# Financial risk protection against noncommunicable diseases: trends and patterns in Bangladesh

**DOI:** 10.1186/s12889-022-14243-0

**Published:** 2022-09-30

**Authors:** Taslima Rahman, Dominic Gasbarro, Khurshid Alam

**Affiliations:** 1 Murdoch Business School, Murdoch University, Perth, WA 6150 Australia; 2 Institute of Health Economics, University of Dhaka, Dhaka, 1000 Bangladesh

**Keywords:** Noncommunicable disease, Out-of-pocket payment, Financial risk protection, Catastrophic health expenditure, Impoverishment, Forgone care, Bangladesh, Low- and middle-income country

## Abstract

**Background:**

Demographic and epidemiological transitions are changing the disease burden from infectious to noncommunicable diseases (NCDs) in low- and middle-income countries, including Bangladesh. Given the rising NCD-related health burdens and growing share of household out-of-pocket (OOP) spending in total health expenditure in Bangladesh, we compared the country’s trends and socioeconomic disparities in financial risk protection (FRP) among households with and without NCDs.

**Methods:**

We used data from three recent waves of the Bangladesh Household Income and Expenditure Survey (2005, 2010, and 2016) and employed the normative food, housing (rent), and utilities method to measure the levels and distributions of catastrophic health expenditure (CHE) and impoverishing effects of OOP health expenditure among households without NCDs (i.e. non-NCDs only) and with NCDs (i.e. NCDs only, and both NCDs and non-NCDs). Additionally, we examined the incidence of forgone care for financial reasons at the household and individual levels.

**Results:**

Between 2005 and 2016, OOP expenses increased by more than 50% across all households (NCD-only: USD 95.6 to 149.3; NCD-and-non-NCD: USD 89.5 to 167.7; non-NCD-only: USD 45.3 to 73.0), with NCD-affected families consistently spending over double that of non-affected households. Concurrently, CHE incidence grew among NCD-only families (13.5% to 14.4%) while declining (with fluctuations) among non-NCD-only (14.4% to 11.6%) and NCD-and-non-NCD households (12.9% to 12.2%). Additionally, OOP-induced impoverishment increased among NCD-only and non-NCD-only households from 1.4 to 2.0% and 1.1 to 1.5%, respectively, affecting the former more. Also, despite falling over time, NCD-affected individuals more frequently mentioned prohibiting treatment costs as the reason for forgoing care than the non-affected (37.9% vs. 13.0% in 2016). The lowest quintile households, particularly those with NCDs, consistently experienced many-fold higher CHE and impoverishment than the highest quintile. Notably, CHE and impoverishment effects were more pronounced among NCD-affected families if NCD-afflicted household members were female rather than male, older people, or children instead of working-age adults.

**Conclusions:**

The lack of FRP is more pronounced among households with NCDs than those without NCDs. Concerted efforts are required to ensure FRP for all families, particularly those with NCDs.

**Supplementary Information:**

The online version contains supplementary material available at 10.1186/s12889-022-14243-0.

## Background

Noncommunicable diseases (NCDs) are a significant health challenge, claiming 41 million lives per year, equivalent to 71% of deaths globally [1]. Low- and middle-income countries (LMICs) are the most affected by NCDs, where 78% of all NCD deaths and 85% of all premature NCD deaths occur [1]. Epidemiological and demographic transitions in LMICs are shifting the disease burden from communicable diseases to NCDs, leaving the countries with a double burden of diseases [2, 3]. Between 2000 and 2019, Disability-Adjusted Life Years (DALYs) lost due to NCDs climbed from 34 to 52% in lower-middle-income countries (LwMICs) and from 20 to 34% in low-income countries (LICs), compared to an increase from 83 to 85% in high-income countries (HICs) [4].

Besides causing premature deaths and disability, NCDs also result in financial hardships for affected individuals and their households, especially in resource-limited LwMICs and LICs [5]. Most LMICs have underdeveloped health systems with inadequate health insurance coverage and insufficient public spending on preventing and treating NCDs [3]. As a result, people must pay for NCD care out-of-pocket (OOP). NCDs are chronic conditions that require protracted and usually expensive care. Consequently, NCD-affected households (i.e., families with members having NCDs) are at a higher risk of experiencing catastrophic and impoverishing OOP expenses than other households [5–10]. Therefore, addressing NCD-related household financial hardships is crucial in combating national and global poverty, improving financial protection, and thus achieving the United Nations Sustainable Development Goals (SDGs) [6].

Bangladesh, a LwMIC in South Asia with a large population of about 165 million, is undergoing epidemiological and demographic transitions and facing high NCD mortality and morbidity [11]. The proportion of deaths due to NCDs in Bangladesh (70%) is higher than the LwMIC average (64%), including the neighboring countries of India (66%), Nepal (66%), and Pakistan (60%) [12]. Troublingly, within a generation or two (by 2040), DALYs lost due to NCDs in Bangladesh are projected to grow exceptionally to more than 80%, to rival its predecessor HICs such as France, Japan, and the US [3]. However, with a health system primarily geared to addressing infectious diseases and maternal and child health problems, the Bangladesh health system is not equipped to tackle the challenges posed by NCDs [13, 14].

As OOP expenses account for a sizable portion of current health expenditure in Bangladesh (currently 73%, up from 61% in 2000), the financial consequences of seeking health care, including NCD care, are substantial [15]. Our previous research on financial risk protection (FRP) against illnesses (all causes) reported a considerable lack of FRP at the national level [16]. Even when using the conservative OOP estimates (derived from a one-year rather than a shorter, mostly 30-day recall period), we found high incidences of catastrophic health expenditure (CHE) (11–14%), impoverishment (over 1%) and further impoverishment (6–9%) during 2005–2016 [16].

Given the rising NCD-related health burdens and increasing share of household spending in total health expenditure in Bangladesh, it is crucial to examine how financially protected NCD-affected households are when seeking health care. However, considering that families deal with all diseases, NCDs and non-NCDs, focusing solely on NCDs will not only lead to disjointed policy suggestions but will also fail to provide an insight into how households manage their members’ competing health care needs. The only nationally representative study investigating NCD-attributable financial risks in Bangladesh found the incidence of CHE among households with and without NCDs was 9.5–13.1% and 7.4%, respectively, and NCD care raised the national poverty rate by 1.37%. [17]. The study analyzed data from 2010, which is now outdated, and did not look into the distribution and trend of these estimates, which is vital for FRP monitoring to be policy-relevant.

Therefore, we analyzed the latest three rounds of nationally representative household survey data to examine the level and distributions of the (lack of) FRP regarding the catastrophic and impoverishing effects of OOP expenses on Bangladeshi households without NCDs (i.e., households affected by non-NCDs only) and with NCDs (i.e., households affected by NCDs only, and both NCDs and non-NCDs). We also measured the incidence of forgone care due to financial constraints as another indicator of the lack of financial protection at both household and individual levels. Previous studies underlined the importance of including the cost barrier to accessing health care when assessing the lack of FRP, pointing out that failing to do so will leave the FRP indicators narrowly conceived [18–21].

Our study broadens the knowledge base of FRP against NCDs in LMICs. Most of the nationally-representative LMIC studies were conducted in China and India, focusing primarily on subgroups of NCD-affected households (such as households with elderly NCD-affected members or those seeking hospitalized NCD care) [22]. Our study is the first to examine trends and patterns of FRP against NCD and non-NCD care in Bangladesh on a nationally representative scale. We covered NCDs not previously studied, including digestive and musculoskeletal diseases, which were the most common NCDs during the study period [23–25]. Unlike prior LMIC research, ours included estimates of two critical but frequently ignored FRP indicators, further impoverishment and forgone care due to financial constraints, providing a comprehensive picture of the lack of FRP against NCD and non-NCD care. Notably, we verified all results using alternative approaches, accounting for the large discrepancy in OOP expenses from the household survey’s ‘health’ and ‘consumption’ modules.

The findings of this study will guide policies and legislation to protect families from the adverse financial consequences of illness in LMICs in general and Bangladesh in particular, the implementation of which would contribute to poverty alleviation and the achievement of the SDGs in the countries.

## Methods

### Data source

Data for this study comes from the three recent waves (2005, 2010, and 2016) of the Bangladesh Household Income and Expenditure Survey (HIES), conducted on 10,080, 12,240, and 46,076 households, respectively [23–25]. Bangladesh HIES is a nationally representative, repeated cross-sectional survey undertaken approximately every five years by the Bangladesh Bureau of Statistics to monitor the population’s living standards and poverty levels. HIES 2005 and 2010 rounds employed a two-stage stratified random sampling method, while HIES 2016 used a stratified two-stage cluster sampling technique. Both the ‘consumption’ and ‘health’ modules of each HIES round contain data on OOP payments. The former collects household-level OOP expenses with a 12-month recall period. The latter gathers the same at the individual level using a 30-day recall period (except for a 12-month recall period for inpatient care in 2016). Consistent with earlier studies, annual OOP expenses from the shorter recall period (health module) were higher than those from the longer recall period (consumption module) [26, 27]. The health module provides additional information on illness occurrence, care-seeking behavior, and the reasons if ill individuals forgo care.

HIES inquired if individuals in the household had any chronic illness in the previous 12 months and any diseases/symptoms (including chronic diseases) in the 30 days before the survey. In the case of a positive response, they were asked to name the disease(s) in order of importance: two for the 12-month question (except just one in 2005) and three for the 30-day question. The complete list of conditions varied slightly among the three HIES rounds. To ensure a valid comparison, we only considered NCDs and non-NCDs that were common throughout the three waves. Cancer, diabetes, heart diseases, hypertension, respiratory diseases (asthma), musculoskeletal diseases (arthritis/rheumatism), digestive diseases (gastric/ulcer), paralysis, and skin diseases are the common chronic NCDs. The non-NCDs include diarrhoeal diseases, dizziness, weakness, fever, jaundice, malaria, pneumonia, tuberculosis, and typhoid. Given the secondary nature of the data used, the Human Research Ethics Committee of Murdoch University, Australia, granted an ethics waiver for this study (reference no. 2020/202).

### Data analysis

Depending on the presence of NCDs or non-NCDs in a household, we put it into one of three groups: households having members with non-NCDs only, NCDs only, and both NCDs and non-NCDs. We compared households with and without NCDs in terms of annual average OOP expenses, CHE, and impoverishment incidences. We also examined the distribution of these indicators across selected equity strata: consumption quintile, area of residence, household head’s education, illness of the household’s main income earner (defined as illness of the household head who is also an earner), age and gender composition of ill household members, comorbidity, and the number of ailing household members. Additionally, we compared the incidence of forgone care for financial (and other) reasons at the household and individual levels.

We used the conservative measure of annual OOP expenses from the consumption module as a separate variable and as a component of total consumption expenditure (thus, CTP). Alternative calculations (two other approaches) using annualized OOP expenses from the health module and a combination of the health and consumption module are presented in additional files. Details of the alternative formulations are in Additional file 1. All expenditures in Bangladeshi taka (BDT) were expressed in 2016 prices using the consumer price index (CPI) and then converted into US dollars using the average 2016 exchange rate (BDT 78.468 = USD 1) [28, 29]. The household-level results are survey estimates generated by the survey commands of Stata (version 17.0).

We applied the normative capacity-to-pay (CTP) method developed by the World Health Organization’s (WHO) Regional Office for Europe to measure CHE and impoverishment incidences. The method’s specifics, including comparisons to conventional measurement methods and equity implications, are explained elsewhere [30–32]. This method is currently being used to monitor FRP in Europe, including in countries with LMIC status [32–35]. In this method, a household’s CTP for health care is measured as total consumption expenses minus subsistence expenditure (SE). SE is defined as per capita total spending after deducting an estimated amount for basic needs (average expenditure on food, housing (rent), and utilities (gas/fuel, electricity, water) between the 25^th^ and 35^th^ percentiles of adult equivalent total consumption expenditure per capita). We excluded tobacco and tobacco-related consumption and dining out while calculating basic food spending; considered paid rent for rented accommodation and imputed rent for owner-occupied dwellings; and used the standard WHO household equivalence scale to derive per capita expenses [36].

#### Catastrophic health expenditure

OOP expenses are catastrophic if a household spends 40% or more of its CTP on health care. Furthermore, health expenditure by “poor” households (those with total consumption expenditure less than their SE and, thus, having a negative CTP) is considered catastrophic in this normative approach. Since OOP expenses are measured relative to CTP, the effective threshold in CHE measurement is lower for poorer households and higher for wealthier families. For comparison, we also examined the level and distribution of CHE incidence by applying the budget-share method at the 10% threshold (the official indicator to measure FRP in the SDGs) [37].

#### Impoverishment effects

To find the impoverishment effects of OOP payments, we compared total household consumption expenditure gross and net of OOP expenses. We then divided all households into the following five mutually exclusive categories according to their risk of impoverishment [30, 33]:

1. *Further impoverished*: Already poor households whose poverty conditions were aggravated by OOP expenses. These households’ total (consumption) expenditure was already below SE, so net spending was even lower.
2. *Impoverished:* Non-poor households who fell into poverty due to OOP expenses. These households’ total expenditure was higher than SE, but net spending was lower.
3. *At-risk of impoverishment:* Non-poor households that were not impoverished but became near-poor due to OOP expenses. Both total and net expenditures were higher than SE. However, the latter was very close (within 120%) to SE [30, 33].
4. *Not at-risk of impoverishment:* Non-poor households that were not impoverished or did not become near-poor due to OOP expenses. Total and net expenditure was higher than (120% of) SE [30, 33].
5. *Non-spender:* Households that did not spend on health care. With zero OOP expenses, total and net expenditures were the same.

To identify the households that forgo care due to financial constraints, we disaggregated the non-spenders by reasons into the following mutually exclusive categories:

- 5a. *Financial reasons*
- *5b. Non-financial reasons*
- *5c. Unspecified reasons*
- *5d. Non-spender but sought health care*

Each category’s definition, including how we converted individual-level information on forgone care to household-level, is available in Additional file 2. Finally, to assess foregone care at the individual level, we grouped individuals who did not seek care for their ailment within 30 days before the survey based on their reasons for not seeking treatment.

## Results

Table 1 shows descriptive statistics of households with NCDs only, non-NCDs only, and both NCDs and non-NCDs. During the study period, the proportion of households with NCDs increased (NCD-only: from 16.4% in 2005 to 20.0% in 2010 to 20.4% in 2016; both NCDs and non-NCDs: 17.9% in 2005 to 19.9% in 2010 to 22.1% in 2016), whereas that of families without NCDs declined (non-NCD-only: from 28.5% in 2005 to 24.0% in 2010 to 22.6% in 2016). The increase in NCD prevalence was the highest among the lowest quintile families, increasing from 16.2% in 2005 to 19.1% in 2016 among NCD-only families. Despite this, most NCD-affected households were in the wealthiest quintile throughout the study period (around 22–26% vs. 15–19% in the lowest), while most without NCDs were in the lowest (approximately 21–23% vs. 15–18% in the highest). Additionally, the largest proportion of unwell people comprised working-age adults among NCD-only households (63.0–68.0%) and children under 18 among non-NCD-only families (39.0–45.0%). Over time, comorbidity increased across all households, more dramatically among families with NCDs (NCD-only: 2.4% to 29.7%, NCD-and-non-NCD: 54.8% to 73.5%) compared to those without NCDs (16.6% to 23.2%).

**Table 1:** Background characteristics of households affected by NCD only, non-NCD only, and both NCD and non-NCD (%)

	Households affected by non-NCD only			Households affected by NCD only			Households affected by both NCD & non-NCD		
	2005 (*n* = 2,875)	2010 (*n* = 2,931)	2016 (*n* = 10,391)	2005 (*n* = 1,648)	2010 (*n* = 2, 449)	2016 (*n* = 9,393)	2005 (*n* = 1,806)	2010 (*n* = 2,440)	2016 (*n* = 10,160)
Overall	28.5 (0.5)	24.0 (0.6)	22.6 (0.5)	16.4 (0.4)	20.0 (0.5)	20.4 (0.4)	17.9 (0.4)	19.9 (0.7)	22.1 (0.4)
Consumption expenditure quintile									
Lowest	21.3 (0.8)	23.1 (1.1)	21.5 (0.9)	16.2 (0.9)	17.0 (0.9)	19.1 (0.7)	15.9 (0.9)	16.3 (1.0)	15.3 (0.6)
2nd	22.6 (0.8)	22.7 (0.9)	21.2 (0.8)	17.9 (1.0)	16.5 (0.8)	19.2 (0.6)	17.3 (0.9)	19.0 (1.0)	18.6 (0.6)
3rd	20.8 (0.8)	19.9 (0.8)	20.1 (0.7)	18.9 (1.0)	19.0 (0.9)	19.9 (0.6)	20.4 (1.0)	20.7 (0.9)	19.4 (0.6)
4th	18.6 (0.8)	19.0 (0.9)	19.9 (1.0)	20.6 (1.1)	21.3 (1.0)	19.1 (0.7)	21.3 (1.1)	21.6 (1.0)	21.3 (0.6)
Highest	16.6 (0.7)	15.4 (1.0)	17.5 (1.0)	26.4 (1.1)	26.3 (1.3)	22.7 (0.8)	25.1 (1.0)	22.4 (1.1)	25.4 (1.2)
Area of residence									
Rural	78.0 (0.0)	78.9 (1.0)	70.0 (1.7)	72.1 (0.0)	69.0 (1.0)	74.2 (1.1)	76.2 (0.0)	81.3 (1.0)	75.6 (1.1)
Urban	22.0 (0.0)	21.1 (1.0)	30.0 (1.7)	27.9 (0.0)	31.0 (1.0)	25.8 (1.1)	23.8 (0.0)	18.7 (1.0)	24.4 (1.1)
Household head’s education									
No education	57.3 (1.0)	53.5 (1.2)	40.4 (0.9)	52.1 (1.3)	50.9 (1.3)	43.8 (0.8)	54.1 (1.2)	54.0 (1.2)	42.0 (0.9)
Below secondary	29.8 (0.9)	33.2 (1.1)	46.0 (0.8)	30.5 (1.2)	29.5 (1.1)	39.6 (0.7)	31.1 (1.2)	31.9 (1.1)	43.5 (0.8)
Secondary or above	12.9 (0.7)	13.4 (0.8)	13.6 (0.8)	17.4 (1.0)	19.6 (1.3)	16.6 (0.8)	14.7 (0.9)	14.1 (0.8)	14.5 (0.8)
Illness of main income earner									
No	76.0 (0.9)	72.9 (1.0)	74.6 (0.7)	56.6 (1.3)	57.7 (1.2)	58.4 (0.8)	42.9 (1.2)	44.8 (1.1)	46.6 (0.8)
Yes	24.0 (0.9)	27.1 (1.0)	25.4 (0.7)	43.4 (1.3)	42.3 (1.2)	41.6 (0.8)	57.1 (1.2)	55.2 (1.1)	53.4 (0.8)
Age composition of ill members									
Children (< 18 years) only	44.9 (1.0)	38.5 (1.1)	39.6 (0.7)	5.5 (0.6)	4.9 (0.5)	4.2 (0.4)	4.0 (0.5)	2.1 (0.3)	4.5 (0.5)
Non-elderly adults (18–60 years) only	32.6 (1.0)	38.3 (1.0)	35.5 (0.8)	68.3 (1.2)	65.5 (1.1)	63.3 (0.7)	29.3 (1.2)	29.6 (1.1)	31.3 (0.7)
Elderly (> 60 years) only	6.0 (0.5)	4.6 (0.4)	4.1 (0.3)	16.9 (1.0)	19.2 (1.0)	20.1 (0.7)	4.6 (0.6)	7.3 (0.6)	6.8 (0.4)
Children and non-elderly adults	14.5 (0.7)	16.7 (0.8)	19.0 (0.6)	2.8 (0.5)	3.3 (0.4)	3.1 (0.2)	45.3 (1.3)	44.5 (1.2)	42.5 (0.8)
Non-elderly adults and elderly	0.9 (0.2)	1.3 (0.2)	1.0 (0.1)	6.4 (0.6)	6.8 (0.6)	8.9 (0.4)	10.8 (0.8)	11.9 (0.8)	10.3 (0.4)
Children and elderly	1.0 (0.2)	0.6 (0.1)	0.8 (0.1)	0.0 (0.0)	0.3 (0.1)	0.3 (0.1)	6.0 (0.6)	4.5 (0.5)	4.6 (0.3)
Gender composition of ill members									
Male only	40.0 (1.0)	38.5 (1.1)	34.1 (0.8)	37.3 (1.3)	32.9 (1.1)	29.6 (0.6)	16.5 (0.9)	12.8 (0.8)	12.0 (0.5)
Female only	42.4 (1.0)	41.2 (1.1)	43.2 (0.8)	43.6 (1.3)	43.7 (1.2)	44.0 (0.7)	19.1 (1.0)	21.3 (0.9)	22.2 (0.6)
Male and female	17.6 (0.8)	20.3 (0.9)	22.6 (0.7)	19.1 (1.0)	23.3 (1.1)	26.5 (0.7)	64.4 (1.2)	65.9 (1.1)	65.8 (0.8)
Number of ill members									
One	71.4 (0.9)	68.6 (1.0)	66.3 (0.8)	77.3 (1.1)	73.3 (1.2)	70.2 (0.7)	15.1 (0.9)	15.6 (0.8)	17.8 (0.7)
Two or more	28.6 (0.9)	31.4 (1.0)	33.7 (0.8)	22.7 (1.1)	26.7 (1.2)	29.8 (0.7)	84.9 (0.9)	84.4 (0.8)	82.2 (0.7)
Comorbidity of ill members									
One disease (no comorbidity)	83.4 (0.7)	90.7 (0.8)	76.8 (1.2)	97.6 (0.4)	80.2 (1.0)	70.3 (0.8)	45.2 (1.3)	36.5 (1.3)	26.5 (0.7)
Two or more diseases	16.6 (0.7)	9.3 (0.8)	23.2 (1.2)	2.4 (0.4)	19.8 (1.0)	29.7 (0.8)	54.8 (1.3)	63.5 (1.3)	73.5 (0.7)

Numbers in parentheses are standard errors

Total number of households included in analysis: 10,075 in 2005, 12,237 in 2010, and 45,976 in 2016

*NCD* Noncommunicable diseases

During the study period, all households experienced more than 50% increase in annual OOP expenses (Table 2), with families having NCDs spending around twice as much as those without NCDs each year (in 2005, 2010, and 2016, NCD-only: USD 95.6, USD 120.8, and USD 149.3, respectively; NCD-and-non-NCD: USD 89.5, USD 161.6, and USD 167.7, respectively; non-NCD-only: USD 45.3, USD 68.3, and USD 73.0, respectively). NCD-affected families in the wealthiest quintile spent seven to ten times more than the lowest quintile (e.g., USD 325.6 vs. USD 42.2 in 2016 among NCD-only households) compared to five to six times more in non-affected homes (e.g., USD 139.2 vs. USD 28.0 in 2016). OOP expenses were also higher among households in urban than rural areas (e.g., with NCD: 43–69% higher, without NCD: 25% higher in 2016) and among those with heads having secondary or above literacy than none (e.g., with NCD: 123–128% higher, without NCD: 45% higher in 2016); still, the discrepancy was more notable for those with NCDs than those without NCDs. The illness of the family’s primary income earner had little effect on OOP expenses for households without NCDs (illness of main income earner vs. other household members: USD 46.7 vs. 44.9 in 2005, USD 69.8 vs. 67.8 in 2010, and USD 72.0 vs. 73.4 in 2016). However, those with both NCDs and non-NCDs consistently had lower OOP expenses (USD 77.7 vs. 105.3 in 2005, USD 130.4 vs. 200.1 in 2010, and USD 166.9 vs. 168.7 in 2016).

**Table 2:** Annual average household-level out-of-pocket expenditure (in USD^a^)

	Households affected by non-NCD only			Households affected by NCD only			Households affected by both NCD & non-NCD		
	2005 (*n* = 2,875)	2010 (*n* = 2,931)	2016 (*n* = 10,391)	2005 (*n* = 1,648)	2010 (*n* = 2, 449)	2016 (*n* = 9,393)	2005 (*n* = 1,806)	2010 (*n* = 2,440)	2016 (*n* = 10,160)
Overall	45.3 (1.7)	68.3 (3.4)	73.0 (2.2)	95.6 (8.3)	120.8 (8.7)	149.3 (4.9)	89.5 (4.6)	161.6 (28.5)	167.7 (5.8)
Consumption expenditure quintile									
Lowest	15.9 (0.7)	29.7 (1.7)	28.0 (1.1)	24.3 (2.4)	28.7 (1.8)	42.2 (1.5)	27.4 (2.1)	39.5 (2.2)	44.8 (1.8)
2nd	31.7 (1.7)	42.6 (2.5)	46.0 (1.6)	37.1 (2.3)	50.6 (3.7)	72.5 (2.9)	43.1 (2.8)	68.4 (4.0)	82.5 (2.8)
3rd	39.9 (2.5)	66.6 (3.9)	66.5 (2.9)	54.3 (4.0)	76.3 (5.2)	109.6 (4.2)	54.1 (3.1)	94.4 (5.5)	119.3 (4.6)
4th	58.6 (3.6)	78.5 (5.3)	98.9 (4.4)	97.4 (8.4)	109.8 (7.6)	166.0 (6.4)	82.6 (4.7)	137.6 (7.7)	168.9 (5.9)
Highest	93.7 (8.0)	153.8 (16.5)	139.2 (8.7)	206.9 (29.8)	265.3 (29.8)	325.6 (16.5)	195.7 (16.5)	414.6 (123.9)	340.4 (16.0)
Area of residence									
Rural	41.8 (1.6)	67.1 (3.6)	67.9 (2.7)	79.3 (5.1)	104.0 (6.7)	126.8 (4.3)	78.2 (4.0)	135.0 (7.2)	151.8 (4.7)
Urban	57.9 (5.3)	72.9 (8.6)	85.1 (4.0)	137.6 (26.7)	158.2 (23.8)	214.3 (14.2)	125.6 (14.4)	277.5 (149.9)	217.1 (17.7)
Household head’s education									
No education	38.2 (1.5)	56.6 (3.3)	60.5 (2.6)	65.2 (4.7)	90.9 (7.4)	107.9 (4.4)	72.0 (4.8)	113.0 (6.6)	126.3 (4.7)
Below secondary	50.4 (4.1)	78.3 (6.1)	79.6 (3.5)	88.2 (8.1)	115.4 (9.7)	154.6 (5.7)	102.2 (11.3)	140.9 (9.9)	169.6 (6.8)
Secondary or above	65.2 (6.3)	90.7 (12.9)	88.2 (4.5)	199.1 (42.6)	206.8 (30.0)	246.1 (18.8)	127.2 (10.2)	395.3 (196.3)	282.0 (20.3)
Illness of main income earner									
No	44.9 (1.9)	67.8 (3.5)	73.4 (2.5)	90.4 (8.6)	119.2 (7.8)	152.1 (6.4)	105.3 (9.0)	200.1 (62.4)	168.7 (7.0)
Yes	46.7 (3.9)	69.8 (6.9)	72.0 (3.4)	102.3 (15.5)	123.0 (17.2)	145.5 (6.3)	77.7 (4.5)	130.4 (7.2)	166.9 (6.9)
Age composition of ill members									
Children (< 18 years) only	44.5 (2.3)	57.1 (3.3)	66.9 (3.0)	53.1 (8.3)	67.7 (9.1)	116.4 (16.5)	55.4 (9.1)	157.3 (57.2)	84.8 (11.5)
Non-elderly adults (18–60 years) only	41.8 (2.5)	66.5 (4.3)	72.4 (3.5)	94.5 (11.5)	124.8 (12.4)	141.0 (6.3)	83.2 (6.5)	114.3 (8.5)	152.5 (7.2)
Elderly (> 60 years) only	41.5 (5.1)	83.6 (22.0)	56.6 (5.1)	97.6 (12.6)	110.4 (12.8)	151.9 (8.7)	141.7 (68.5)	101.8 (13.8)	147.3 (13.1)
Children and non-elderly adults	57.0 (7.1)	88.1 (11.2)	85.9 (4.3)	109.4 (36.2)	128.9 (28.3)	156.6 (17.6)	79.2 (5.2)	133.0 (8.2)	160.8 (6.3)
Non-elderly adults and elderly	53.8 (11.3)	79.6 (21.2)	72.8 (9.3)	130.3 (20.0)	146.4 (16.6)	209.5 (12.0)	108.1 (13.7)	156.7 (18.7)	214.2 (23.2)
Children and elderly	43.5 (10.0)	118.2 (38.5)	96.8 (19.7)	12.8 (2.0)	121.4 (43.1)	200.9 (41.9)	126.0 (23.5)	156.5 (28.4)	200.3 (26.8)
Gender composition of ill members									
Male only	46.4 (3.1)	61.0 (3.5)	67.5 (3.1)	122.6 (20.8)	133.6 (18.3)	145.8 (7.7)	77.3 (10.0)	108.6 (13.7)	138.2 (9.8)
Female only	38.6 (1.8)	65.2 (4.7)	70.0 (2.9)	66.4 (5.0)	108.2 (8.3)	128.9 (6.7)	107.9 (17.7)	99.8 (8.1)	127.5 (6.8)
Male and female	59.0 (5.3)	88.5 (9.8)	87.2 (4.4)	109.4 (10.1)	126.4 (11.2)	187.1 (8.6)	87.2 (4.2)	191.9 (42.7)	186.7 (7.4)
Number of ill members									
One	41.5 (1.7)	63.9 (3.4)	67.1 (2.5)	91.7 (10.4)	117.5 (9.6)	132.4 (5.5)	92.0 (20.9)	92.1 (10.4)	116.8 (8.8)
Two or more	54.9 (4.3)	78.0 (6.7)	84.8 (3.5)	108.8 (9.4)	130.1 (10.2)	189.2 (8.1)	89.1 (4.0)	174.5 (33.6)	178.8 (6.5)
Comorbidity of ill members									
One disease (no comorbidity)	44.2 (1.8)	67.5 (3.5)	72.9 (2.4)	95.0 (8.5)	113.2 (8.7)	138.6 (5.6)	87.7 (5.4)	195.7 (76.6)	151.0 (7.1)
Two or more diseases	51.3 (5.1)	76.7 (11.2)	73.4 (4.0)	119.4 (24.1)	151.7 (16.0)	174.8 (7.8)	91.0 (7.1)	142.0 (7.8)	173.8 (6.9)

Numbers in parentheses are standard errors

*NCD* Noncommunicable diseases, *OOP* Out-of-pocket

^a^All expenses in Bangladeshi taka (BDT) were expressed in 2016 prices using consumer price index, CPI (CPI_2005_ = 69.153, CPI_2010_ = 100, and CPI_2016_ = 152.529) and then converted into US dollars using the 2016 average exchange rate (USD 1 = BDT 78.468)

The mean CHE incidence using the normative food, rent, and utilities method (Table 3) increased steadily among NCD-only families during the study period (from 13.5% to 13.7% to 14.4% in 2005, 2010, and 2016, respectively). However, it declined (with fluctuations) among the other two household categories (non-NCD-only: from 14.4% to 15.7% to 11.6% in 2005, 2010, and 2016; both NCDs and non-NCDs: from 12.9% to 13.5% to 12.2% in 2005, 2010, and 2016, respectively). Despite a decline over time, OOP expenses were catastrophic for around half of the lowest quintile households in 2016 (households with NCDs: about 58.0%, without NCDs: 49.0%). As only a minuscule proportion of the wealthiest families experienced CHE throughout the study period (with NCD: 1.0–2.0%, without NCD: 0.0–0.8%), there was a persistently wide disparity in CHE incidence between the lowest and highest quintile households, more so among NCD-affected families. CHE was higher in rural than urban areas among all three household types, with the rural NCD-only households incurring higher CHE (17.0%) in 2016 than those without NCDs (14.4%).

**Table 3:** Incidence of Catastrophic health expenditure (%), normative food, housing (rent), and utilities method, 40% threshold

	Households affected by non-NCD only			Households affected by NCD only			Households affected by both NCD & non-NCD		
	2005 (*n* = 2,875)	2010 (*n* = 2,931)	2016 (*n* = 10,391)	2005 (*n* = 1,648)	2010 (*n* = 2, 449)	2016 (*n* = 9,393)	2005 (*n* = 1,806)	2010 (*n* = 2,440)	2016 (*n* = 10,160)
Overall	14.4 (0.7)	15.7 (1.0)	11.6 (0.6)	13.5 (0.9)	13.7 (0.8)	14.4 (0.5)	12.9 (0.8)	13.5 (0.8)	12.2 (0.5)
Consumption expenditure quintile									
Lowest	63.3 (2.1)	62.0 (2.4)	48.9 (1.4)	68.3 (3.0)	68.5 (2.3)	57.7 (1.5)	69.9 (2.9)	64.9 (2.4)	57.9 (1.6)
2nd	2.8 (0.7)	3.4 (0.8)	2.6 (0.4)	7.3 (1.6)	6.5 (1.4)	8.3 (0.8)	6.8 (1.5)	9.2 (1.4)	8.3 (0.7)
3rd	0.9 (0.4)	1.8 (0.6)	1.3 (0.4)	2.2 (0.9)	1.8 (0.6)	4.2 (0.6)	1.9 (0.7)	2.4 (0.8)	3.9 (0.5)
4th	0.5 (0.3)	0.6 (0.4)	0.7 (0.2)	2.1 (0.8)	1.7 (0.6)	3.0 (0.5)	0.0 (n/o)	1.1 (0.5)	2.3 (0.4)
Highest	0.0 (n/o)	0.8 (0.4)	0.8 (0.2)	1.2 (0.6)	1.0 (0.5)	1.8 (0.3)	1.0 (0.5)	2.0 (0.7)	1.9 (0.4)
Area of residence									
Rural	16.8 (0.8)	18.1 (1.2)	14.4 (0.7)	16.5 (1.1)	17.8 (1.1)	17.0 (0.7)	15.1 (1.0)	14.9 (1.0)	14.0 (0.6)
Urban	5.9 (0.6)	6.7 (0.9)	5.1 (0.7)	5.7 (0.8)	4.3 (0.6)	7.0 (0.7)	6.2 (0.8)	7.5 (1.2)	6.3 (0.8)
Household head’s education									
No education	20.5 (1.0)	21.0 (1.3)	17.1 (0.9)	20.9 (1.4)	21.7 (1.3)	21.3 (0.9)	19.3 (1.3)	19.6 (1.3)	16.4 (0.8)
Below secondary	8.5 (1.0)	11.5 (1.2)	9.6 (0.7)	7.2 (1.2)	8.2 (1.1)	11.3 (0.6)	7.1 (1.1)	6.5 (1.0)	10.7 (0.6)
Secondary or above	1.1 (0.5)	4.8 (1.1)	2.0 (0.4)	2.5 (1.1)	1.0 (0.4)	4.0 (0.6)	1.9 (0.9)	5.9 (1.4)	4.2 (0.7)
Illness of main income earner									
No	13.1 (0.7)	15.5 (1.1)	10.8 (0.6)	14.6 (1.2)	14.3 (1.0)	15.7 (0.7)	11.9 (1.2)	14.8 (1.2)	11.7 (0.7)
Yes	18.8 (1.6)	16.2 (1.6)	13.9 (1.0)	12.1 (1.3)	12.8 (1.2)	12.6 (0.7)	13.7 (1.1)	12.4 (1.1)	12.6 (0.7)
Age composition of ill members									
Children (< 18 years) only	13.6 (1.0)	17.5 (1.4)	10.4 (0.7)	16.2 (4.1)	18.3 (3.7)	18.4 (3.1)	13.7 (4.5)	20.7 (6.1)	14.7 (2.3)
Non-elderly adults (18–60 years) only	14.5 (1.2)	14.5 (1.3)	11.5 (0.8)	12.0 (1.0)	12.0 (0.9)	11.9 (0.6)	11.6 (1.5)	13.7 (1.4)	12.2 (0.9)
Elderly (> 60 years) only	21.5 (3.4)	26.2 (4.5)	23.8 (2.5)	20.2 (2.5)	19.8 (2.1)	24.5 (1.4)	21.2 (4.9)	28.0 (3.8)	29.3 (2.4)
Children and non-elderly adults	14.9 (1.9)	12.0 (1.6)	11.5 (1.0)	16.2 (6.1)	10.5 (3.6)	8.6 (1.6)	13.0 (1.3)	12.6 (1.2)	10.4 (0.7)
Non-elderly adults and elderly	1.8 (1.8)	14.4 (5.9)	13.9 (3.5)	9.1 (2.9)	11.2 (2.6)	10.7 (1.2)	16.5 (2.9)	8.5 (1.9)	12.2 (1.3)
Children and elderly	12.9 (6.9)	0.0 (n/o)	13.2 (5.2)	0.0 (n/o)	18.7 (13.1)	0.0 (n/o)	13.0 (3.6)	10.3 (3.0)	6.2 (1.3)
Gender composition of ill members									
Male only	14.7 (1.1)	16.6 (1.4)	11.2 (0.8)	13.5 (1.4)	13.1 (1.4)	13.8 (0.9)	17.2 (2.3)	12.7 (2.1)	14.4 (1.3)
Female only	15.0 (1.1)	16.8 (1.5)	12.1 (0.8)	15.9 (1.4)	16.0 (1.2)	17.3 (0.9)	17.6 (2.2)	18.6 (1.9)	15.7 (1.1)
Male and female	12.3 (1.6)	11.8 (1.4)	11.0 (0.9)	8.0 (1.6)	10.1 (1.4)	10.4 (0.7)	10.5 (0.9)	12.0 (1.0)	10.6 (0.5)
Number of ill members									
One	14.9 (0.8)	16.8 (1.2)	11.9 (0.7)	14.7 (1.0)	15.2 (0.9)	16.1 (0.7)	17.4 (2.4)	23.0 (2.4)	19.5 (1.4)
Two or more	13.3 (1.3)	13.4 (1.3)	11.0 (0.8)	9.5 (1.6)	9.4 (1.3)	10.4 (0.7)	12.2 (0.9)	11.8 (0.9)	10.6 (0.5)
Comorbidity of ill members									
One disease (no comorbidity)	13.5 (0.7)	15.8 (1.0)	11.5 (0.6)	13.7 (0.9)	14.5 (0.9)	15.2 (0.6)	10.7 (1.1)	12.2 (1.3)	11.3 (0.8)
Two or more diseases	19.1 (1.9)	14.4 (2.5)	11.8 (1.1)	6.1 (4.2)	10.3 (1.6)	12.6 (0.8)	14.8 (1.2)	14.3 (1.1)	12.4 (0.6)

Numbers in parentheses are standard errors

*NCD* Noncommunicable diseases, *OOP* Out-of-pocket, *n/o* No observations

All families, with or without NCD, incurred the highest CHE if it was only the older people who were ill (with NCD: 24.5–29.3%, without NCD: 23.8% in 2016). Additionally, among NCD-affected households, CHE was higher if all illness-afflicted members were children (under 18 years) than if they were of productive age (e.g., NCD-only: 18.4% vs. 11.9%, NCD-and-non-NCD: 14.7% vs. 12.2% in 2016) or when only female family members were ill than when only males were sick (e.g., NCD-only: 17.3% vs. 13.8%, NCD-and-non-NCD: 15.7% vs. 14.4% in 2016). Contrary to expectations, CHE incidence was lower among all families with more ill members than just one. Furthermore, NCD-only households had lower CHE if the afflicted individual had more than one NCD than only one.

The budget share method showed a consistent upward trend in the mean CHE incidence (Table 4) among all households over the study period, regardless of NCD presence, with the incidence being over twofold higher among families with NCDs than those without NCDs (in 2005, 2010, and 2016: NCD-only: 7.7%, 8.3%, 16.6%, respectively; NCD-and-non-NCD: 6.3%, 9.4%, 15.6%, respectively; non-NCD-only: 3.1%, 4.6%, 5.6%, respectively). A roughly similar trend prevailed across all equity strata, including households’ residence locations. However, in contrast to the normative food, rent, and utilities method, CHE increased with household economic status, particularly among NCD-affected families. For example, among NCD-only families, CHE was 7.7%, 10.9%, and 18.2% in 2005, 2010, and 2016, respectively, among the wealthiest quintile compared to the corresponding 5.7%, 5.1%, and 15.3% among the lowest quintile families. Moreover, CHE grew in all households when multiple people were ill or at least one individual had a comorbid condition.

**Table 4:** Incidence of catastrophic health expenditure (%), budget share method, 10% threshold

	Households affected by non-NCD only			Households affected by NCD only			Households affected by both NCD & non-NCD		
	2005 (*n* = 2,875)	2010 (*n* = 2,931)	2016 (*n* = 10,391)	2005 (*n* = 1,648)	2010 (*n* = 2, 449)	2016 (*n* = 9,393)	2005 (*n* = 1,806)	2010 (*n* = 2,440)	2016 (*n* = 10,160)
Overall	3.1 (0.4)	4.6 (0.5)	5.6 (0.3)	7.7 (0.7)	8.3 (0.7)	16.6 (0.7)	6.3 (0.6)	9.4 (0.7)	15.6 (0.6)
Consumption expenditure quintile									
Lowest	1.1 (0.4)	3.5 (0.7)	5.5 (0.6)	5.7 (1.5)	5.1 (1.2)	15.3 (1.1)	4.5 (1.4)	4.4 (1.1)	12.6 (1.0)
2nd	2.6 (0.7)	3.1 (0.8)	5.1 (0.5)	7.1 (1.6)	6.5 (1.5)	14.0 (1.1)	5.9 (1.4)	9.1 (1.3)	14.2 (1.0)
3rd	3.7 (0.8)	7.0 (1.1)	6.3 (0.7)	6.3 (1.5)	8.5 (1.5)	16.7 (1.7)	6.1 (1.3)	7.8 (1.6)	17.2 (1.3)
4th	4.2 (1.0)	4.9 (1.0)	6.2 (0.9)	10.9 (1.9)	8.9 (1.5)	18.5 (1.5)	6.0 (1.3)	10.9 (1.4)	15.9 (1.2)
Highest	4.4 (1.1)	5.3 (1.2)	4.8 (0.7)	7.7 (1.4)	10.9 (1.5)	18.2 (1.4)	8.3 (1.5)	13.4 (1.5)	17.1 (1.3)
Area of residence									
Rural	3.1 (0.4)	5.0 (0.5)	5.7 (0.4)	7.9 (0.9)	8.6 (0.9)	16.7 (0.9)	6.1 (0.7)	10.0 (0.8)	16.0 (0.7)
Urban	3.2 (0.8)	3.3 (1.1)	5.3 (0.7)	7.1 (1.3)	7.6 (1.3)	16.5 (1.1)	7.2 (1.4)	7.0 (1.0)	14.4 (1.2)
Household head’s education									
No education	2.9 (0.5)	4.2 (0.6)	5.8 (0.5)	7.5 (1.0)	9.2 (1.0)	16.2 (1.0)	6.7 (0.9)	9.3 (0.9)	14.2 (0.8)
Below secondary	3.4 (0.7)	5.8 (0.9)	5.8 (0.5)	7.9 (1.3)	7.7 (1.2)	17.7 (0.9)	6.3 (1.1)	8.3 (1.0)	16.5 (0.9)
Secondary or above	3.1 (1.0)	3.4 (1.0)	4.2 (0.7)	7.6 (1.7)	6.9 (1.3)	15.0 (1.2)	5.0 (1.5)	12.5 (1.9)	17.0 (1.5)
Illness of main income earner									
No	2.6 (0.4)	4.6 (0.6)	5.4 (0.4)	7.4 (0.9)	9.4 (0.9)	17.2 (0.9)	7.9 (1.1)	9.9 (0.9)	15.2 (0.8)
Yes	4.6 (0.9)	4.7 (0.8)	6.2 (0.6)	7.9 (1.1)	6.8 (1.0)	15.8 (0.9)	5.2 (0.8)	9.1 (0.9)	16.0 (0.7)
Age composition of ill members									
Children (< 18 years) only	2.6 (0.5)	3.6 (0.6)	4.5 (0.4)	8.2 (3.5)	4.2 (1.9)	20.9 (6.7)	1.4 (1.4)	11.5 (4.8)	8.4 (1.8)
Non-elderly adults (18–60 years) only	3.2 (0.6)	4.7 (0.7)	6.0 (0.5)	7.4 (0.9)	8.4 (0.9)	14.0 (0.7)	8.0 (1.3)	7.8 (1.2)	13.9 (0.9)
Elderly (> 60 years) only	3.7 (1.6)	7.3 (2.5)	6.6 (1.3)	9.0 (1.8)	9.5 (1.5)	22.1 (1.5)	13.0 (4.3)	14.5 (2.9)	25.2 (2.2)
Children and non-elderly adults	4.3 (1.2)	6.1 (1.2)	6.6 (0.7)	2.6 (2.6)	7.8 (3.9)	14.8 (2.5)	5.3 (0.9)	8.6 (0.9)	14.3 (1.0)
Non-elderly adults and elderly	3.8 (3.7)	5.9 (4.1)	6.4 (2.6)	9.1 (2.9)	7.1 (2.0)	21.1 (1.7)	6.1 (1.9)	10.6 (1.9)	20.8 (1.6)
Children and elderly	0.0 (n/o)	0.0 (n/o)	5.6 (2.6)	0.0 (n/o)	11.0 (10.4)	18.4 (7.6)	8.7 (3.0)	9.1 (2.9)	16.5 (2.2)
Gender composition of ill members									
Male only	3.4 (0.6)	4.3 (0.7)	5.2 (0.5)	10.9 (1.4)	7.8 (1.1)	17.6 (1.8)	6.6 (1.6)	6.7 (1.4)	12.4 (1.2)
Female only	2.3 (0.5)	4.5 (0.7)	5.4 (0.5)	4.8 (0.9)	9.1 (1.0)	14.9 (0.7)	10.9 (1.9)	7.7 (1.2)	14.7 (1.1)
Male and female	4.2 (1.0)	5.6 (1.0)	6.5 (0.7)	7.9 (1.6)	7.6 (1.3)	18.3 (1.0)	4.9 (0.7)	10.5 (0.9)	16.5 (0.7)
Number of ill members									
One	3.0 (0.4)	4.5 (0.6)	5.2 (0.4)	7.6 (0.8)	8.5 (0.8)	15.8 (0.9)	10.1 (2.1)	7.9 (1.4)	14.3 (1.1)
Two or more	3.4 (0.7)	4.9 (0.8)	6.3 (0.6)	7.9 (1.4)	7.8 (1.1)	18.6 (1.0)	5.7 (0.6)	9.7 (0.7)	15.9 (0.7)
Comorbidity of ill members									
One disease (no comorbidity)	3.0 (0.4)	4.7 (0.5)	5.7 (0.4)	7.4 (0.7)	7.9 (0.8)	15.3 (0.8)	5.2 (0.9)	7.7 (1.0)	12.3 (0.8)
Two or more diseases	3.4 (1.0)	3.8 (1.3)	5.1 (0.7)	17.7 (7.4)	9.9 (1.5)	19.8 (1.1)	7.3 (0.9)	10.4 (0.9)	16.8 (0.7)

Numbers in parentheses are standard errors

*NCD* Noncommunicable diseases, *OOP* Out-of-pocket, *n/o* No observations

Table 5 shows the proportions of households in the different impoverishment risk categories. In 2005, OOP expenses impoverished 1.4% and 1.7% of NCD-only and NCD-and-non-NCD households, respectively, compared to 1.1% of non-NCD-only families; by 2016, the corresponding incidences reached 2.0%, and 1.5%, compared to 1.5%. Despite a decline over time, further impoverishment of families that were already below the poverty line was much higher than impoverishment of the non-poor irrespective of household NCD status (in 2005, 2010, and 2016, NCD-only: 8.0%, 8.7%, 6.7%, respectively; NCD-and-non-NCD: 7.8%, 7.3%, and 5.3%, respectively; non-NCD-only: 10.8%, 10.4%, 7.3%, respectively). However, further impoverishment was higher among households without NCDs than with NCDs. Similarly, more families without NCDs were at risk of being pushed to poverty (8.0–12.0%) than those with NCDs (6.0–10.0%) during the study period.

**Table 5:** Impoverishment effects of OOP expenditure (%), normative food, housing (rent), and utilities method

Impoverishment risk categories ^a^	Households affected by non-NCD only			Households affected by NCD only			Households affected by both NCD and non-NCD		
	2005 (*n* = 2,875)	2010 (*n* = 2,931)	2016 (*n* = 10,391)	2005 (*n* = 1,648)	2010 (*n* = 2, 449)	2016 (*n* = 9,393)	2005 (*n* = 1,806)	2010 (*n* = 2,440)	2016 (*n* = 10,160)
1. Further impoverished	10.8 (0.6)	10.4 (0.8)	7.3 (0.4)	8.0 (0.7)	8.7 (0.6)	6.7 (0.4)	7.8 (0.7)	7.3 (0.6)	5.3 (0.3)
2. Impoverished	1.1 (0.2)	1.6 (0.3)	1.5 (0.1)	1.4 (0.3)	1.4 (0.2)	2.0 (0.2)	1.7 (0.3)	1.5 (0.3)	1.5 (0.1)
3. At-risk of impoverishment ^b^	11.7 (0.6)	11.7 (0.7)	8.2 (0.4)	9.4 (0.7)	7.3 (0.5)	7.4 (0.4)	9.0 (0.7)	8.9 (0.7)	6.4 (0.3)
4. Not at-risk of impoverishment	71.3 (0.9)	68.6 (1.4)	78.3 (0.9)	78.3 (1.0)	76.3 (1.3)	79.0 (0.7)	79.3 (1.0)	77.6 (1.1)	83.4 (0.6)
5. Non-spenders	5.0 (0.4)	7.6 (1.0)	4.8 (0.4)	2.9 (0.4)	6.3 (1.0)	4.9 (0.4)	2.2 (0.4)	4.6 (0.7)	3.3 (0.3)
*Non-spenders disaggregated by reasons*									
5a. Financial reasons	0.2 (0.1)	0.2 (0.1)	0.1 (0.0)	0.1 (0.1)	0.1 (0.0)	0.1 (0.0)	0.1 (0.1)	0.1 (0.1)	0.1 (0.0)
5b. Non-financial reasons^c^	1.0 (0.2)	1.1 (0.4)	1.2 (0.2)	0.2 (0.1)	0.0 (0.0)	0.1 (0.0)	0.1 (0.1)	0.2 (0.1)	0.6 (0.1)
5c. Unspecified reasons^d^	0.0 (n/o)	0.0 (n/o)	0.0 (n/o)	1.8 (0.3)	4.2 (0.9)	3.7 (0.3)	0.0 (n/o)	0.0 (0.0)	0.1 (0.0)
5d. Non-spender but sought care	3.7 (0.4)	6.3 (0.8)	3.4 (0.3)	0.8 (0.2)	2.0 (0.5)	1.1 (0.1)	2.0 (0.4)	4.2 (0.6)	2.5 (0.3)

Numbers in parentheses are standard errors

*NCD* Noncommunicable disease, *n/o* No observations

^a^The sum of the incidences of risk categories 1, 2, 3, 4, and 5 = 100%; the sum of the incidences of the risk categories 1, 2, 3, 4, 5a, 5b, 5c, and 5d = 100%

^b^Households are at risk of impoverishment if consumption expenditure net of OOP expenses is between 100 and 120% of subsistence expenditure

^c^Non-financial reasons: health problem was not severe, distance, worried about receiving a fatal diagnosis, none to accompany, permission from the household decision-maker to seek care, didn’t know where to seek care, and others

^d^Categories 5a, 5b, and 5c represent households forgoing care. HIES provides no information on reasons for forgoing care for individuals’ health problems within the last 12 months or illnesses that occurred 30 days before the survey but were ranked second or third in order of importance. HIES collects this information only for health problems ranked the most important within 30 days before the survey

The consequences of OOP expenses on poverty were most remarkable among the poorest fifth of the population, regardless of the NCD status of the household (Additional files 3 and 4). During the study period, approximately 8.0 to 10% of NCD-affected households in the lowest quintile were impoverished, compared to 5.0 to 7.0% of non-affected households. Further impoverishment was comparable across the three household categories, declining from around 50.0% of the lowest quintile families in 2005 to 35.0% in 2016. OOP expenses pushed more rural households into poverty if they had NCDs than if they did not (e.g., 2.5% vs. 1.8% in 2016). Furthermore, similar to the distribution of CHE incidence, OOP-induced poverty, and poverty deepening were higher among NCD-affected households if all patients were female, older persons, or children, and not the principal income earner.

At the household level, the proportion of non-spenders (annual OOP = 0) were somewhat lower among families with NCDs than without NCDs (2.2–2.9% vs. 5.0% in 2005, 4.6–6.3% vs. 7.6% in 2010, and 3.3–4.9% vs. 4.8% in 2016) (Table 5). Most of these non-spending households without NCDs (e.g., 3.4% out of 4.8% in 2016) sought care (but paid zero OOP), whereas most with NCDs did not (e.g., 3.9% out of 4.9% in 2016). Only a negligible proportion of non-spending households skipped health care because they were unaffordable: a constant of 0.1% among the NCD-affected throughout the study period and 0.1–0.2% among the non-affected. Notably, the reason for forgoing care could not be ascertained for a large proportion of NCD-only families (e.g., 3.7% out of 4.9% in 2016).

At the individual level (Table 6), despite a decline over time, the proportion of people forgoing care was slightly higher among individuals with NCDs than those without NCDs (16.1–18.9% vs. 15.0% in 2005, 9.1–9.2% vs. 7.2% in 2010, and 10.6–13.6% vs. 11.9% in 2016). Not deeming the illness serious was the top reason people didn’t seek care: about a persistent 70% among individuals without NCDs and 27.0–44.0% among those with NCD-only. However, a considerably higher proportion of NCD-affected individuals forgoing care reported treatment costs as prohibitively expensive than individuals without NCDs (e.g., 24.8–37.0% vs. 13.0% in 2016). Although relatively small, distance to health facilities was also mentioned more frequently by NCD-affected individuals than those with non-NCDs only (around 4% vs. 1.4% in 2016).

**Table 6:** Reasons for forgoing care (%) for health problems within 30 days before the surveys (individual-level results)

	Individuals have non-NCDs only			Individuals have NCDs only			Individuals have both NCDs and non-NCDs		
	2005 (*n* = 5,907)	2010 (*n* = 6,986)	2016 (*n* = 23,770)	2005 (*n* = 3,632)	2010 (*n* = 5,382)	2016 (*n* = 21,385)	2005 (*n* = 926)	2010 (*n* = 1,517)	2016 (*n* = 6,922)
Any reason	15.0 (0.4)	7.2 (0.3)	11.9 (0.2)	16.1 (0.9)	9.1 (0.6)	10.6 (0.4)	18.9 (1.3)	9.2 (0.8)	13.6 (0.4)
Specific reasons (as % of all reasons)									
Problem was not severe	70.5 (1.5)	70.6 (1.9)	69.2 (0.9)	43.3 (3.1)	27.6 (3.1)	42.9 (2.2)	46.0 (3.8)	66.4 (4.1)	52.4 (1.7)
Treatment cost is too high	19.1 (1.3)	10.5 (1.4)	13.0 (0.6)	41.8 (3.0)	24.1 (2.7)	37.0 (2.1)	37.9 (3.7)	23.1 (3.6)	24.8 (1.5)
Distance is too long	1.0 (0.4)	0.9 (0.4)	1.4 (0.2)	2.3 (0.9)	1.7 (0.8)	3.6 (0.8)	2.3 (1.1)	1.5 (1.1)	3.9 (0.7)
Worried about receiving a fatal diagnosis	0.4 (0.2)	0.2 (0.2)	1.4 (0.2)	0.4 (0.4)	0.0 (n/o)	2.1 (0.6)	0.6 (0.6)	0.0 (n/o)	2.1 (0.5)
None to accompany	1.0 (0.3)	2.4 (0.7)	4.7 (0.4)	1.1 (0.7)	4.3 (1.9)	6.1 (1.0)	1.7 (1.0)	4.5 (1.8)	7.4 (0.9)
Decision maker did not permit to seek care	0.0 (n/o)	0.0 (n/o)	5.1 (0.4)	0.0 (n/o)	0.0 (n/o)	5.7 (1.0)	0.0 (n/o)	0.0 (n/o)	5.7 (0.8)
Didn’t know where to go	0.1 (0.1)	0.0 (n/o)	0.7 (0.2)	0.4 (0.4)	1.7 (0.6)	1.1 (0.4)	0.6 (0.6)	0.0 (n/o)	1.6 (0.4)
Others	8.0 (0.8)	15.5 (1.7)	4.5 (0.4)	10.6 (1.9)	39.7 (4.5)	1.3 (0.5)	10.9 (2.4)	4.5 (1.8)	2.0 (0.5)
Total	100.0	100.0	100.0	100.0	100.0	100.0	100.0	100.0	100.0

Numbers in parentheses are standard errors

*NCD* Noncommunicable diseases, *n* Number of individuals, *n/o* No observations

Our alternative computations (approach A and B) found the incidences of CHE, impoverishment effects, and households forgoing care for financial reasons to be generally higher than the results presented above (Additional files 5, 6, 7, 8, 9, 10 and 11). Nonetheless, the distribution trends and patterns of these indicators remained broadly consistent.

## Discussion

The rising NCD-related health burden and increasing share of household spending in total health expenditure warrants examining FRP among NCD-affected households over time in Bangladesh. This study is the first undertaking to investigate Bangladesh’s trajectory of FRP of households with and without NCDs from an equity perspective.

Analysis of the latest three rounds of Bangladesh HIES (2005, 2010, and 2016) data shows that OOP expenditure increased by at least 50% across all households during the research period, with NCD-affected families spending more than twice as much as unaffected households. CHE incidence increased in NCD-only families while dropping (with fluctuations) in other households. Impoverishment and further impoverishment due to OOP expenses proceeded in opposite directions, with impoverishment incidence rising among all homes and hurting those with NCDs more. Families with NCDs were worse off than those without if they were in the lowest quintile (incurred higher CHE and impoverishment), lived in rural areas, or had heads with no education (experienced higher impoverishment). The lack of FRP was more acute among NCD-affected families when the ill household members were female, elderly, or children rather than male or working-age adults. Notably, more people dealing with NCDs than non-NCDs blamed unaffordable treatment expenses for not seeking health care.

The rise in OOP spending across all households is connected to the declining government contribution to Bangladesh’s total health spending (from 22 to 18% between 2005 and 2016) [15]. Bangladesh’s sustained economic expansion has boosted the population’s purchasing power [38]. Rising incomes and education have prompted people to seek more and better care, thus increasing OOP health costs [39]. The considerably higher OOP expenses on NCDs compared to non-NCDs underscore that NCD management requires prolonged and relatively expensive treatment. Previous studies in Bangladesh also reported a significant disparity in treatment costs for chronic illnesses (including NCDs) and non-NCDs, with most of the NCD treatment expenses (56–85%) incurred for medicines [40–43]. Only 30 out of 209 drugs on the Bangladesh government’s essential drug list are for NCDs, and many are rarely prescribed [44]. Additionally, there is an overall lack of availability of medicines in the public sector, where they are dispensed free of cost, compelling patients to purchase medications from the private sector [45, 46]. The increase in the proportion of NCD-affected households (particularly among low-income families), including those with multiple ill individuals and comorbid individuals, added to the burden of OOP expenditure among affected households and, hence, resulted in the increasing trend of CHE incidence. The rising CHE trend related to NCDs contrasts with the decline in its neighbor, India, where the OOP share of current health expenditure declined (from 73 to 63%), and the government’s share increased (from 19 to 23%) over the same period [15, 47].

NCD-affected households’ OOP expenses had increasingly severe poverty effects, especially among the NCD-affected, impeding existing poverty reduction initiatives. Between 2005 and 2016, impoverishment incidence climbed from 1.1% to 1.5%, 1.4% to 2.0%, and 1.7% to 1.5% among non-NCD-only, NCD-only, and both NCD and non-NCD-affected households, respectively, or 0.4 to 0.5 million, 0.3 to 0.6 million, and 0.4 to 0.5 million people, respectively, at the population level. The current (2016) impoverishment incidence among NCD-affected households in Bangladesh (2.0%) is higher than in Nepal (1.3%) but lower than in India (5.4%), while the rate among families without NCDs (1.5%) was lower than in both countries (Nepal: 1.7%; India: 2.1%) [48, 49]. Further impoverishment was a more severe problem than impoverishment in Bangladesh, though it fell from 10.8% to 7.3%, 8.0% to 6.7%, and 7.8% to 5.3% among non-NCD-only, NCD-only, and both NCD and non-NCD-affected households, respectively, or 4.3 to 2.6 million, 1.8 to 2.2 million, and 2.0 to 1.9 million individuals, respectively. The declining trend of further impoverishment among NCD-affected families may not continue, given the study’s finding that the proportion of the lowest quintile households (that include “poor” families) with NCDs is growing over time.

The results showed a negligible rate of forgone care for financial reasons at the household level, particularly for those with NCDs (0.1% throughout the study period). The lack of information in the survey prevented determining the actual extent of forgone care for financial reasons at the household level. The survey asks about disease occurrences within 12 months and 30 days before the survey. However, the questions regarding whether individuals sought health care and the reasons if they did not only relate to the most severe illness (out of a maximum of three in order of importance) reported in the 30-day question. Therefore, it was not possible to identify the reasons for not seeking care for non-spending households that had individuals with NCDs within 12 months or 30 days before the survey but ranked the illness as second or third in order of importance for the latter. Some of these households might have had trouble affording care. Notably, a household forgoing care has zero annual OOP expenditure, i.e., the sum of annual OOP expenses of all family members is zero. When multiple household members are ill, health care for some may be prioritized over others due to financial constraints. In that situation, the family would incur OOP expenses and not be counted as forgoing care even though it skipped care for some of its members. So, the household-level incidence may not represent the actual rate of forgone care for financial reasons.

Therefore, we also looked at forgone care at the individual level, which revealed that 24.8–37.0% of people with NCDs forgoing care, or equivalently, 3.4–3.9% of all NCD-affected individuals in 2016, cited high treatment costs as the reason not to seek care. This result reinforces findings from previous studies, which consistently identified high treatment costs as a critical barrier to accessing NCD care in Bangladesh [41, 50]. In fact, despite a thriving pharmaceutical sector, common NCD drugs (those for treating hypertension, diabetes, and hypercholesterolemia) in Bangladesh were found expensive by international standards in prior research [45]. The current study also found that many individuals do not seek care because they do not deem their health problems serious (42.9% of those forgoing care or 4.5% of all NCD-only patients in 2016). A qualitative study in Bangladesh found that people with NCDs frequently wait until their condition interferes with their normal daily activities before seeking treatment; they also discontinue treatments when the disturbing symptoms subside [50]. Such results signify a widespread lack of knowledge regarding the long-term consequences of untreated NCDs.

In terms of the distribution of FRP across different equity strata, inequity was the most pronounced across consumption quintiles. The normative food, rent, and utilities approach revealed a pro-poor distribution of CHE, but the budget share estimates were pro-rich. Thus, policymaking might be misguided if the evidence is not carefully considered. The budget-share technique has been shown to exaggerate CHE among the wealthy while underestimating it among the poor [31]. The exceedingly high CHE incidence among poorer households found through the normative food, rent, and utilities method (e.g., with NCDs: 57.7%, without NCDs: 48.9% in 2016) may be partly because the approach considers any OOP expense by the poor households as catastrophic. After removing this condition in further analysis, CHE incidence was still considerably high among the poorest households, with NCD-affected families continuing to incur higher CHE than those without NCDs (e.g., with NCDs: 38.1%, without NCDs: 24.3% in 2016). Unlike some infectious diseases (such as TB) and maternal and child health problems, no free care is offered at the public health care facilities for NCDs [11]. The absence of social safety net for health care exposes low-income households to an excessive financial burden, particularly those with NCDs.

We found the impoverishment incidence of rural households with NCDs to be higher than those without NCDs. Rural households, most of whom are poor, lack access to NCD care. The first entry points into the rural public health care facilities, the Community Clinics and the Union Health and Family Welfare Centers, focus on providing non-NCD care, particularly maternal, neonatal, and child health care [51]. Although NCD corners are housed in many public primary health care facilities in the next level up (Upazila Health Complexes), they remain poorly functioning. The corners lack explicit guidelines, standard operating procedures, and shortages of other resources, including trained human resources, logistics, laboratory services, and medications [46]. As households with heads having no education predominantly belong to the poorest quintile and live in rural areas, they also bear a disproportionate financial burden.

Our study did not find intra-household resource allocation in the primary income earner’s favor among households with NCDs. Spending on health care was lower for families dealing with both NCDs and non-NCDs when the principal income earner (the household head who is an earner) was sick rather than someone else. More than 80% of these families included two or more unwell people, indicating that household heads prioritize other family members (such as elderly and children) health over their own. A previous study on NCD care-seeking behavior in Bangladesh also showed that family heads and income earners sought less care and had lower OOP expenses than non-head members and non-income earners [41]. Since earning household heads are mostly working-age adults, this group’s CHE and impoverishment incidence is lower than in other age groups across NCD-affected households.

We also found OOP expenses of NCD-affected households to be lower and CHE and impoverishment to be higher if the ailing persons were female instead of male. Gender disparity in OOP expenses may be explained by care seeking for NCDs. Previous studies in Bangladesh found men to be more likely to seek NCD care from qualified sources, whereas women mainly chose relatively cheap semi-qualified sources as their first point of contact [52, 53]. Prior studies also found that 39% of women, compared to 17% of men in resource-poor countries (including Bangladesh), do not adhere to NCD treatment [5]. However, despite lower OOP, CHE and impoverishment could be higher if the households in question are mostly low-income, for whom only a small payment for NCD care can be financially disastrous. Our further investigation confirmed this contention; 51% of NCD-only households with all female patients incurring CHE were in the poorest quintile, compared to 29% with male patients.

Two of the study results obtained through the normative food, rent, and utilities method might appear counter-intuitive. NCD-affected households with multiple ill members or comorbid individuals generally incur lower CHE, impoverishment, and further impoverishment than those with a single unwell person or without comorbid people, respectively. The above result is a methodological artifact. As mentioned above, when computing CHE incidence, the normative food, rent, and utilities technique assigns greater weights to the OOP expenses of poorer households than those of their wealthier counterparts (the effective threshold is lower for lower quintile households). Additionally, the method considers any health expenditure by "poor" households catastrophic. Our further investigation revealed that NCD-affected families with comorbid or multiple ailing members were predominantly wealthy, who are less prone to financial hardships (e.g., richest vs. poorest: 38.1% vs. 22.6% with comorbid individuals and 40.8% vs. 23.1% with two or more ill individuals in 2016). Therefore, CHE and impoverishment incidences were lower among these households (despite higher OOP expenses) than those without comorbid people or those with a single unwell person (the groups that lowest quintile households dominated). The above explanation is supported by the fact that the budget share method, which tends to overstate CHE among the wealthy and underestimate it among the poor [31], found a higher incidence of CHE among NCD-affected families with comorbid or multiple ill members than among those without such members.

Households in Bangladesh lack financial protection against OOP expenses in the event of illness, which calls for an overall health system reform. The balance must shift away from excessive dependence on OOP expenses to government funding while at the same time improving the supply-side readiness of the public delivery network and implementing demand-side programs to protect the population vulnerable to the financial risks of seeking care. A recent World Bank study found that the most effective strategy to expand fiscal space for healthcare is to give health a higher priority in the national budget [54].

Since NCD-affected households experience higher financial hardships, NCD prevention and control and reducing OOP expenses for NCDs need special attention [6, 55]. Bangladesh’s government recognizes NCDs as a major public health challenge [56, 57]. As a response, it has developed tobacco taxes, cigarette packaging, and alcohol advertising policies for NCD prevention. However, the finding that neither cigarette taxes nor health-warning images on tobacco packaging met the WHO recommendations indicates poor policy implementation [58, 59]. The latest policy, Multisectoral Action Plan for Prevention and Control of NCDs, 2018–2025, involving about 30 ministries and agencies, also suffers implementation challenges [57]. There is a gap in the participation of non-health ministries due to the lack of specific information about each ministry’s role in combating NCDs [58]. The country’s budget dedicated to NCD control (US cents 8.2/capita/year) is not commensurate with the NCD burden. It is far short of what is required to implement the "WHO best buys" in LwMICs (US$1.5/capita/year) [56, 60]. The government needs further focus and transformative measures to implement the policies successfully [56].

Increasing government investment in NCDs is crucial for lowering NCD prevalence and associated OOP expenses. Given a small tax base, the Bangladesh government will have to look for innovative sources for revenue generation [6]. Taxing tobacco, alcohol, sugary drinks, and foods rich in salt and trans-fat can improve fiscal space while lowering consumption, NCD prevalence, and health care expenditures [61]. A recent study showed that increasing the minimum price and switching to a specific excise tax of 65% on tobacco from the existing ad valorem system in Bangladesh would prevent 0.9 million premature deaths and generate 30% more than the current tax revenue [62]. Revamping the sugar-sweetened beverage tax structure, including imposing a "health development surcharge" similar to tobacco and earmarking it for endocrine problems such as diabetes, also has the potential for public health and revenue gains in Bangladesh [63]. Evidence shows that low-income consumers and young people benefit most from such levies [64]. Taxes and regulations on unhealthy products typically face strong resistance from the relevant industry [65]. Therefore, strong political will and civil society support will be imperative. Notably, Bangladesh has 181.02 million mobile cellular subscriptions (107 per 100 people), one of the world’s highest [66, 67]. Therefore, taxes on call rates could be an unorthodox but potent source of revenue for funding NCD care, although such taxes may not directly affect disease prevalence. A recent study showed that a small tax levy on mobile phone calls (USD 0.26/month) could generate funds for cancer or other chronic illness treatments in Bangladesh [68].

For NCD-affected households with lower socioeconomic status, social safety nets are critical. As part of the government’s effort to ensure FRP against OOP expenses of low-income families, it has been piloting *Shasthyo Suroksha Karmasuchi* (SSK), a tax-funded, fully subsidized social health protection scheme for the below-poverty-line (BPL) households in three sub-districts of Dhaka since 2016. Each enlisted BPL family receives a membership card, entitling members to free inpatient care for 78 disease groups, including NCDs, and outpatient consultation at the adjacent primary health care facility (Upazila Health Complex) or district hospital (in case of a referral for complicated case), with a benefit of USD 595 per household per year [69]. Supply-side readiness was improved by contracting a scheme operator, private pharmacies, diagnostic centers, and providers of support workers. According to a recent review of the scheme, enrolled families that sought care from SSK facilities had significantly lower OOP expenses and CHE incidence than BPL households in the non-SSK areas [69]. Given its effectiveness, the government can scale up the scheme to improve FRP among low-income families with and without NCDs. However, before expanding the program, implementation issues, such as misidentifying BPL households and cardholders’ lack of awareness of benefits entitlements, should be addressed to ensure the optimal outcome [69].

Additionally, since NCD care typically requires ongoing medications, the program might consider covering outpatient medicines at a subsidized price, if not for free, which would further protect low-income NCD-affected households. However, upscaling the scheme and including NCD medications in the benefits package will require substantial funding. The Philippines nearly tripled low-income family enrolment in its National Insurance Program with tobacco tax revenue from raising tobacco taxes [70]. Bangladesh may extend SSK using revenues from increased tobacco taxes like the Philippines.

The availability of NCD medications at public health facilities should be prioritized as drugs constitute the largest share of OOP expenses for NCD care [46]. A sustainable mechanism for promoting accessibility and affordability of NCD drugs should be developed for all patients. One option could be selling government-subsidized NCD medications in community pharmacies [44].

To address the rural–urban disparity in FRP, NCD services need to be effectively available in rural health facilities. NCD care might also be provided through the vast network of rural Community Clinics (13,000 in total), which could perform NCD screenings (such as hypertension and diabetes), manage uncomplicated cases, and facilitate referrals to higher-tier facilities [56]. Additionally, awareness-building campaigns promoting timely health-seeking and treatment adherence are crucial since there appears to be a general lack of knowledge regarding the long-term health and financial consequences of untreated NCDs.

The study’s primary limitation is that it relies on repeated cross-sectional data. Ideally, longitudinal or panel data should be used to determine the causative effect of financial risk associated with chronic disease in family members. Additionally, our study might be limited in that the self-reported disease prevalence in HIES is much lower than the estimates based on actual biomedical measurements from the national NCD risk factors survey (for example, 2.6% vs. 8.0% for diabetes among the adult population) [71]. Nevertheless, HIES is the most appropriate survey available in Bangladesh that has data on the necessary variables to measure FRP among households. Furthermore, due to considerable missing data on the financing source of OOP expenses, we opted not to report the incidence of distress financing, a vital indicator of a lack of FRP in health care. Despite these limitations, our study findings have significant policy implications for Bangladesh and other resource-constrained countries undergoing demographic and economic transitions and experiencing a growing burden of NCDs.

## Conclusion

Overall, all households, regardless of NCD status, lacked FRP against OOP expenses between 2005 and 2016, with the lack of FRP being more pronounced among families with NCDs than those without NCDs. Besides reprioritizing health in national budgets to reduce OOP and improve FRP for all households, transformative actions are required to protect NCD-affected families from financial hardships. Making NCD care, including drugs, effectively available at public primary health care facilities and expanding the social health protection scheme for the poorest households by adding additional NCD-related benefits are some measures to consider. These, however, would require a substantial increase in public investments. The government should consider innovative financing to strengthen its NCD response.

## Supplementary Information

**Additional file 1.**

**Additional file 2.**

**Additional file 3.**

**Additional file 4.**

**Additional file 5.**

**Additional file 6.**

**Additional file 7.**

**Additional file 8.**

**Additional file 9.**

**Additional file 10.**

**Additional file 11.**

## Data Availability

The datasets generated/or analyzed during the current study are available from Bangladesh Bureau of Statistics, Statistics and Informatics Division, Government of Bangladesh upon subscription (http://data.bbs.gov.bd/index.php/catalog/HIES/about).

## Abbreviations

BDT: Bangladeshi taka
BPL: Below-poverty-line
CHE: Catastrophic health expenditure
CPI: Consumer price index
CTP: Capacity-to-pay
DALY: Disability-adjusted life year
FRP: Financial risk protection
HIC: High-income country
HIES: Household income and expenditure survey
LIC: Low-income country
LMIC: Low- and middle-income country
LwMIC: Lower-middle-income country
NCD: Noncommunicable disease
OOP: Out-of-pocket
SDG: Sustainable development goal
SE: Subsistence expenditure
SSK: Shasthyo Suroksha Karmasuchi
USD: United States dollar
WHO: World Health Organization

## References

[1] World Health Organization (2018). Noncommunicable diseases country profiles 2018.

[2] Nishtar S, Niinistö S, Sirisena M, Vázquez T, Skvortsova V, Rubinstein A (2018). Time to deliver: report of the WHO Independent High-Level Commission on NCDs. Lancet.

[3] Bollyky TJ, Templin T, Cohen M, Dieleman JL (2017). Lower-income countries that face the most rapid shift in noncommunicable disease burden are also the least prepared. Health Aff.

[4] Global Health Estimates 2019: Disease burden by Cause, Age, Sex, by Country and by Region, 2000-2019. 2020. Available from: https://www.who.int/data/gho/data/themes/mortality-and-global-health-estimates/global-health-estimates-leading-causes-of-dalys.

[5] Murphy A, Palafox B, Walli-Attaei M, Powell-Jackson T, Rangarajan S, Alhabib KF (2020). The household economic burden of non-communicable diseases in 18 countries. BMJ Glob Health.

[6] Jan S, Laba T-L, Essue BM, Gheorghe A, Muhunthan J, Engelgau M (2018). Action to address the household economic burden of non-communicable diseases. Lancet.

[7] Kankeu HT, Saksena P, Xu K, Evans DB (2013). The financial burden from non-communicable diseases in low-and middle-income countries: a literature review. Health Res Policy Syst.

[8] Jaspers L, Colpani V, Chaker L, van der Lee SJ, Muka T, Imo D (2015). The global impact of non-communicable diseases on households and impoverishment: a systematic review. Eur J Epidemiol.

[9] World Health Organization. Fact sheet: noncommunicable diseases 2021. Available from: https://www.who.int/news-room/fact-sheets/detail/noncommunicable-diseases. updated 13 April, 2021

[10] Zhang Y, Dong D, Xu L, Miao Z, Mao W, Sloan F (2021). Ten-year impacts of China’s rural health scheme: lessons for universal health coverage. BMJ Glob Health.

[11] El-Saharty S, Ahsan KZ, Koehlmoos TLP, Engelgau MM (2013). Tackling noncommunicable diseases in Bangladesh: now is the time: World Bank Publications.

[12] Cause of death, by non-communicable diseases (% of total). 2022. Available from: https://data.worldbank.org/indicator/SH.DTH.NCOM.ZS?locations=BD-IN-PK-NP-XN-XO. [cited 17 February 2022]

[13] Alam D, Robinson H, Kanungo A, Hossain MD, Hassan M (2013). Health Systems Preparedness for responding to the growing burden of non-communicable disease-a case study of Bangladesh. Health Policy & Health Finance knowledge Hub The Nossal Institute for Global Health The University of Melbourne.

[14] Bangladesh Health Watch (2017). Non Communicable diseases in Bangladesh: current scenario and future directions. Bangladesh Health Watch Secretariat, James PGrant School of Public Health.

[15] Global Health Expenditure Database. 2022. Available from: https://apps.who.int/nha/database/ViewData/Indicators/en. [cited 18 August 2022]

[16] Rahman T, Gasbarro D, Alam K (2022). Financial risk protection in health care in Bangladesh in the era of Universal Health Coverage. PLoS ONE.

[17] Datta BK, Husain MJ, Husain MM, Kostova D (2018). Noncommunicable disease-attributable medical expenditures, household financial stress and impoverishment in Bangladesh. SSM-population health.

[18] Moreno-Serra R, Millett C, Smith PC (2011). Towards improved measurement of financial protection in health. PLoS Med.

[19] Ruger JP (2012). An alternative framework for analyzing financial protection in health. PLoS Med.

[20] Thomson S, Evetovits T, Cylus J, Jakab M (2016). Generating evidence for UHC: systematic monitoring of financial protection in European health systems. Eurohealth.

[21] World Health Organization Regional Office For Europe (2019). Can people afford to pay for health care? New evidence on financial protection in Europe.

[22] Rahman T, Gasbarro D, Alam K (2022). Financial risk protection from out-of-pocket health spending in low-and middle-income countries: a scoping review of the literature. Health Res Policy Syst.

[23] Bangladesh Bureau of Statistics (2007). Report of the Household Income and Expenditure Survey 2005. Statistics Division, Ministry of Planning: Government of Bangladesh.

[24] Bangladesh Bureau of Statistics. Report of the Household Income and Expenditure Survey 2010. Statistics Division, Ministry of Planning: Government of Bangladesh. 2011.

[25] Bangladesh Bureau of Statistics (2019). Report of the Household Income and Expenditure Survey 2016. Statistics Division, Ministry of Planning: Government of Bangladesh.

[26] Lu C, Chin B, Li G, Murray CJL. Limitations of methods for measuring out-of-pocket and catastrophic private health expenditures. Bull World Health Organ. 2009;87:238–44 10.2471/BLT.08.054379 PMC2654642 19377721

[27] Lavado RF, Brooks BPC, Hanlon M (2013). Estimating health expenditure shares from household surveys. Bull World Health Organ.

[28] Consumer price index (2010 = 100) - Bangladesh. World Bank. 2022. Available from: https://data.worldbank.org/indicator/FP.CPI.TOTL?locations=BD. [cited 25 March, 2022]

[29] Official exchange rate (LCU per US$, period average) - Bangladesh. World Bank. 2022. Available from: https://data.worldbank.org/indicator/PA.NUS.FCRF?locations=BD. [cited 01 April, 2022]

[30] Thomson S, Evetovits T, Cylus J (2018). Financial protection in high-income countries. A comparison of the Czech Republic, Estonia and Latvia Copenhagen: WHO Regional Office for Europe.

[31] Cylus J, Thomson S, Evetovits T (2018). Catastrophic health spending in Europe: equity and policy implications of different calculation methods. Bull World Health Organ.

[32] World Health Organization (2019). Can people afford to pay for health care? New evidence on financial protection in Europe.

[33] Thomson S, Evetovits T, Cylus J, Jakab M, World Health O (2016). Monitoring financial protection to assess progress towards universal health coverage in Europe. Public Health Panorama.

[34] World Health Organization (2017). Tracking universal health coverage: 2017 global monitoring report.

[35] World Health Organization (2021). Primary health care on the road to universal health coverage: 2019 global monitoring report.

[36] World Health Organization. Distribution of health payments and catastrophic expenditures methodology. 2005.

[37] Wagstaff A, Doorslaer Ev (2003). Catastrophe and impoverishment in paying for health care: with applications to Vietnam 1993–1998. Health Econ.

[38] World Bank national accounts data, GDP growth (annual %). World Bank. 2022. Available from: https://data.worldbank.org/indicator/NY.GDP.MKTP.KD.ZG?locations=BD. [cited 24.04.2022]

[39] Islam MA, Akhter S, Islam M (2018). Health financing in Bangladesh: why changes in public financial management rules will be important. Health Syst Reform.

[40] Hamid SA, Ahsan SM, Begum A (2014). Disease-specific impoverishment impact of out-of-pocket payments for health care: evidence from rural Bangladesh. Appl Health Econ Health Policy.

[41] Rasul FB, Kalmus O, Sarker M, Adib HI, Hossain MS, Hasan MZ (2019). Determinants of health seeking behavior for chronic non-communicable diseases and related out-of-pocket expenditure: results from a cross-sectional survey in northern Bangladesh. J Health Popul Nutr.

[42] Rahman MM, Zhang C, Swe KT, Rahman MS, Islam MR, Kamrujjaman M (2020). Disease-specific out-of-pocket healthcare expenditure in urban Bangladesh: A Bayesian analysis. PLoS ONE.

[43] Sarker AR, Ali SMZ, Ahmed M, Chowdhury SMZI, Ali N (2022). Out-of-pocket payment for healthcare among urban citizens in Dhaka, Bangladesh. PLoS ONE.

[44] Islam SMS, Islam MT, Islam A, Rodgers A, Chow CK, Naheed A (2017). National drug policy reform for noncommunicable diseases in low-resource countries: an example from Bangladesh. Bull World Health Organ.

[45] Kasonde L, Tordrup D, Naheed A, Zeng W, Ahmed S, Babar Z-U-D (2019). Evaluating medicine prices, availability and affordability in Bangladesh using World Health Organisation and Health Action International methodology. BMC Health Serv Res.

[46] Rawal LB, Kanda K, Biswas T, Tanim MI, Poudel P, Renzaho AMN (2019). Non-communicable disease (NCD) corners in public sector health facilities in Bangladesh: a qualitative study assessing challenges and opportunities for improving NCD services at the primary healthcare level. BMJ Open.

[47] Verma VR, Kumar P, Dash U (2021). Assessing the household economic burden of non-communicable diseases in India: evidence from repeated cross-sectional surveys. BMC Public Health.

[48] Swe KT, Rahman MM, Rahman MS, Saito E, Abe SK, Gilmour S (2018). Cost and economic burden of illness over 15 years in Nepal: A comparative analysis. PLoS ONE.

[49] Sangar S, Dutt V, Thakur R (2019). Comparative assessment of economic burden of disease in relation to out of pocket expenditure. Front Public Health.

[50] Rasul FB, Sarker M, Yasmin F, De Allegri M (2022). Exploring health-seeking behavior for non-communicable chronic conditions in northern Bangladesh. PLOS Global Public Health.

[51] World Health Organization (2017). Primary Health Care Systems (PRIMASYS): Comprehensive case study from Bangladesh.

[52] Parr JD, Lindeboom W, Khanam MA, Pérez Koehlmoos TL (2011). Diagnosis of chronic conditions with modifiable lifestyle risk factors in selected urban and rural areas of Bangladesh and sociodemographic variability therein. BMC Health Serv Res.

[53] Sikder SS, Labrique AB, Ullah B, Mehra S, Rashid M, Ali H (2012). Care-seeking patterns for fatal non-communicable diseases among women of reproductive age in rural northwest Bangladesh. BMC Women’s Health.

[54] Bonilla-Chacin ME, Hossain M, Mahmud M, Hossain S, Amin M, Sarker MAB (2020). Pathways to Reduce Household Out-of-Pocket Expenditure.

[55] Essue B, Laba T-L, Knaul F, Chu A, Minh H, Nguyen TKP (2018). Economic Burden of Chronic Ill-Health and Injuries for Households in Low-and Middle-Income Countries. Disease control priorities: improving health and reducing poverty.

[56] World Bank (2019). The continuum of care for NCDs in Bangladesh: The time is to act now!.

[57] Government of Bangladesh (2018). Multi-sectoral action plan for prevention and control of non-communicable diseases 2018–2025.

[58] Elfarra RM (2021). A stakeholder analysis of noncommunicable diseases’ multisectoral action plan in Bangladesh. WHO South-East Asia J Public Health.

[59] Siddiqi K, Scammell K, Huque R, Khan A, Baral S, Ali S (2015). Smokeless tobacco supply chain in South Asia: a comparative analysis using the who framework convention on tobacco control. Nicotine Tob Res.

[60] World Health Organization. Saving lives, spending less: a strategic response to noncommunicable diseases. 2018.

[61] NCD Alliance. Policy Brief: Invest to Protect. NCD financing as the foundation for healthy societies and economies 2022. Available from: https://ncdalliance.org/resources/invest-to-protect-ncd-financing-as-the-foundation-for-healthy-societies-and-economies.

[62] Institute for Health Research and Policy. Raising Tobacco Taxes in FY 2022-2023: A crucial step towards a tobacco-free Bangladesh by 2040 [Internet]. Chicago (USA): University of Illinois; 2022 [Available from: https://tobacconomics.org/files/research/753/bangladesh-tax-factsheet-2022-2023-jan-21-2022.pdf.

[63] ARK Foundation. Policy Brief. Taxation on Sugar-Sweetened Beverages (SSBs) in Bangladesh: What should we do? 2020. Available from: https://arkfoundationbd.org/wp-content/uploads/2020/12/Policy-brief-on-SSB_13.12.2020-brief-1.pdf.

[64] Colchero MA, Rivera-Dommarco J, Popkin BM, Ng SW (2017). In Mexico, evidence of sustained consumer response two years after implementing a sugar-sweetened beverage tax. Health Aff.

[65] Countdown NCD (2022). NCD Countdown 2030: efficient pathways and strategic investments to accelerate progress towards the Sustainable Development Goal target 3.4 in low-income and middle-income countries. Lancet..

[66] Mobile cellular subscriptions (per 100 people) - Bangladesh. 2022. Available from: https://data.worldbank.org/indicator/IT.CEL.SETS.P2. [cited 07.09.2022]

[67] Bangladesh Telecommunication Regulatory Commission. Total number of mobile phone subscribers 2022. Available from: http://old.btrc.gov.bd/content/mobile-phone-subscribers-bangladesh-december-2021. [cited 07.09.2022]

[68] Hamid SA, Talukder MH, Tasnim A, Ihsan-Ul-Kabir M (2021). Financing cancer Care in Bangladesh: an alternative route. Int J Soc Adm Sci.

[69] Chowdhury ME, Hasan MZ, Akter R, Mehdi GG, Ahmed MW, Chowdhury A (2021). Evaluation of the Pilot Shasthyo Shurokhsha Karmasuchi (SSK).

[70] Goodchild M, Perucic AM, Zheng R, Blecher E, Paul J (2017). The health impact of raising tobacco taxes in developing countries. Tobacco tax reform–at the crossroads of health and development Washington DC, World Bank Group.

[71] World Health Organization (2018). National STEPS survey for non-communicable diseases risk factors in Bangladesh 2018.

