# Supplementary material for: Financial risk protection against noncommunicable diseases: trends and patterns in Bangladesh
Source: BMC Public Health. 2022 Sep 30;22:1835. doi: 10.1186/s12889-022-14243-0 (PMC9524135; doi:10.1186/s12889-022-14243-0)
Supplement: Supplementary file 1 — Additional file 1. [file 12889_2022_14243_MOESM1_ESM.docx]

**Additional file 1:** Alternative calculations used for measuring financial risk protection (FRP) indicators to account for the discrepancy in OOP expenses between the HIES health and consumption modules

Annual out-of-pocket (OOP) expenditure differs substantially between the health (*oop_h_*) and consumption modules (*oop_c_*) in each HIES round, with *oop_c <_ oop_h_* . This discrepancy might lead to different financial risk protection (FRP) statuses for a given household and, consequently, different levels and distributions of FRP indicators across all households in the same year, depending on the source of OOP spending data utilized in the analysis. To verify, we produced three sets of results, one in the main text and the other two in additional files. In what follows are the details of these three alternative measurement techniques.

Estimation of FRP indicators through the normative food, housing (rent), and utilities method is based on the ratio of two key variables: household's OOP expenditure and capacity-to-pay for health care, $ctp$. The latter is defined as what is left of household’s total income, in this case approximated by household’s total consumption expenditure ($c$), after the household meets it’s subsistence needs ($se$); i.e., $ctp= c-se$. So, $ctp$ depends on $c$. Again, $c$ can be expressed as the sum of food expenditure $(f)$, health care expenditure $(oop)$, and all other expenditures $(other)$. Therefore,

$$\frac{oop}{ctp}= \frac{oop}{c-se} = \frac{oop}{(f+oop+other)-se}$$

Consequently, OOP is there both in the numerator and the denominator of $\frac{oop}{ctp}$.

**Approach used in the main text:**  Both the numerator and denominator OOP were extracted from the consumption module (${oop}_{c})$; i.e.,

$\frac{oop}{ctp}= \frac{oop}{c-se} = \frac{{oop}_{c}}{(f+{oop}_{c}+other)-se}$

This approach produces the most conservative estimates of the incidences of FRP indicators among the three.

**Alternative measurement approach A (results showed in additional files) :** Both the numerator and denominator OOP were from the health module (${oop}_{h}$); i.e.,

$\frac{oop}{ctp}= \frac{oop}{c-se} = \frac{{oop}_{h}}{(f+{oop}_{h}+other)-se}$

**Alternative measurement approach B (results showed in additional files):** We extracted OOP expenditure from the health module and total consumption expenditure from the consumption module (in line with the survey modules’ purpose). Therefore, the numerator OOP was from the health module (${oop}_{h}$) but the denominator OOP came from the consumption module (${oop}_{c})$; i.e.,

$\frac{oop}{ctp}= \frac{oop}{c-se} = \frac{{oop}_{h}}{(f+{oop}_{c}+other)-se}$

Since *oop_h_ > oop_c ,_* alternative measurement approach B produces the largest estimates of the incidences of FRP estimates.

Our all-inclusive consideration of sources of information on OOP payments combined with exhaustive ways to analyze them yielded an upper and a lower limit of the incidences of financial protection indicators for each round of HIES.
