# Supplementary material for: Financial risk protection against noncommunicable diseases: trends and patterns in Bangladesh
Source: BMC Public Health. 2022 Sep 30;22:1835. doi: 10.1186/s12889-022-14243-0 (PMC9524135; doi:10.1186/s12889-022-14243-0)
Supplement: Supplementary file 2 — Additional file 2. [file 12889_2022_14243_MOESM2_ESM.docx]

**Additional file 2:** Example questions regarding disease occurrences and health care-seeking behavior in Bangladesh Household Income and Expenditure Survey’s (HIES) health module and definition of the categories of non-spending households

1. **Example questions regarding an individual’s health status and care-seeking behavior in the HIES health module**

- Have you suffered from any chronic illness/ disability in the last 12 months?
- What chronic illness/ disability are you suffering from? List two in order of importance.
- Have you suffered from any symptoms of illness/ injury in the last 30 days?
- If yes, what symptoms/ diseases did you suffer from? List three in order of importance
- Have you sought any medical treatment related to the most important health problem you suffered in the last 30 days?
- If no, why did you not seek any treatment?

1. **Definition of the categories of non-spending households**

Non-spending households have zero OOP expenses. We categorized these households based on whether or not they sought medical attention, further partitioning those forgoing care by reasons for doing so (financial, non-financial, unspecified).

- *Non-spender, financial reasons*: Households with individuals suffering from diseases (NCDs, non-NCDs, or both NCDs and non-NCDs) within the last 30 days preceding the survey that reported zero OOP expenses because health care was not sought. Additionally, all the individuals in the household forgoing care did so because of financial reasons (high/ unaffordable costs) implying the number of individuals forgoing care within a household equaled the number of individuals mentioning financial barrier as the reason.
- *Non-spender, non-financial reasons*: Non-spending households that had at least one individual in the household who did not seek care for their health problems that occurred within the last 30 days for non-financial reasons. The reasons are stated as “health problem was not serous”, “distance is too long”, “afraid of discovering serious illness”, “none to accompany”, “decision maker does not think I should seek treatment”, “don’t know where to go”, and “others”.
- *Non-spender, unspecified reasons:* These households had individuals with NCDs within the last 12 months or had NCDs or non-NCDs within 30 days before the survey (which they ranked second or third in order of importance) but reported no health expenditure. No information was available to confirm if these households experienced financial or other barriers or if they sought care but did not have to pay.
- *Non-spenders but sought health care:* Households that had individuals suffering from disease(s)/ symptom(s) within the last 30 days, sought health care but had zero OOP.
