# Supplementary material for: Financial risk protection against noncommunicable diseases: trends and patterns in Bangladesh
Source: BMC Public Health. 2022 Sep 30;22:1835. doi: 10.1186/s12889-022-14243-0 (PMC9524135; doi:10.1186/s12889-022-14243-0)
Supplement: Supplementary file 4 — Additional file 4. [file 12889_2022_14243_MOESM4_ESM.docx]

**Additional file 4:** Incidence of further impoverishment ^a^ due to OOP expenses (%), normative food, housing (rent), and utilities method

|  | Households affected by non-NCD only | | | Households affected by NCD only | | | Households affected by both NCD & non-NCD | | |
| --- | --- | --- | --- | --- | --- | --- | --- | --- | --- |
|  | 2005  (n = 2,875) | 2010  (n = 2,931) | 2016  (n = 10,391) | 2005  (n = 1,648) | 2010  (n = 2, 449) | 2016  (n = 9,393) | 2005  (n=1,806) | 2010  (n = 2,440) | 2016  (n = 10,160) |
|  |  |  |  |  |  |  |  |  |  |
| Overall | 10.8  (0.6) | 10.4  (0.8) | 7.3  (0.4) | 8.0  (0.7) | 8.7  (0.6) | 6.7  (0.4) | 7.8  (0.7) | 7.3  (0.6) | 5.3  (0.3) |
|  |  |  |  |  |  |  |  |  |  |
| Consumption expenditure quintile |  |  |  |  |  |  |  |  |  |
| Lowest^b^ | 50.7  (2.2) | 45.1  (2.3) | 33.9  (1.3) | 49.6  (3.2) | 51.2  (2.4) | 35.0  (1.3) | 49.0  (3.1) | 44.9  (2.6) | 34.9  (1.6) |
|  |  |  |  |  |  |  |  |  |  |
| Area of residence |  |  |  |  |  |  |  |  |  |
| Rural | 12.6  (0.7) | 12.0  (1.0) | 9.2  (0.6) | 10.0  (0.9) | 11.7  (0.8) | 8.2  (0.5) | 9.2  (0.8) | 8.1  (0.7) | 6.5  (0.4) |
|  |  |  |  |  |  |  |  |  |  |
| Urban | 4.6  (0.5) | 4.5  (0.7) | 2.9  (0.5) | 2.8  (0.5) | 2.0  (0.3) | 2.3  (0.3) | 3.3  (0.6) | 3.9  (0.8) | 1.9  (0.4) |
|  |  |  |  |  |  |  |  |  |  |
| Household head's education |  |  |  |  |  |  |  |  |  |
| No education | 15.9  (0.9) | 14.7  (1.2) | 11.1  (0.7) | 12.7  (1.2) | 14.4  (1.0) | 11.1  (0.6) | 11.9  (1.1) | 11.3  (0.9) | 8.4  (0.6) |
|  |  |  |  |  |  |  |  |  |  |
| Below secondary | 5.5  (0.8) | 7.1  (0.9) | 5.7  (0.6) | 4.1  (0.9) | 4.5  (0.8) | 4.2  (0.4) | 4.1  (0.9) | 3.3  (0.7) | 3.9  (0.3) |
|  |  |  |  |  |  |  |  |  |  |
| Secondary or above | 0.9  (0.5) | 1.8  (0.7) | 1.2  (0.3) | 0.8  (0.6) | 0.1  (0.1) | 1.0  (0.3) | 0.5  (0.5) | 1.3  (0.6) | 1.0  (0.3) |
|  |  |  |  |  |  |  |  |  |  |
| Illness of main income earner |  |  |  |  |  |  |  |  |  |
| No | 9.9  (0.7) | 10.3  (0.9) | 6.9  (0.5) | 8.1  (0.9) | 9.3  (0.8) | 7.3  (0.5) | 7.9  (1.0) | 7.6  (0.9) | 5.3  (0.5) |
|  |  |  |  |  |  |  |  |  |  |
| Yes | 13.9  (1.4) | 10.7  (1.3) | 8.4  (0.7) | 8.0  (1.0) | 7.9  (0.9) | 5.9  (0.5) | 7.7  (0.9) | 7.1  (0.8) | 5.3  (0.4) |
|  |  |  |  |  |  |  |  |  |  |
| Age composition of ill members |  |  |  |  |  |  |  |  |  |
| Children (<18 years) only | 10.5  (0.9) | 12.0  (1.3) | 6.5  (0.6) | 11.1  (3.5) | 13.4  (3.2) | 9.7  (1.9) | 8.1  (3.4) | 9.3  (4.6) | 7.8  (1.6) |
|  |  |  |  |  |  |  |  |  |  |
| Non-elderly adults (18-60 years) only | 10.5  (1.1) | 9.4  (1.0) | 7.3  (0.6) | 6.9  (0.8) | 7.2  (0.7) | 5.4  (0.4) | 7.2  (1.2) | 7.8  (1.1) | 5.6  (0.7) |
|  |  |  |  |  |  |  |  |  |  |
| Elderly (>60 years) only | 19.2  (3.3) | 18.8  (4.0) | 18.5  (2.2) | 12.2  (2.1) | 14.2  (1.8) | 12.2  (1.0) | 16.4  (4.5) | 19.8  (3.5) | 16.1  (1.9) |
|  |  |  |  |  |  |  |  |  |  |
| Children and non-elderly adults | 10.0  (1.6) | 7.5  (1.3) | 6.2  (0.7) | 11.2  (5.3) | 3.3  (2.1) | 3.5  (1.1) | 7.7  (1.0) | 6.0  (0.8) | 4.2  (0.4) |
|  |  |  |  |  |  |  |  |  |  |
| Non-elderly adults and elderly | 1.8  (1.8) | 11.6  (5.5) | 8.7  (3.0) | 5.3  (2.2) | 6.9  (2.1) | 3.3  (0.6) | 8.9  (2.3) | 5.0  (1.4) | 4.4  (0.7) |
|  |  |  |  |  |  |  |  |  |  |
| Children and elderly | 12.9  (0.7) | 0.0  (n/o) | 12.6  (5.2) | 0.0  (n/o) | 18.7  (13.1) | 0.0  (n/o) | 6.5  (0.7) | 6.6  (2.5) | 2.9  (0.9) |
|  |  |  |  |  |  |  |  |  |  |
| Gender composition of ill members |  |  |  |  |  |  |  |  |  |
| Male only | 10.9  (1.0) | 11.1  (1.2) | 7.0  (0.6) | 7.4  (1.1) | 6.9  (1.0) | 6.7  (0.6) | 8.4  (1.7) | 7.6  (1.6) | 6.1  (0.8) |
|  |  |  |  |  |  |  |  |  |  |
| Female only | 11.2  (1.0) | 11.2  (1.2) | 8.0  (0.6) | 9.5  (1.1) | 11.7  (1.0) | 8.6  (0.6) | 13.1  (1.9) | 11.8  (1.6) | 9.4  (0.9) |
|  |  |  |  |  |  |  |  |  |  |
| Male and female | 9.8  (1.4) | 7.5  (1.1) | 6.3  (0.7) | 5.8  (1.4) | 5.5  (1.1) | 3.4  (0.4) | 6.1  (0.7) | 5.8  (0.6) | 3.8  (0.3) |
|  |  |  |  |  |  |  |  |  |  |
| Number of ill members |  |  |  |  |  |  |  |  |  |
| One | 11.1  (0.7) | 11.2  (1.0) | 7.2  (0.5) | 8.4  (0.8) | 10.0  (0.7) | 8.1  (0.5) | 12.8  (2.1) | 15.0  (2.0) | 11.4  (1.2) |
|  |  |  |  |  |  |  |  |  |  |
| Two or more | 10.1  (1.1) | 8.7  (1.0) | 8.0  (0.9) | 6.6  (1.3) | 5.1  (0.9) | 3.4  (0.4) | 6.9  (0.7) | 5.9  (0.6) | 4.0  (0.3) |
| Comorbidity of ill members |  |  |  |  |  |  |  |  |  |
| One disease (no comorbidity) | 10.3  (0.6) | 10.6  (0.8) | 7.2  (0.5) | 8.2  (0.7) | 9.1  (0.7) | 7.5  (0.4) | 6.5  (0.9) | 5.9  (0.9) | 4.6  (0.5) |
|  |  |  |  |  |  |  |  |  |  |
| Two or more diseases | 13.7  (1.7) | 9.2  (2.0) | 7.7  (0.8) | 0.0  (n/o) | 7.0  (1.2) | 4.7  (0.5) | 8.8  (1.0) | 8.1  (0.8) | 5.6  (0.4) |

NCD = noncommunicable diseases, OOP = out-of-pocket, n/o = no observations

Numbers in parentheses are standard errors

^a^ Any health expenditure by a poor household is considered further impoverishing. A household is poor when its total consumption expenditure is less than subsistence expenditure. Subsistence expenditure is defined as the average per (equivalent) person spending on food, rent, and utilities of the households between the 25th and 35th percentiles of the per (equivalent) person total consumption expenditure distribution.

^b^ The poorest consumption quintile consists of poor and non-poor households. No household among the second and higher quintiles was poor. Hence, the further impoverishment incidence is not available for the upper four quintiles.
