# Supplementary material for: Financial risk protection against noncommunicable diseases: trends and patterns in Bangladesh
Source: BMC Public Health. 2022 Sep 30;22:1835. doi: 10.1186/s12889-022-14243-0 (PMC9524135; doi:10.1186/s12889-022-14243-0)
Supplement: Supplementary file 5 — Additional file 5. [file 12889_2022_14243_MOESM5_ESM.docx]

**Additional file 5:** Alternative calculation of background characteristics of households affected by NCD only, non-NCD only and both NCD and non-NCD

**Alternative measurement approach A** [using OOP expenses (as a separate variable and as a component of total consumption expenditure) from the survey’s health module]

|  | Households affected by non-NCD only | | | Households affected by NCD only | | | Households affected by both NCD & non-NCD | | |
| --- | --- | --- | --- | --- | --- | --- | --- | --- | --- |
|  | 2005  (n = 2,875) | 2010  (n = 2,931) | 2016  (n = 10,391) | 2005  (n = 1,648) | 2010  (n = 2, 449) | 2016  (n = 9,393) | 2005  (n=1,806) | 2010  (n = 2,440) | 2016  (n = 10,160) |
|  |  |  |  |  |  |  |  |  |  |
| Overall | 28.5  (0.5) | 24.0  (0.6) | 22.6  (0.5) | 16.4  (0.4) | 20.0  (0.5) | 20.4  (0.4) | 17.9  (0.4) | 19.9  (0.7) | 22.1  (0.4) |
|  |  |  |  |  |  |  |  |  |  |
| Consumption expenditure quintile |  |  |  |  |  |  |  |  |  |
| Lowest | 19.3  (0.7) | 21.6  (1.0) | 20.7  (0.9) | 17.2  (0.9) | 17.8  (0.9) | 18.2  (0.7) | 15.2  (0.9) | 15.1  (0.9) | 13.6  (0.6) |
|  |  |  |  |  |  |  |  |  |  |
| 2nd | 22.3  (0.8) | 22.1  (0.9) | 21.4  (0.8) | 18.4  (1.0) | 16.8  (0.8) | 18.7  (0.6) | 16.7  (0.9) | 18.4  (0.9) | 17.5  (0.6) |
|  |  |  |  |  |  |  |  |  |  |
| 3rd | 21.2  (0.8) | 20.4  (0.8) | 20.9  (0.7) | 18.3  (1.0) | 18.3  (0.9) | 19.3  (0.6) | 20.4  (1.0) | 20.5  (0.9) | 19.6  (0.7) |
|  |  |  |  |  |  |  |  |  |  |
| 4th | 19.5  (0.8) | 19.9  (0.9) | 19.6  (1.0) | 20.1  (1.1) | 20.9  (1.0) | 19.8  (0.6) | 22.7  (1.1) | 22.3  (1.0) | 22.4  (0.6) |
|  |  |  |  |  |  |  |  |  |  |
| Highest | 17.7  (0.8) | 16.0  (1.0) | 17.4  (0.9) | 26.0  (1.1) | 26.2  (1.3) | 24.0  (0.9) | 25.0  (1.1) | 23.6  (1.2) | 26.9  (1.1) |
|  |  |  |  |  |  |  |  |  |  |
| Area of residence |  |  |  |  |  |  |  |  |  |
| Rural | 78.0  (0.0) | 78.9  (1.0) | 70.0  (1.7) | 72.1  (0.0) | 69.0  (1.0) | 74.2  (1.1) | 76.2  (0.0) | 81.3  (1.0) | 75.6  (1.1) |
|  |  |  |  |  |  |  |  |  |  |
| Urban | 22.0  (0.0) | 21.1  (1.0) | 30.0  (1.7) | 27.9  (0.0) | 31.0  (1.0) | 25.8  (1.1) | 23.8  (0.0) | 18.7  (1.0) | 24.4  (1.1) |
|  |  |  |  |  |  |  |  |  |  |
| Household head's education |  |  |  |  |  |  |  |  |  |
| No education | 57.3  (1.0) | 53.5  (1.2) | 40.4  (0.9) | 52.1  (1.3) | 50.9  (1.3) | 43.8  (0.8) | 54.1  (1.2) | 54.0  (1.2) | 42.0  (0.9) |
|  |  |  |  |  |  |  |  |  |  |
| Below secondary | 29.8  (0.9) | 33.2  (1.1) | 46.0  (0.8) | 30.5  (1.2) | 29.5  (1.1) | 39.6  (0.7) | 31.1  (1.2) | 31.9  (1.1) | 43.5  (0.8) |
|  |  |  |  |  |  |  |  |  |  |
| Secondary or above | 12.9  (0.7) | 13.4  (0.8) | 13.6  (0.8) | 17.4  (1.0) | 19.6  (1.3) | 16.6  (0.8) | 14.7  (0.9) | 14.1  (0.8) | 14.5  (0.8) |
|  |  |  |  |  |  |  |  |  |  |
| Illness of main income earner |  |  |  |  |  |  |  |  |  |
| No | 76.0  (0.9) | 72.9  (1.0) | 74.6  (0.7) | 56.6  (1.3) | 57.7  (1.2) | 58.4  (0.8) | 42.9  (1.2) | 44.8  (1.1) | 46.6  (0.8) |
|  |  |  |  |  |  |  |  |  |  |
| Yes | 24.0  (0.9) | 27.1  (1.0) | 25.4  (0.7) | 43.4  (1.3) | 42.3  (1.2) | 41.6  (0.8) | 57.1  (1.2) | 55.2  (1.1) | 53.4  (0.8) |
|  |  |  |  |  |  |  |  |  |  |
| Age composition of ill members |  |  |  |  |  |  |  |  |  |
| Children (<18 years) only | 44.9  (1.0) | 38.5  (1.1) | 39.6  (0.7) | 5.5  (0.6) | 4.9  (0.5) | 4.2  (0.4) | 4.0  (0.5) | 2.1  (0.3) | 4.5  (0.5) |
|  |  |  |  |  |  |  |  |  |  |
| Non-elderly adults (18-60 years) only | 32.6  (1.0) | 38.3  (1.0) | 35.5  (0.8) | 68.3  (1.2) | 65.5  (1.1) | 63.3  (0.7) | 29.3  (1.2) | 29.6  (1.1) | 31.3  (0.7) |
|  |  |  |  |  |  |  |  |  |  |
| Elderly (>60 years) only | 6.0  (0.5) | 4.6  (0.4) | 4.1  (0.3) | 16.9  (1.0) | 19.2  (1.0) | 20.1  (0.7) | 4.6  (0.6) | 7.3  (0.6) | 6.8  (0.4) |
|  |  |  |  |  |  |  |  |  |  |
| Children and non-elderly adults | 14.5  (0.7) | 16.7  (0.8) | 19.0  (0.6) | 2.8  (0.5) | 3.3  (0.4) | 3.1  (0.2) | 45.3  (1.3) | 44.5  (1.2) | 42.5  (0.8) |
|  |  |  |  |  |  |  |  |  |  |
| Non-elderly adults and elderly | 0.9  (0.2) | 1.3  (0.2) | 1.0  (0.1) | 6.4  (0.6) | 6.8  (0.6) | 8.9  (0.4) | 10.8  (0.8) | 11.9  (0.8) | 10.3  (0.4) |
|  |  |  |  |  |  |  |  |  |  |
| Children and elderly | 1.0  (0.2) | 0.6  (0.1) | 0.8  (0.1) | 0.0  (0.0) | 0.3  (0.1) | 0.3  (0.1) | 6.0  (0.6) | 4.5  (0.5) | 4.6  (0.3) |
|  |  |  |  |  |  |  |  |  |  |
| Gender composition of ill members |  |  |  |  |  |  |  |  |  |
| Male only | 40.0  (1.0) | 38.5  (1.1) | 34.1  (0.8) | 37.3  (1.3) | 32.9  (1.1) | 29.6  (0.6) | 16.5  (0.9) | 12.8  (0.8) | 12.0  (0.5) |
|  |  |  |  |  |  |  |  |  |  |
| Female only | 42.4  (1.0) | 41.2  (1.1) | 43.2  (0.8) | 43.6  (1.3) | 43.7  (1.2) | 44.0  (0.7) | 19.1  (1.0) | 21.3  (0.9) | 22.2  (0.6) |
|  |  |  |  |  |  |  |  |  |  |
| Male and female | 17.6  (0.8) | 20.3  (0.9) | 22.6  (0.7) | 19.1  (1.0) | 23.3  (1.1) | 26.5  (0.7) | 64.4  (1.2) | 65.9  (1.1) | 65.8  (0.8) |
|  |  |  |  |  |  |  |  |  |  |
| Number of ill members |  |  |  |  |  |  |  |  |  |
| One | 71.4  (0.9) | 68.6  (1.0) | 66.3  (0.8) | 77.3  (1.1) | 73.3  (1.2) | 70.2  (0.7) | 15.1  (0.9) | 15.6  (0.8) | 17.8  (0.7) |
|  |  |  |  |  |  |  |  |  |  |
| Two or more | 28.6  (0.9) | 31.4  (1.0) | 33.7  (0.8) | 22.7  (1.1) | 26.7  (1.2) | 29.8  (0.7) | 84.9  (0.9) | 84.4  (0.8) | 82.2  (0.7) |
|  |  |  |  |  |  |  |  |  |  |
| Comorbidity of ill members |  |  |  |  |  |  |  |  |  |
| One disease (no comorbidity) | 83.4  (0.7) | 90.7  (0.8) | 76.8  (1.2) | 97.6  (0.4) | 80.2  (1.0) | 70.3  (0.8) | 45.2  (1.3) | 36.5  (1.3) | 26.5  (0.7) |
|  |  |  |  |  |  |  |  |  |  |
| Two or more diseases | 16.6  (0.7) | 9.3  (0.8) | 23.2  (1.2) | 2.4  (0.4) | 19.8  (1.0) | 29.7  (0.8) | 54.8  (1.3) | 63.5  (1.3) | 73.5  (0.7) |

NCD = noncommunicable diseases, OOP = out-of-pocket, CTP = capacity to-pay, Numbers in parentheses are standard errors

Total number of households included in analysis: 10,075 in 2005, 12,237 in 2010, and 45,976 in 2016

**Alternative measurement approach B** [using OOP expenses (as a separate variable) from the survey’s health module, and the OOP component of total consumption expenditure (thus CTP) from the consumption module]

|  | Households affected by non-NCD only | | | Households affected by NCD only | | | Households affected by both NCD & non-NCD | | |
| --- | --- | --- | --- | --- | --- | --- | --- | --- | --- |
|  | 2005  (n = 2,875) | 2010  (n = 2,931) | 2016  (n = 10,391) | 2005  (n = 1,648) | 2010  (n = 2, 449) | 2016  (n = 9,393) | 2005  (n=1,806) | 2010  (n = 2,440) | 2016  (n = 10,160) |
|  |  |  |  |  |  |  |  |  |  |
| Overall | 28.5  (0.5) | 24.0  (0.6) | 22.6  (0.5) | 16.4  (0.4) | 20.0  (0.5) | 20.4  (0.4) | 17.9  (0.4) | 19.9  (0.7) | 22.1  (0.4) |
|  |  |  |  |  |  |  |  |  |  |
| Consumption expenditure quintile |  |  |  |  |  |  |  |  |  |
| Lowest | 21.3  (0.8) | 23.1  (1.1) | 21.5  (0.9) | 16.2  (0.9) | 17.0  (0.9) | 19.1  (0.7) | 15.9  (0.9) | 16.3  (1.0) | 15.3  (0.6) |
|  |  |  |  |  |  |  |  |  |  |
| 2nd | 22.6  (0.8) | 22.7  (0.9) | 21.2  (0.8) | 17.9  (1.0) | 16.5  (0.8) | 19.2  (0.6) | 17.3  (0.9) | 19.0  (1.0) | 18.6  (0.6) |
|  |  |  |  |  |  |  |  |  |  |
| 3rd | 20.8  (0.8) | 19.9  (0.8) | 20.1  (0.7) | 18.9  (1.0) | 19.0  (0.9) | 19.9  (0.6) | 20.4  (1.0) | 20.7  (0.9) | 19.4  (0.6) |
|  |  |  |  |  |  |  |  |  |  |
| 4th | 18.6  (0.8) | 19.0  (0.9) | 19.9  (1.0) | 20.6  (1.1) | 21.3  (1.0) | 19.1  (0.7) | 21.3  (1.1) | 21.6  (1.0) | 21.3  (0.6) |
|  |  |  |  |  |  |  |  |  |  |
| Highest | 16.6  (0.7) | 15.4  (1.0) | 17.5  (1.0) | 26.4  (1.1) | 26.3  (1.3) | 22.7  (0.8) | 25.1  (1.0) | 22.4  (1.1) | 25.4  (1.2) |
|  |  |  |  |  |  |  |  |  |  |
| Area of residence |  |  |  |  |  |  |  |  |  |
| Rural | 78.0  (0.0) | 78.9  (1.0) | 70.0  (1.7) | 72.1  (0.0) | 69.0  (1.0) | 74.2  (1.1) | 76.2  (0.0) | 81.3  (1.0) | 75.6  (1.1) |
|  |  |  |  |  |  |  |  |  |  |
| Urban | 22.0  (0.0) | 21.1  (1.0) | 30.0  (1.7) | 27.9  (0.0) | 31.0  (1.0) | 25.8  (1.1) | 23.8  (0.0) | 18.7  (1.0) | 24.4  (1.1) |
|  |  |  |  |  |  |  |  |  |  |
| Household head's education |  |  |  |  |  |  |  |  |  |
| No education | 57.3  (1.0) | 53.5  (1.2) | 40.4  (0.9) | 52.1  (1.3) | 50.9  (1.3) | 43.8  (0.8) | 54.1  (1.2) | 54.0  (1.2) | 42.0  (0.9) |
|  |  |  |  |  |  |  |  |  |  |
| Below secondary | 29.8  (0.9) | 33.2  (1.1) | 46.0  (0.8) | 30.5  (1.2) | 29.5  (1.1) | 39.6  (0.7) | 31.1  (1.2) | 31.9  (1.1) | 43.5  (0.8) |
|  |  |  |  |  |  |  |  |  |  |
| Secondary or above | 12.9  (0.7) | 13.4  (0.8) | 13.6  (0.8) | 17.4  (1.0) | 19.6  (1.3) | 16.6  (0.8) | 14.7  (0.9) | 14.1  (0.8) | 14.5  (0.8) |
|  |  |  |  |  |  |  |  |  |  |
| Illness of main income earner |  |  |  |  |  |  |  |  |  |
| No | 76.0  (0.9) | 72.9  (1.0) | 74.6  (0.7) | 56.6  (1.3) | 57.7  (1.2) | 58.4  (0.8) | 42.9  (1.2) | 44.8  (1.1) | 46.6  (0.8) |
|  |  |  |  |  |  |  |  |  |  |
| Yes | 24.0  (0.9) | 27.1  (1.0) | 25.4  (0.7) | 43.4  (1.3) | 42.3  (1.2) | 41.6  (0.8) | 57.1  (1.2) | 55.2  (1.1) | 53.4  (0.8) |
|  |  |  |  |  |  |  |  |  |  |
| Age composition of ill members |  |  |  |  |  |  |  |  |  |
| Children (<18 years) only | 44.9  (1.0) | 38.5  (1.1) | 39.6  (0.7) | 5.5  (0.6) | 4.9  (0.5) | 4.2  (0.4) | 4.0  (0.5) | 2.1  (0.3) | 4.5  (0.5) |
| Non-elderly adults (18-60 years) only |  |  |  |  |  |  |  |  |  |
| Elderly (>60 years) only | 32.6  (1.0) | 38.3  (1.0) | 35.5  (0.8) | 68.3  (1.2) | 65.5  (1.1) | 63.3  (0.7) | 29.3  (1.2) | 29.6  (1.1) | 31.3  (0.7) |
| Children (<18 years) only |  |  |  |  |  |  |  |  |  |
| Non-elderly adults (18-60 years) only | 6.0  (0.5) | 4.6  (0.4) | 4.1  (0.3) | 16.9  (1.0) | 19.2  (1.0) | 20.1  (0.7) | 4.6  (0.6) | 7.3  (0.6) | 6.8  (0.4) |
|  |  |  |  |  |  |  |  |  |  |
| Children and non-elderly adults | 14.5  (0.7) | 16.7  (0.8) | 19.0  (0.6) | 2.8  (0.5) | 3.3  (0.4) | 3.1  (0.2) | 45.3  (1.3) | 44.5  (1.2) | 42.5  (0.8) |
|  |  |  |  |  |  |  |  |  |  |
| Non-elderly adults and elderly | 0.9  (0.2) | 1.3  (0.2) | 1.0  (0.1) | 6.4  (0.6) | 6.8  (0.6) | 8.9  (0.4) | 10.8  (0.8) | 11.9  (0.8) | 10.3  (0.4) |
|  |  |  |  |  |  |  |  |  |  |
| Children and elderly | 1.0  (0.2) | 0.6  (0.1) | 0.8  (0.1) | 0.0  (0.0) | 0.3  (0.1) | 0.3  (0.1) | 6.0  (0.6) | 4.5  (0.5) | 4.6  (0.3) |
|  |  |  |  |  |  |  |  |  |  |
| Gender composition of ill members |  |  |  |  |  |  |  |  |  |
| Male only | 40.0  (1.0) | 38.5  (1.1) | 34.1  (0.8) | 37.3  (1.3) | 32.9  (1.1) | 29.6  (0.6) | 16.5  (0.9) | 12.8  (0.8) | 12.0  (0.5) |
|  |  |  |  |  |  |  |  |  |  |
| Female only | 42.4  (1.0) | 41.2  (1.1) | 43.2  (0.8) | 43.6  (1.3) | 43.7  (1.2) | 44.0  (0.7) | 19.1  (1.0) | 21.3  (0.9) | 22.2  (0.6) |
|  |  |  |  |  |  |  |  |  |  |
| Male and female | 17.6  (0.8) | 20.3  (0.9) | 22.6  (0.7) | 19.1  (1.0) | 23.3  (1.1) | 26.5  (0.7) | 64.4  (1.2) | 65.9  (1.1) | 65.8  (0.8) |
|  |  |  |  |  |  |  |  |  |  |
| Number of ill members |  |  |  |  |  |  |  |  |  |
| One | 71.4  (0.9) | 68.6  (1.0) | 66.3  (0.8) | 77.3  (1.1) | 73.3  (1.2) | 70.2  (0.7) | 15.1  (0.9) | 15.6  (0.8) | 17.8  (0.7) |
|  |  |  |  |  |  |  |  |  |  |
| Two or more | 28.6  (0.9) | 31.4  (1.0) | 33.7  (0.8) | 22.7  (1.1) | 26.7  (1.2) | 29.8  (0.7) | 84.9  (0.9) | 84.4  (0.8) | 82.2  (0.7) |
|  |  |  |  |  |  |  |  |  |  |
| Comorbidity of ill members |  |  |  |  |  |  |  |  |  |
| One disease (no comorbidity) | 83.4  (0.7) | 90.7  (0.8) | 76.8  (1.2) | 97.6  (0.4) | 80.2  (1.0) | 70.3  (0.8) | 45.2  (1.3) | 36.5  (1.3) | 26.5  (0.7) |
|  |  |  |  |  |  |  |  |  |  |
| Two or more diseases | 16.6  (0.7) | 9.3  (0.8) | 23.2  (1.2) | 2.4  (0.4) | 19.8  (1.0) | 29.7  (0.8) | 54.8  (1.3) | 63.5  (1.3) | 73.5  (0.7) |

NCD = noncommunicable diseases

Numbers in parentheses are standard errors

Total number of households included in analysis: 10,075 in 2005, 12,237 in 2010, and 45,976 in 2016
