# Supplementary material for: Financial risk protection against noncommunicable diseases: trends and patterns in Bangladesh
Source: BMC Public Health. 2022 Sep 30;22:1835. doi: 10.1186/s12889-022-14243-0 (PMC9524135; doi:10.1186/s12889-022-14243-0)
Supplement: Supplementary file 6 — Additional file 6. [file 12889_2022_14243_MOESM6_ESM.docx]

**Additional file 6:** Alternative calculation of annual average out-of-pocket expenditure (in USD^a^)

**Alternative measurement approach A** [using OOP expenses (as a separate variable and as a component of total consumption expenditure) from the survey’s health module]

|  | Households affected by non-NCD only | | | Households affected by NCD only | | | Households affected by both NCD & non-NCD | | |
| --- | --- | --- | --- | --- | --- | --- | --- | --- | --- |
|  | 2005  (n = 2,875) | 2010  (n = 2,931) | 2016  (n = 10,391) | 2005  (n = 1,648) | 2010  (n = 2, 449) | 2016  (n = 9,393) | 2005  (n=1,806) | 2010  (n = 2,440) | 2016  (n = 10,160) |
|  |  |  |  |  |  |  |  |  |  |
| Overall | 144.5  (8.1) | 165.0  (9.8) | 199.1  (8.5) | 140.7  (15.6) | 185.7  (42.9) | 365.8  (13.3) | 188.4  (12.7) | 260.7  (14.9) | 429.9  (14.6) |
|  |  |  |  |  |  |  |  |  |  |
| Consumption expenditure quintile |  |  |  |  |  |  |  |  |  |
| Lowest | 34.7  (1.9) | 52.3  (3.1) | 57.6  (2.2) | 10.8  (2.5) | 17.2  (2.3) | 64.5  (2.2) | 40.0  (3.4) | 62.7  (4.8) | 92.3  (3.3) |
|  |  |  |  |  |  |  |  |  |  |
| 2nd | 66.7  (3.8) | 77.7  (4.5) | 100.8  (3.6) | 20.3  (2.9) | 38.8  (4.9) | 117.6  (4.0) | 62.9  (4.9) | 96.3  (6.2) | 165.9  (4.7) |
|  |  |  |  |  |  |  |  |  |  |
| 3rd | 99.0  (5.8) | 121.6  (7.0) | 154.7  (6.5) | 55.3  (8.8) | 60.8  (7.8) | 200.0  (10.3) | 101.9  (7.4) | 157.4  (8.8) | 247.0  (8.1) |
|  |  |  |  |  |  |  |  |  |  |
| 4th | 156.3  (9.7) | 191.1  (13.3) | 214.0  (13.2) | 95.5  (12.7) | 114.6  (13.5) | 326.4  (12.3) | 155.3  (10.0) | 248.9  (14.0) | 395.4  (12.4) |
|  |  |  |  |  |  |  |  |  |  |
| Highest | 404.6  (41.5) | 461.9  (52.4) | 525.1  (43.7) | 406.3  (56.5) | 537.3  (162.4) | 953.7  (51.1) | 463.5  (46.8) | 616.5  (51.1) | 935.3  (47.2) |
|  |  |  |  |  |  |  |  |  |  |
| Area of residence |  |  |  |  |  |  |  |  |  |
| Rural | 126.1  (7.3) | 165.7  (11.7) | 203.1  (10.6) | 125.0  (16.3) | 216.0  (60.7) | 348.0  (15.1) | 173.7  (14.6) | 260.9  (17.1) | 412.1  (15.9) |
|  |  |  |  |  |  |  |  |  |  |
| Urban | 209.7  (25.9) | 162.2  (16.1) | 189.9  (13.9) | 181.1  (36.6) | 118.0  (29.7) | 417.1  (28.1) | 235.5  (25.5) | 260.0  (28.4) | 485.0  (33.5) |
|  |  |  |  |  |  |  |  |  |  |
| Household head's education |  |  |  |  |  |  |  |  |  |
| No education | 128.6  (10.1) | 135.0  (9.2) | 183.7  (14.1) | 115.0  (18.0) | 107.0  (11.8) | 297.6  (15.8) | 139.7  (15.8) | 243.0  (20.2) | 326.4  (12.5) |
|  |  |  |  |  |  |  |  |  |  |
| Below secondary | 167.8  (17.0) | 194.6  (20.1) | 202.0  (10.3) | 139.7  (25.6) | 267.3  (130.8) | 356.0  (17.1) | 212.3  (23.2) | 275.0  (23.4) | 466.1  (23.5) |
|  |  |  |  |  |  |  |  |  |  |
| Secondary or above | 161.4  (20.6) | 211.8  (28.3) | 235.1  (23.6) | 219.2  (55.4) | 267.1  (89.7) | 569.2  (43.4) | 316.8  (39.8) | 296.4  (29.0) | 621.2  (41.6) |
|  |  |  |  |  |  |  |  |  |  |
| Illness of main income earner |  |  |  |  |  |  |  |  |  |
| No | 132.9  (7.8) | 163.6  (10.3) | 184.0  (7.4) | 146.5  (20.2) | 164.1  (30.8) | 372.8  (17.1) | 197.9  (18.0) | 297.8  (25.7) | 452.1  (21.8) |
|  |  |  |  |  |  |  |  |  |  |
| Yes | 181.1  (23.0) | 168.7  (19.9) | 243.6  (23.9) | 133.1  (24.5) | 215.0  (92.1) | 355.9  (16.6) | 181.3  (17.7) | 230.6  (14.2) | 410.6  (16.0) |
|  |  |  |  |  |  |  |  |  |  |
| Age composition of ill members |  |  |  |  |  |  |  |  |  |
| Children (<18 years) only | 109.1  (9.1) | 120.1  (9.7) | 153.6  (8.4) | 244.8  (89.4) | 194.5  (56.7) | 348.3  (67.1) | 109.6  (25.9) | 192.7  (51.3) | 194.3  (24.5) |
|  |  |  |  |  |  |  |  |  |  |
| Non-elderly adults (18-60 years) only | 170.6  (18.1) | 198.9  (22.0) | 201.8  (11.5) | 125.5  (15.5) | 148.9  (28.1) | 345.2  (15.2) | 221.1  (34.7) | 239.0  (20.3) | 410.3  (22.4) |
|  |  |  |  |  |  |  |  |  |  |
| Elderly (>60 years) only | 83.9  (12.2) | 111.5  (26.4) | 284.9  (114.0) | 105.1  (34.5) | 282.3  (199.4) | 385.9  (27.4) | 169.3  (57.2) | 186.8  (47.7) | 366.7  (30.3) |
|  |  |  |  |  |  |  |  |  |  |
| Children and non-elderly adults | 213.6  (24.7) | 201.9  (16.6) | 248.1  (19.3) | 404.3  (257.0) | 219.8  (66.2) | 354.6  (40.4) | 165.3  (15.3) | 253.0  (23.2) | 384.8  (15.6) |
|  |  |  |  |  |  |  |  |  |  |
| Non-elderly adults and elderly | 144.5  (39.0) | 150.4  (29.6) | 317.1  (59.2) | 192.9  (66.5) | 229.8  (86.3) | 466.1  (33.3) | 212.6  (32.1) | 331.6  (49.1) | 632.1  (84.8) |
|  |  |  |  |  |  |  |  |  |  |
| Children and elderly | 127.2  (30.7) | 169.5  (29.7) | 304.9  (81.1) | 8.1  (6.0) | 332.3  (157.8) | 563.3  (98.2) | 203.5  (48.7) | 299.4  (60.8) | 490.8  (58.7) |
|  |  |  |  |  |  |  |  |  |  |
| Gender composition of ill members |  |  |  |  |  |  |  |  |  |
| Male only | 139.2  (13.0) | 140.0  (14.6) | 185.2  (16.2) | 152.9  (25.6) | 238.8  (117.2) | 375.7  (21.4) | 160.9  (27.3) | 223.3  (33.3) | 367.8  (29.0) |
|  |  |  |  |  |  |  |  |  |  |
| Female only | 124.3  (9.8) | 165.7  (15.7) | 176.4  (9.5) | 112.7  (18.9) | 160.3  (39.4) | 313.7  (17.1) | 168.0  (28.3) | 223.0  (20.7) | 364.2  (24.1) |
|  |  |  |  |  |  |  |  |  |  |
| Male and female | 204.0  (26.4) | 210.9  (17.8) | 263.4  (18.7) | 180.7  (47.9) | 158.1  (29.9) | 441.1  (23.3) | 201.5  (16.4) | 280.2  (18.7) | 463.4  (18.4) |
|  |  |  |  |  |  |  |  |  |  |
| Number of ill members |  |  |  |  |  |  |  |  |  |
| One | 127.5  (8.5) | 153.3  (12.9) | 171.7  (9.7) | 132.8  (16.3) | 195.9  (57.7) | 327.2  (14.7) | 181.9  (36.2) | 186.4  (22.3) | 328.0  (26.5) |
|  |  |  |  |  |  |  |  |  |  |
| Two or more | 187.0  (18.7) | 190.4  (12.7) | 253.1  (15.5) | 167.5  (40.6) | 157.6  (27.2) | 456.4  (22.4) | 189.6  (13.5) | 274.5  (16.9) | 452.0  (16.5) |
|  |  |  |  |  |  |  |  |  |  |
| Comorbidity of ill members |  |  |  |  |  |  |  |  |  |
| One disease (no comorbidity) | 139.9  (8.5) | 156.1  (8.7) | 199.3  (9.7) | 132.5  (15.5) | 191.8  (52.7) | 346.6  (15.6) | 182.5  (21.7) | 240.6  (22.2) | 398.6  (22.5) |
|  |  |  |  |  |  |  |  |  |  |
| Two or more diseases | 167.7  (23.7) | 251.6  (53.5) | 198.6  (16.5) | 474.1  (147.8) | 160.7  (33.6) | 411.2  (21.2) | 193.2  (14.7) | 272.3  (18.4) | 441.2  (17.3) |

NCD = noncommunicable diseases

Numbers in parentheses are standard errors

^a^ All expenses in Bangladeshi taka (BDT) were expressed in 2016 prices using consumer price index, CPI (CPI_2005_ = 69.153, CPI_2010_ = 100, and CPI_2016_ = 152.529) and then converted into US dollars using the 2016 average exchange rate (USD 1 = BDT 78.468)

**Alternative measurement approach B:** [using OOP expenses (as a separate variable) from the survey’s health module, and the OOP component of total consumption expenditure (thus CTP) from the consumption module]

|  | Households affected by non-NCD only | | | Households affected by NCD only | | | Households affected by both NCD & non-NCD | | |
| --- | --- | --- | --- | --- | --- | --- | --- | --- | --- |
|  | 2005  (n = 2,875) | 2010  (n = 2,931) | 2016  (n = 10,391) | 2005  (n = 1,648) | 2010  (n = 2, 449) | 2016  (n = 9,393) | 2005  (n=1,806) | 2010  (n = 2,440) | 2016  (n = 10,160) |
|  |  |  |  |  |  |  |  |  |  |
| Overall | 144.5  (8.1) | 165.0  (9.8) | 199.1  (8.5) | 140.7  (15.6) | 185.7  (42.9) | 365.8  (13.3) | 188.4  (12.7) | 260.7  (14.9) | 429.9  (14.6) |
|  |  |  |  |  |  |  |  |  |  |
| Consumption expenditure quintile |  |  |  |  |  |  |  |  |  |
| Lowest | 68.2  (7.3) | 114.9  (21.2) | 134.3  (22.2) | 65.0  (21.0) | 42.4  (8.0) | 162.4  (12.0) | 110.1  (41.8) | 129.6  (17.5) | 190.8  (12.5) |
|  |  |  |  |  |  |  |  |  |  |
| 2nd | 119.7  (13.0) | 113.6  (10.1) | 146.3  (9.5) | 90.6  (22.7) | 77.1  (11.8) | 234.1  (15.9) | 106.0  (15.2) | 142.9  (14.6) | 283.4  (12.9) |
|  |  |  |  |  |  |  |  |  |  |
| 3rd | 129.9  (17.5) | 162.5  (19.9) | 195.1  (13.0) | 77.2  (16.0) | 130.1  (21.6) | 325.2  (23.0) | 112.2  (9.8) | 223.1  (21.2) | 357.3  (16.7) |
|  |  |  |  |  |  |  |  |  |  |
| 4th | 184.6  (22.8) | 209.1  (18.5) | 232.2  (17.1) | 143.5  (27.8) | 159.5  (31.4) | 382.8  (19.3) | 210.5  (25.8) | 291.5  (24.6) | 452.4  (23.6) |
|  |  |  |  |  |  |  |  |  |  |
| Highest | 249.7  (28.7) | 264.7  (31.9) | 309.7  (25.0) | 264.2  (49.2) | 407.3  (159.1) | 670.1  (45.4) | 338.2  (33.8) | 461.0  (47.7) | 718.3  (44.1) |
|  |  |  |  |  |  |  |  |  |  |
| Area of residence |  |  |  |  |  |  |  |  |  |
| Rural | 126.1  (7.3) | 165.7  (11.7) | 203.1  (10.6) | 125.0  (16.3) | 216.0  (60.7) | 348.0  (15.1) | 173.7  (14.6) | 260.9  (17.1) | 412.1  (15.9) |
|  |  |  |  |  |  |  |  |  |  |
| Urban | 209.7  (25.9) | 162.2  (16.1) | 189.9  (13.9) | 181.1  (36.6) | 118.0  (29.7) | 417.1  (28.1) | 235.5  (25.5) | 260.0  (28.4) | 485.0  (33.5) |
|  |  |  |  |  |  |  |  |  |  |
| Household head's education |  |  |  |  |  |  |  |  |  |
| No education | 128.6  (10.1) | 135.0  (9.2) | 183.7  (14.1) | 115.0  (18.0) | 107.0  (11.8) | 297.6  (15.8) | 139.7  (15.8) | 243.0  (20.2) | 326.4  (12.5) |
|  |  |  |  |  |  |  |  |  |  |
| Below secondary | 167.8  (17.0) | 194.6  (20.1) | 202.0  (10.3) | 139.7  (25.6) | 267.3  (130.8) | 356.0  (17.1) | 212.3  (23.2) | 275.0  (23.4) | 466.1  (23.5) |
|  |  |  |  |  |  |  |  |  |  |
| Secondary or above | 161.4  (20.6) | 211.8  (28.3) | 235.1  (23.6) | 219.2  (55.4) | 267.1  (89.7) | 569.2  (43.4) | 316.8  (39.8) | 296.4  (29.0) | 621.2  (41.6) |
|  |  |  |  |  |  |  |  |  |  |
| Illness of main income earner |  |  |  |  |  |  |  |  |  |
| No | 132.9  (7.8) | 163.6  (10.3) | 184.0  (7.4) | 146.5  (20.2) | 164.1  (30.8) | 372.8  (17.1) | 197.9  (18.0) | 297.8  (25.7) | 452.1  (21.8) |
|  |  |  |  |  |  |  |  |  |  |
| Yes | 181.1  (23.0) | 168.7  (19.9) | 243.6  (23.9) | 133.1  (24.5) | 215.0  (92.1) | 355.9  (16.6) | 181.3  (17.7) | 230.6  (14.2) | 410.6  (16.0) |
|  |  |  |  |  |  |  |  |  |  |
| Age composition of ill members |  |  |  |  |  |  |  |  |  |
| Children (<18 years) only | 109.1  (9.1) | 120.1  (9.7) | 153.6  (8.4) | 244.8  (89.4) | 194.5  (56.7) | 348.3  (67.1) | 109.6  (25.9) | 192.7  (51.3) | 194.3  (24.5) |
|  |  |  |  |  |  |  |  |  |  |
| Non-elderly adults (18-60 years) only | 170.6  (18.1) | 198.9  (22.0) | 201.8  (11.5) | 125.5  (15.5) | 148.9  (28.1) | 345.2  (15.2) | 221.1  (34.7) | 239.0  (20.3) | 410.3  (22.4) |
|  |  |  |  |  |  |  |  |  |  |
| Elderly (>60 years) only | 83.9  (12.2) | 111.5  (26.4) | 284.9  (114.0) | 105.1  (34.5) | 282.3  (199.4) | 385.9  (27.4) | 169.3  (57.2) | 186.8  (47.7) | 366.7  (30.3) |
|  |  |  |  |  |  |  |  |  |  |
| Children and non-elderly adults | 213.6  (24.7) | 201.9  (16.6) | 248.1  (19.3) | 404.3  (257.0) | 219.8  (66.2) | 354.6  (40.4) | 165.3  (15.3) | 253.0  (23.2) | 384.8  (15.6) |
|  |  |  |  |  |  |  |  |  |  |
| Non-elderly adults and elderly | 144.5  (39.0) | 150.4  (29.6) | 317.1  (59.2) | 192.9  (66.5) | 229.8  (86.3) | 466.1  (33.3) | 212.6  (32.1) | 331.6  (49.1) | 632.1  (84.8) |
|  |  |  |  |  |  |  |  |  |  |
| Children and elderly | 127.2  (30.7) | 169.5  (29.7) | 304.9  (81.1) | 8.1  (6.0) | 332.3  (157.8) | 563.3  (98.2) | 203.5  (48.7) | 299.4  (60.8) | 490.8  (58.7) |
|  |  |  |  |  |  |  |  |  |  |
| Gender composition of ill members |  |  |  |  |  |  |  |  |  |
| Male only | 139.2  (13.0) | 140.0  (14.6) | 185.2  (16.2) | 152.9  (25.6) | 238.8  (117.2) | 375.7  (21.4) | 160.9  (27.3) | 223.3  (33.3) | 367.8  (29.0) |
|  |  |  |  |  |  |  |  |  |  |
| Female only | 124.3  (9.8) | 165.7  (15.7) | 176.4  (9.5) | 112.7  (18.9) | 160.3  (39.4) | 313.7  (17.1) | 168.0  (28.3) | 223.0  (20.7) | 364.2  (24.1) |
|  |  |  |  |  |  |  |  |  |  |
| Male and female | 204.0  (26.4) | 210.9  (17.8) | 263.4  (18.7) | 180.7  (47.9) | 158.1  (29.9) | 441.1  (23.3) | 201.5  (16.4) | 280.2  (18.7) | 463.4  (18.4) |
|  |  |  |  |  |  |  |  |  |  |
| Number of ill members |  |  |  |  |  |  |  |  |  |
| One | 127.5  (8.5) | 153.3  (12.9) | 171.7  (9.7) | 132.8  (16.3) | 195.9  (57.7) | 327.2  (14.7) | 181.9  (36.2) | 186.4  (22.3) | 328.0  (26.5) |
|  |  |  |  |  |  |  |  |  |  |
| Two or more | 187.0  (18.7) | 190.4  (12.7) | 253.1  (15.5) | 167.5  (40.6) | 157.6  (27.2) | 456.4  (22.4) | 189.6  (13.5) | 274.5  (16.9) | 452.0  (16.5) |
|  |  |  |  |  |  |  |  |  |  |
| Comorbidity of ill members |  |  |  |  |  |  |  |  |  |
| One disease (no comorbidity) | 139.9  (8.5) | 156.1  (8.7) | 199.3  (9.7) | 132.5  (15.5) | 191.8  (52.7) | 346.6  (15.6) | 182.5  (21.7) | 240.6  (22.2) | 398.6  (22.5) |
|  |  |  |  |  |  |  |  |  |  |
| Two or more diseases | 167.7  (23.7) | 251.6  (53.5) | 198.6  (16.5) | 474.1  (147.8) | 160.7  (33.6) | 411.2  (21.2) | 193.2  (14.7) | 272.3  (18.4) | 441.2  (17.3) |

NCD = noncommunicable diseases, OOP = out-of-pocket, HIES = Household Income and Expenditure Survey, CTP = capacity-to-pay

Numbers in parentheses are standard errors

(OOP expenses in alternative measurement approach A and B differs only across consumption expenditure quintile)

^a^ All expenses in Bangladeshi taka (BDT) were expressed in 2016 prices using consumer price index, CPI (CPI_2005_ = 69.153, CPI_2010_ = 100, and CPI_2016_ = 152.529) and then converted into US dollars using the 2016 average exchange rate (USD 1 = BDT 78.468)
