# Supplementary material for: Financial risk protection against noncommunicable diseases: trends and patterns in Bangladesh
Source: BMC Public Health. 2022 Sep 30;22:1835. doi: 10.1186/s12889-022-14243-0 (PMC9524135; doi:10.1186/s12889-022-14243-0)
Supplement: Supplementary file 7 — Additional file 7. [file 12889_2022_14243_MOESM7_ESM.docx]

**Additional file 7:** Alternative calculation of the incidence of catastrophic health expenditure (%), normative food, housing (rent), and utilities method, 40% threshold

**Alternative measurement approach A** [using OOP expenses (as a separate variable and as a component of total consumption expenditure) from the survey’s health module]

|  | Households affected by non-NCD only | | | Households affected by NCD only | | | Households affected by both NCD & non-NCD | | |
| --- | --- | --- | --- | --- | --- | --- | --- | --- | --- |
|  | 2005  (n = 2,875) | 2010  (n = 2,931) | 2016  (n = 10,391) | 2005  (n = 1,648) | 2010  (n = 2, 449) | 2016  (n = 9,393) | 2005  (n=1,806) | 2010  (n = 2,440) | 2016  (n = 10,160) |
|  |  |  |  |  |  |  |  |  |  |
| Overall | 21.6  (0.8) | 21.0  (1.0) | 18.4  (0.8) | 8.3  (0.7) | 9.5  (0.7) | 23.5  (0.7) | 18.1  (0.9) | 19.3  (1.0) | 24.5  (0.7) |
|  |  |  |  |  |  |  |  |  |  |
| Consumption expenditure quintile |  |  |  |  |  |  |  |  |  |
| Lowest | 66.5  (2.1) | 65.9  (2.3) | 52.4  (1.5) | 15.2  (2.3) | 24.7  (2.2) | 59.6  (1.5) | 65.8  (3.0) | 69.3  (2.7) | 69.9  (1.6) |
|  |  |  |  |  |  |  |  |  |  |
| 2nd | 16.5  (1.6) | 11.6  (1.4) | 13.2  (0.9) | 6.8  (1.6) | 8.1  (1.5) | 16.2  (1.1) | 16.0  (2.2) | 16.1  (1.8) | 23.6  (1.2) |
|  |  |  |  |  |  |  |  |  |  |
| 3rd | 9.3  (1.3) | 7.2  (1.1) | 8.4  (0.7) | 5.3  (1.3) | 5.1  (1.1) | 14.5  (1.3) | 10.5  (1.7) | 9.3  (1.3) | 16.8  (1.1) |
|  |  |  |  |  |  |  |  |  |  |
| 4th | 7.9  (1.3) | 6.6  (1.2) | 7.9  (0.9) | 5.1  (1.3) | 4.8  (1.0) | 14.1  (1.2) | 6.9  (1.3) | 8.5  (1.3) | 15.6  (1.2) |
|  |  |  |  |  |  |  |  |  |  |
| Highest | 8.6  (1.4) | 9.0  (1.5) | 8.3  (1.0) | 9.2  (1.6) | 6.8  (1.1) | 16.8  (1.1) | 6.8  (1.3) | 8.9  (1.3) | 15.2  (1.2) |
|  |  |  |  |  |  |  |  |  |  |
| Area of residence |  |  |  |  |  |  |  |  |  |
| Rural | 23.9  (1.0) | 23.9  (1.3) | 22.2  (0.9) | 8.9  (0.9) | 12.1  (1.0) | 27.3  (0.8) | 20.3  (1.2) | 21.4  (1.1) | 27.6  (0.8) |
|  |  |  |  |  |  |  |  |  |  |
| Urban | 13.2  (1.3) | 10.5  (1.2) | 9.7  (1.0) | 6.6  (1.2) | 3.7  (0.6) | 12.7  (1.0) | 11.0  (1.3) | 10.2  (1.4) | 15.0  (1.1) |
|  |  |  |  |  |  |  |  |  |  |
| Household head's education |  |  |  |  |  |  |  |  |  |
| No education | 28.1  (1.2) | 27.0  (1.5) | 24.1  (1.2) | 10.8  (1.1) | 12.4  (1.1) | 30.6  (1.2) | 23.8  (1.4) | 25.5  (1.4) | 29.0  (1.1) |
|  |  |  |  |  |  |  |  |  |  |
| Below secondary | 15.7  (1.3) | 16.9  (1.4) | 16.7  (0.9) | 6.4  (1.2) | 8.7  (1.1) | 20.6  (0.9) | 12.8  (1.5) | 14.3  (1.5) | 24.4  (0.9) |
|  |  |  |  |  |  |  |  |  |  |
| Secondary or above | 6.3  (1.4) | 7.5  (1.4) | 7.2  (1.2) | 4.0  (1.4) | 3.1  (0.9) | 11.8  (1.1) | 8.5  (1.9) | 7.1  (1.4) | 11.8  (1.2) |
|  |  |  |  |  |  |  |  |  |  |
| Age composition of ill members |  |  |  |  |  |  |  |  |  |
| Children (<18 years) only | 18.5  (1.1) | 21.5  (1.6) | 14.9  (0.8) | 20.4  (4.8) | 22.0  (4.0) | 28.8  (5.8) | 17.6  (4.8) | 24.1  (5.8) | 19.0  (2.7) |
|  |  |  |  |  |  |  |  |  |  |
| Non-elderly adults (18-60 years) only | 23.1  (1.5) | 21.2  (1.5) | 18.6  (1.0) | 7.4  (0.8) | 8.6  (0.8) | 20.8  (0.8) | 17.6  (1.9) | 19.3  (1.6) | 23.9  (1.1) |
|  |  |  |  |  |  |  |  |  |  |
| Elderly (>60 years) only | 28.0  (3.7) | 24.2  (4.2) | 29.6  (2.7) | 7.1  (1.6) | 7.7  (1.3) | 32.6  (1.3) | 21.4  (5.0) | 31.2  (4.1) | 41.6  (2.9) |
|  |  |  |  |  |  |  |  |  |  |
| Children and non-elderly adults | 25.1  (2.3) | 19.2  (1.9) | 21.5  (1.5) | 17.8  (6.7) | 13.6  (4.0) | 19.4  (2.4) | 19.0  (1.5) | 18.3  (1.4) | 22.2  (1.0) |
|  |  |  |  |  |  |  |  |  |  |
| Non-elderly adults and elderly | 12.6  (6.7) | 27.0  (7.5) | 28.1  (5.6) | 6.7  (2.5) | 10.9  (2.6) | 21.4  (1.7) | 22.0  (3.3) | 18.1  (2.4) | 29.4  (1.9) |
|  |  |  |  |  |  |  |  |  |  |
| Children and elderly | 29.8  (9.1) | 2.1  (2.1) | 31.0  (7.3) | 47.6  (35.3) | 18.7  (13.1) | 15.8  (8.5) | 15.4  (3.8) | 12.1  (3.2) | 19.5  (2.3) |
|  |  |  |  |  |  |  |  |  |  |
| Illness of household's main income earner |  |  |  |  |  |  |  |  |  |
| No | 19.5  (0.9) | 20.4  (1.2) | 16.6  (0.8) | 8.1  (1.0) | 10.2  (0.9) | 24.8  (0.9) | 16.7  (1.4) | 20.1  (1.4) | 24.5  (1.0) |
|  |  |  |  |  |  |  |  |  |  |
| Yes | 28.1  (1.9) | 22.7  (1.8) | 23.7  (1.5) | 8.5  (1.1) | 8.4  (1.0) | 21.7  (0.9) | 19.2  (1.3) | 18.7  (1.2) | 24.5  (0.9) |
|  |  |  |  |  |  |  |  |  |  |
| Gender composition of ill members |  |  |  |  |  |  |  |  |  |
| Male only | 21.4  (1.3) | 21.6  (1.6) | 18.1  (0.9) | 9.8  (1.3) | 9.2  (1.2) | 24.3  (1.3) | 20.4  (2.5) | 17.6  (2.4) | 23.9  (1.6) |
|  |  |  |  |  |  |  |  |  |  |
| Female only | 21.5  (1.3) | 21.5  (1.6) | 17.1  (0.9) | 7.3  (1.0) | 10.8  (1.0) | 24.6  (1.0) | 21.1  (2.3) | 23.7  (2.2) | 28.2  (1.3) |
|  |  |  |  |  |  |  |  |  |  |
| Male and female | 22.0  (2.0) | 19.1  (1.7) | 21.5  (1.4) | 7.6  (1.6) | 7.5  (1.2) | 20.8  (1.1) | 16.6  (1.2) | 18.3  (1.1) | 23.4  (0.8) |
|  |  |  |  |  |  |  |  |  |  |
| Number of ill members |  |  |  |  |  |  |  |  |  |
| One | 21.4  (1.0) | 21.3  (1.2) | 17.4  (0.8) | 8.4  (0.8) | 10.1  (0.8) | 24.4  (0.9) | 21.7  (2.6) | 27.7  (2.6) | 30.3  (1.5) |
|  |  |  |  |  |  |  |  |  |  |
| Two or more | 22.0  (1.6) | 20.5  (1.5) | 20.4  (1.2) | 7.8  (1.5) | 7.7  (1.2) | 21.5  (1.1) | 17.5  (1.0) | 17.8  (1.0) | 23.3  (0.8) |
|  |  |  |  |  |  |  |  |  |  |
| Comorbidity of ill members |  |  |  |  |  |  |  |  |  |
| One disease (no comorbidity) | 20.4  (0.9) | 20.5  (1.1) | 18.1  (0.8) | 8.2  (0.7) | 9.9  (0.8) | 23.8  (0.8) | 15.3  (1.3) | 16.2  (1.3) | 22.8  (1.1) |
|  |  |  |  |  |  |  |  |  |  |
| Two or more diseases | 27.7  (2.2) | 26.3  (2.8) | 19.5  (1.6) | 12.9  (6.7) | 7.7  (1.2) | 22.7  (1.1) | 20.4  (1.4) | 21.2  (1.3) | 25.2  (0.8) |

NCD = noncommunicable diseases, OOP = out-of-pocket, CTP = capacity to-pay

Numbers in parentheses are standard errors

**Alternative measurement approach B** [using OOP expenses (as a separate variable) from the survey’s health module, and the OOP component of total consumption expenditure (thus CTP) from the consumption module]

|  | Households affected by non-NCD only | | | Households affected by NCD only | | | Households affected by both NCD & non-NCD | | |
| --- | --- | --- | --- | --- | --- | --- | --- | --- | --- |
|  | 2005  (n = 2,875) | 2010  (n = 2,931) | 2016  (n = 10,391) | 2005  (n = 1,648) | 2010  (n = 2, 449) | 2016  (n = 9,393) | 2005  (n=1,806) | 2010  (n = 2,440) | 2016  (n = 10,160) |
|  |  |  |  |  |  |  |  |  |  |
| Overall | 25.5  (0.9) | 23.6  (1.0) | 21.6  (0.7) | 10.1  (0.8) | 10.7  (0.7) | 27.3  (0.7) | 20.4  (1.0) | 23.0  (1.1) | 29.5  (0.8) |
|  |  |  |  |  |  |  |  |  |  |
| Consumption expenditure quintile |  |  |  |  |  |  |  |  |  |
| Lowest | 73.9  (1.9) | 71.3  (2.1) | 60.7  (1.4) | 23.3  (2.7) | 31.2  (2.5) | 68.2  (1.3) | 73.1  (2.8) | 74.4  (2.5) | 78.0  (1.4) |
|  |  |  |  |  |  |  |  |  |  |
| 2nd | 24.5  (1.8) | 14.8  (1.4) | 18.5  (1.1) | 12.3  (2.1) | 10.2  (1.6) | 26.5  (1.4) | 20.7  (2.4) | 21.3  (2.1) | 36.3  (1.5) |
|  |  |  |  |  |  |  |  |  |  |
| 3rd | 9.9  (1.3) | 8.6  (1.3) | 11.1  (1.1) | 7.3  (1.5) | 8.2  (1.3) | 20.2  (1.4) | 10.8  (1.7) | 15.1  (1.8) | 24.0  (1.5) |
|  |  |  |  |  |  |  |  |  |  |
| 4th | 7.3  (1.2) | 7.8  (1.3) | 7.7  (0.9) | 5.8  (1.4) | 6.7  (1.2) | 15.2  (1.1) | 9.2  (1.7) | 10.9  (1.5) | 16.2  (1.1) |
|  |  |  |  |  |  |  |  |  |  |
| Highest | 4.4  (1.1) | 4.0  (0.9) | 4.9  (0.9) | 6.1  (1.4) | 3.0  (0.7) | 9.9  (1.0) | 4.2  (1.0) | 5.9  (1.1) | 10.5  (1.0) |
|  |  |  |  |  |  |  |  |  |  |
|  |  |  |  |  |  |  |  |  |  |
| Area of residence |  |  |  |  |  |  |  |  |  |
| Rural | 28.1  (1.0) | 26.6  (1.3) | 25.3  (0.9) | 10.8  (1.0) | 13.6  (1.0) | 31.2  (0.8) | 22.4  (1.2) | 24.7  (1.2) | 32.7  (0.9) |
|  |  |  |  |  |  |  |  |  |  |
| Urban | 16.2  (1.5) | 12.6  (1.2) | 12.9  (1.2) | 8.4  (1.4) | 4.4  (0.7) | 16.1  (1.2) | 14.2  (1.6) | 15.4  (2.3) | 19.5  (1.4) |
|  |  |  |  |  |  |  |  |  |  |
|  |  |  |  |  |  |  |  |  |  |
| Household head's education |  |  |  |  |  |  |  |  |  |
| No education | 32.8  (1.2) | 29.4  (1.5) | 27.3  (1.1) | 12.3  (1.2) | 13.4  (1.0) | 33.7  (1.1) | 25.2  (1.5) | 28.2  (1.4) | 33.6  (1.1) |
|  |  |  |  |  |  |  |  |  |  |
| Below secondary | 18.8  (1.4) | 19.9  (1.5) | 19.8  (0.9) | 8.8  (1.4) | 9.9  (1.2) | 24.5  (1.0) | 15.9  (1.7) | 19.3  (1.7) | 29.6  (1.1) |
|  |  |  |  |  |  |  |  |  |  |
| Secondary or above | 8.3  (1.6) | 10.0  (1.7) | 10.5  (1.4) | 6.2  (1.7) | 5.0  (1.1) | 17.1  (1.8) | 12.5  (2.3) | 11.2  (1.9) | 17.0  (1.5) |
|  |  |  |  |  |  |  |  |  |  |
|  |  |  |  |  |  |  |  |  |  |
| Illness of household's main income earner |  |  |  |  |  |  |  |  |  |
| No | 22.9  (0.9) | 22.8  (1.2) | 19.6  (0.8) | 10.4  (1.1) | 11.5  (0.9) | 29.0  (0.9) | 19.7  (1.5) | 23.4  (1.5) | 29.5  (1.0) |
|  |  |  |  |  |  |  |  |  |  |
| Yes | 33.6  (2.0) | 26.0  (1.8) | 27.3  (1.5) | 9.8  (1.2) | 9.7  (1.1) | 24.9  (1.0) | 21.0  (1.4) | 22.6  (1.3) | 29.4  (1.0) |
|  |  |  |  |  |  |  |  |  |  |
|  |  |  |  |  |  |  |  |  |  |
| Age composition of ill members |  |  |  |  |  |  |  |  |  |
| Children (<18 years) only | 21.5  (1.2) | 23.1  (1.5) | 17.8  (0.9) | 26.1  (5.2) | 25.0  (4.2) | 33.0  (5.6) | 24.4  (5.5) | 26.4  (5.9) | 22.0  (3.0) |
|  |  |  |  |  |  |  |  |  |  |
| Non-elderly adults (18-60 years) only | 27.4  (1.6) | 23.5  (1.6) | 21.5  (1.1) | 9.1  (0.9) | 9.5  (0.8) | 24.8  (0.8) | 20.6  (2.0) | 23.8  (1.7) | 29.5  (1.2) |
|  |  |  |  |  |  |  |  |  |  |
| Elderly (>60 years) only | 31.0  (3.8) | 30.2  (4.4) | 32.2  (2.7) | 7.9  (1.7) | 9.2  (1.4) | 34.9  (1.4) | 28.7  (5.7) | 32.9  (4.1) | 44.9  (3.0) |
|  |  |  |  |  |  |  |  |  |  |
| Children and non-elderly adults | 31.4  (2.5) | 23.0  (2.2) | 25.3  (1.6) | 20.4  (7.0) | 14.8  (4.2) | 22.6  (2.5) | 20.4  (1.6) | 21.5  (1.6) | 27.2  (1.1) |
|  |  |  |  |  |  |  |  |  |  |
| Non-elderly adults and elderly | 12.6  (6.7) | 29.9  (7.7) | 34.5  (5.8) | 9.2  (2.9) | 12.0  (2.7) | 27.3  (1.9) | 23.3  (3.3) | 22.1  (2.5) | 34.8  (2.0) |
|  |  |  |  |  |  |  |  |  |  |
| Children and elderly | 33.8  (9.4) | 9.3  (7.2) | 35.0  (7.4) | 47.6  (35.3) | 31.6  (15.9) | 28.3  (12.2) | 19.5  (4.2) | 17.4  (3.8) | 21.4  (2.3) |
|  |  |  |  |  |  |  |  |  |  |
|  |  |  |  |  |  |  |  |  |  |
| Gender composition of ill members |  |  |  |  |  |  |  |  |  |
| Male only | 25.7  (1.4) | 23.8  (1.6) | 20.3  (1.0) | 12.2  (1.5) | 10.6  (1.2) | 27.7  (1.1) | 23.4  (2.6) | 21.6  (2.6) | 28.2  (1.8) |
|  |  |  |  |  |  |  |  |  |  |
| Female only | 24.5  (1.3) | 23.9  (1.6) | 20.1  (0.9) | 8.8  (1.1) | 11.7  (1.1) | 28.6  (1.0) | 22.8  (2.4) | 27.0  (2.2) | 32.7  (1.4) |
|  |  |  |  |  |  |  |  |  |  |
| Male and female | 27.1  (2.1) | 22.9  (1.9) | 26.1  (1.5) | 9.3  (1.8) | 9.1  (1.3) | 24.6  (1.2) | 19.0  (1.2) | 22.0  (1.2) | 28.6  (0.9) |
|  |  |  |  |  |  |  |  |  |  |
|  |  |  |  |  |  |  |  |  |  |
| Number of ill members |  |  |  |  |  |  |  |  |  |
| One | 24.9  (1.0) | 23.5  (1.2) | 19.7  (0.8) | 10.4  (0.9) | 11.3  (0.8) | 28.2  (0.8) | 27.4  (2.9) | 31.0  (2.6) | 34.4  (1.6) |
|  |  |  |  |  |  |  |  |  |  |
| Two or more | 27.0  (1.7) | 24.0  (1.6) | 25.3  (1.3) | 9.3  (1.7) | 9.1  (1.3) | 25.2  (1.1) | 19.2  (1.1) | 21.5  (1.1) | 28.4  (0.8) |
|  |  |  |  |  |  |  |  |  |  |
|  |  |  |  |  |  |  |  |  |  |
| Comorbidity of ill members |  |  |  |  |  |  |  |  |  |
| One disease (no comorbidity) | 23.6  (0.9) | 22.7  (1.1) | 21.1  (0.7) | 9.7  (0.8) | 11.1  (0.8) | 27.5  (0.8) | 17.0  (1.4) | 19.7  (1.5) | 28.1  (1.2) |
|  |  |  |  |  |  |  |  |  |  |
| Two or more diseases | 34.6  (2.3) | 32.5  (3.0) | 23.2  (1.8) | 27.7  (8.8) | 9.1  (1.3) | 26.9  (1.2) | 23.2  (1.4) | 24.9  (1.3) | 29.9  (0.9) |

NCD = noncommunicable diseases, OOP = out-of-pocket, CTP = capacity-to-pay

Numbers in parentheses are standard errors
