# Supplementary material for: Financial risk protection against noncommunicable diseases: trends and patterns in Bangladesh
Source: BMC Public Health. 2022 Sep 30;22:1835. doi: 10.1186/s12889-022-14243-0 (PMC9524135; doi:10.1186/s12889-022-14243-0)
Supplement: Supplementary file 8 — Additional file 8. [file 12889_2022_14243_MOESM8_ESM.docx]

**Additional file 8**: Alternative calculation of the incidence of catastrophic health expenditure (%), budget share method, 10% threshold

**Alternative measurement approach A** [using OOP expenses (as a separate variable and as a component of total consumption expenditure) from the survey’s health module]

|  | Households affected by non-NCD only | | | Households affected by NCD only | | | Households affected by both NCD & non-NCD | | |
| --- | --- | --- | --- | --- | --- | --- | --- | --- | --- |
|  | 2005  (n = 2,875) | 2010  (n = 2,931) | 2016  (n = 10,391) | 2005  (n = 1,648) | 2010  (n = 2, 449) | 2016  (n = 9,393) | 2005  (n=1,806) | 2010  (n = 2,440) | 2016  (n = 10,160) |
|  |  |  |  |  |  |  |  |  |  |
| Overall | 18.2  (0.8) | 16.1  (0.8) | 21.8  (0.7) | 12.2  (0.9) | 10.6  (0.8) | 34.5  (0.7) | 19.8  (1.0) | 24.4  (1.1) | 41.2  (1.0) |
|  |  |  |  |  |  |  |  |  |  |
| Consumption expenditure quintile |  |  |  |  |  |  |  |  |  |
| Lowest | 12.1  (1.5) | 13.6  (1.7) | 17.6  (1.0) | 5.3  (1.4) | 5.4  (1.1) | 26.8  (1.4) | 14.5  (2.3) | 17.6  (2.2) | 34.7  (1.7) |
|  |  |  |  |  |  |  |  |  |  |
| 2nd | 17.4  (1.6) | 11.3  (1.4) | 21.0  (1.1) | 7.3  (1.6) | 7.6  (1.4) | 26.5  (1.3) | 15.1  (2.2) | 18.8  (2.1) | 37.5  (1.5) |
|  |  |  |  |  |  |  |  |  |  |
| 3rd | 19.0  (1.7) | 17.8  (1.7) | 23.6  (1.4) | 11.7  (1.9) | 10.1  (1.6) | 33.7  (1.9) | 19.5  (2.2) | 23.5  (2.0) | 38.7  (1.6) |
|  |  |  |  |  |  |  |  |  |  |
| 4th | 21.3  (1.9) | 18.6  (1.8) | 22.5  (1.6) | 14.3  (2.1) | 13.6  (1.8) | 39.6  (1.7) | 22.7  (2.3) | 30.0  (2.2) | 45.0  (1.6) |
|  |  |  |  |  |  |  |  |  |  |
| Highest | 21.3  (2.0) | 20.8  (2.3) | 25.0  (2.0) | 18.9  (2.1) | 13.9  (1.6) | 43.2  (2.0) | 23.9  (2.2) | 28.8  (2.2) | 45.4  (2.4) |
|  |  |  |  |  |  |  |  |  |  |
| Area of residence |  |  |  |  |  |  |  |  |  |
| Rural | 18.2  (0.9) | 16.7  (1.0) | 23.6  (0.8) | 12.5  (1.0) | 12.8  (1.0) | 35.9  (0.9) | 19.8  (1.2) | 25.2  (1.2) | 42.7  (1.1) |
|  |  |  |  |  |  |  |  |  |  |
| Urban | 18.1  (1.7) | 13.9  (1.5) | 17.8  (1.2) | 11.3  (1.6) | 5.6  (0.8) | 30.7  (1.4) | 19.8  (2.1) | 21.2  (2.8) | 36.5  (2.2) |
|  |  |  |  |  |  |  |  |  |  |
| Household head's education |  |  |  |  |  |  |  |  |  |
| No education | 19.3  (1.1) | 16.0  (1.1) | 23.2  (0.9) | 11.8  (1.2) | 11.0  (1.0) | 35.0  (1.1) | 19.6  (1.4) | 24.6  (1.4) | 39.8  (1.3) |
|  |  |  |  |  |  |  |  |  |  |
| Below secondary | 18.5  (1.4) | 17.9  (1.5) | 21.7  (0.9) | 12.9  (1.6) | 11.5  (1.4) | 34.5  (1.1) | 19.8  (1.9) | 25.2  (1.9) | 42.6  (1.2) |
|  |  |  |  |  |  |  |  |  |  |
| Secondary or above | 12.3  (1.9) | 12.1  (1.9) | 18.0  (1.8) | 12.1  (2.2) | 7.9  (1.4) | 33.2  (1.9) | 20.9  (2.8) | 22.2  (2.6) | 40.5  (2.8) |
|  |  |  |  |  |  |  |  |  |  |
| Illness of main income earner |  |  |  |  |  |  |  |  |  |
| No | 16.0  (0.8) | 14.9  (0.9) | 19.6  (0.8) | 12.8  (1.2) | 11.3  (1.0) | 35.7  (0.9) | 20.4  (1.6) | 26.0  (1.4) | 40.4  (1.3) |
|  |  |  |  |  |  |  |  |  |  |
| Yes | 25.0  (1.8) | 19.4  (1.7) | 28.3  (1.6) | 11.4  (1.3) | 9.6  (1.1) | 33.0  (1.1) | 19.4  (1.4) | 23.2  (1.4) | 41.9  (1.1) |
|  |  |  |  |  |  |  |  |  |  |
| Age composition of ill members |  |  |  |  |  |  |  |  |  |
| Children (<18 years) only | 12.6  (1.0) | 10.3  (1.0) | 16.3  (1.0) | 22.8  (5.0) | 20.8  (4.2) | 36.0  (5.9) | 21.5  (5.3) | 21.0  (5.6) | 19.4  (2.9) |
|  |  |  |  |  |  |  |  |  |  |
| Non-elderly adults (18-60 years) only | 20.9  (1.5) | 18.9  (1.5) | 23.3  (1.0) | 11.7  (1.0) | 9.7  (0.8) | 31.5  (0.8) | 22.7  (2.1) | 25.0  (1.9) | 41.1  (1.8) |
|  |  |  |  |  |  |  |  |  |  |
| Elderly (>60 years) only | 15.2  (3.0) | 14.8  (3.5) | 25.5  (2.5) | 9.8  (1.9) | 8.3  (1.4) | 38.8  (1.5) | 19.5  (5.0) | 25.2  (3.6) | 50.9  (3.1) |
|  |  |  |  |  |  |  |  |  |  |
| Children and non-elderly adults | 29.2  (2.5) | 22.7  (2.1) | 28.0  (1.8) | 22.9  (7.5) | 10.2  (3.6) | 37.5  (3.1) | 17.5  (1.5) | 22.4  (1.5) | 39.4  (1.2) |
|  |  |  |  |  |  |  |  |  |  |
| Non-elderly adults and elderly | 26.2  (9.2) | 18.5  (6.2) | 37.0  (5.5) | 10.7  (3.0) | 15.1  (2.9) | 43.8  (2.2) | 24.0  (3.4) | 27.6  (2.7) | 48.4  (2.1) |
|  |  |  |  |  |  |  |  |  |  |
| Children and elderly | 21.0  (8.0) | 15.1  (8.9) | 25.8  (6.7) | 0.0  (n/o) | 39.4  (16.9) | 63.5  (11.0) | 23.8  (4.7) | 23.4  (4.2) | 35.2  (3.0) |
|  |  |  |  |  |  |  |  |  |  |
| Gender composition of ill members |  |  |  |  |  |  |  |  |  |
| Male only | 16.7  (1.2) | 15.2  (1.3) | 20.1  (1.0) | 13.8  (1.5) | 10.5  (1.2) | 33.4  (1.6) | 19.5  (2.5) | 19.8  (2.6) | 36.1  (2.2) |
|  |  |  |  |  |  |  |  |  |  |
| Female only | 15.8  (1.1) | 15.1  (1.3) | 19.4  (0.8) | 10.8  (1.3) | 10.2  (1.1) | 32.7  (1.0) | 17.6  (2.2) | 23.8  (1.9) | 40.6  (2.0) |
|  |  |  |  |  |  |  |  |  |  |
| Male and female | 26.9  (2.2) | 19.9  (1.9) | 29.2  (1.7) | 12.2  (2.0) | 11.4  (1.4) | 38.9  (1.4) | 20.6  (1.3) | 25.5  (1.3) | 42.3  (1.0) |
|  |  |  |  |  |  |  |  |  |  |
| Number of ill members |  |  |  |  |  |  |  |  |  |
| One | 16.2  (0.9) | 14.6  (0.9) | 19.1  (0.7) | 12.2  (1.0) | 10.4  (0.9) | 32.3  (1.0) | 22.4  (2.7) | 24.9  (2.5) | 40.9  (2.6) |
|  |  |  |  |  |  |  |  |  |  |
| Two or more | 23.1  (1.6) | 19.5  (1.5) | 27.2  (1.4) | 12.1  (1.9) | 11.0  (1.4) | 39.9  (1.3) | 19.4  (1.1) | 24.4  (1.2) | 41.2  (0.9) |
|  |  |  |  |  |  |  |  |  |  |
| Comorbidity of ill members |  |  |  |  |  |  |  |  |  |
| One disease (no comorbidity) | 16.6  (0.8) | 15.0  (0.9) | 21.3  (0.7) | 11.5  (0.9) | 10.4  (0.8) | 32.3  (0.9) | 16.5  (1.4) | 20.3  (1.8) | 37.1  (1.4) |
|  |  |  |  |  |  |  |  |  |  |
| Two or more diseases | 25.7  (2.2) | 26.6  (3.0) | 23.5  (1.7) | 41.8  (9.4) | 11.0  (1.5) | 39.9  (1.3) | 22.6  (1.5) | 26.8  (1.3) | 42.6  (1.1) |

NCD = noncommunicable diseases, OOP = out-of-pocket, n/o = no observations

Numbers in parentheses are standard errors

**Alternative measurement approach B** [using OOP expenses (as a separate variable) from the survey’s health module, and the OOP component of total consumption expenditure from the consumption module]

|  | Households affected by non-NCD only | | | | Households affected by NCD only | | | | Households affected by both NCD & non-NCD | | |
| --- | --- | --- | --- | --- | --- | --- | --- | --- | --- | --- | --- |
|  | 2005  (n = 2,875) | 2010  (n = 2,931) | 2016  (n = 10,391) | 2005  (n = 1,648) | | 2010  (n = 2, 449) | 2016  (n = 9,393) | 2005  (n=1,806) | | 2010  (n = 2,440) | 2016  (n = 10,160) |
|  |  |  |  |  | |  |  |  | |  |  |
| Overall | 19.7  (0.8) | 17.5  (0.9) | 23.3  (0.7) | 12.2  (0.9) | | 11.1  (0.8) | 35.3  (0.8) | 21.1  (1.0) | | 25.9  (1.1) | 42.6  (1.0) |
|  |  |  |  |  | |  |  |  | |  |  |
| Consumption expenditure quintile |  |  |  |  | |  |  |  | |  |  |
| Lowest | 22.4  (1.8) | 23.5  (1.9) | 27.6  (1.2) | 12.4  (2.1) | | 10.5  (1.6) | 37.5  (1.4) | 26.0  (2.8) | | 28.0  (2.6) | 48.6  (1.7) |
|  |  |  |  |  | |  |  |  | |  |  |
| 2nd | 23.3  (1.8) | 15.2  (1.6) | 24.9  (1.3) | 12.2  (2.1) | | 12.0  (1.7) | 34.7  (1.4) | 20.7  (2.4) | | 23.4  (2.2) | 45.5  (1.6) |
|  |  |  |  |  | |  |  |  | |  |  |
| 3rd | 17.2  (1.7) | 18.3  (1.8) | 25.0  (1.6) | 11.5  (1.9) | | 13.5  (1.7) | 35.3  (1.9) | 22.2  (2.3) | | 27.3  (2.2) | 45.1  (1.7) |
|  |  |  |  |  | |  |  |  | |  |  |
| 4th | 19.0  (1.9) | 17.0  (1.8) | 21.3  (1.6) | 12.9  (2.0) | | 12.6  (1.6) | 36.2  (1.6) | 20.5  (2.3) | | 30.1  (2.2) | 40.8  (1.7) |
|  |  |  |  |  | |  |  |  | |  |  |
| Highest | 15.3  (1.9) | 11.5  (1.7) | 16.3  (1.5) | 12.0  (1.8) | | 7.9  (1.1) | 33.3  (1.8) | 18.0  (2.0) | | 21.0  (1.9) | 36.4  (2.6) |
|  |  |  |  |  | |  |  |  | |  |  |
| Area of residence |  |  |  |  | |  |  |  | |  |  |
| Rural | 19.7  (0.9) | 18.2  (1.0) | 25.0  (0.8) | 12.4  (1.0) | | 13.5  (1.0) | 36.7  (0.9) | 21.2  (1.2) | | 26.7  (1.2) | 44.1  (1.1) |
|  |  |  |  |  | |  |  |  | |  |  |
| Urban | 19.7  (1.8) | 14.8  (1.5) | 19.4  (1.4) | 11.6  (1.6) | | 5.7  (0.8) | 31.5  (1.4) | 20.9  (2.1) | | 22.1  (2.8) | 37.9  (2.7) |
|  |  |  |  |  | |  |  |  | |  |  |
| Household head's education |  |  |  |  | |  |  |  | |  |  |
| No education | 20.7  (1.1) | 17.4  (1.1) | 24.5  (1.0) | 12.0  (1.2) | | 11.4  (1.0) | 35.7  (1.1) | 20.4  (1.4) | | 26.2  (1.4) | 41.1  (1.3) |
|  |  |  |  |  | |  |  |  | |  |  |
| Below secondary | 20.2  (1.5) | 19.4  (1.5) | 23.5  (0.9) | 12.9  (1.6) | | 12.7  (1.4) | 35.4  (1.1) | 21.6  (1.9) | | 26.7  (1.9) | 44.2  (1.2) |
|  |  |  |  |  | |  |  |  | |  |  |
| Secondary or above | 14.1  (2.0) | 13.2  (2.0) | 19.1  (1.8) | 11.5  (2.1) | | 7.8  (1.4) | 34.1  (1.9) | 22.7  (2.9) | | 22.9  (2.6) | 41.9  (2.8) |
|  |  |  |  |  | |  |  |  | |  |  |
| Illness of main income earner |  |  |  |  | |  |  |  | |  |  |
| No | 17.3  (0.9) | 16.2  (0.9) | 20.9  (0.8) | 12.8  (1.2) | | 12.0  (1.0) | 36.5  (0.9) | 21.8  (1.6) | | 27.6  (1.5) | 41.8  (1.4) |
|  |  |  |  |  | |  |  |  | |  |  |
| Yes | 27.4  (1.9) | 20.9  (1.7) | 30.3  (1.6) | 11.4  (1.3) | | 9.9  (1.1) | 33.7  (1.1) | 20.7  (1.4) | | 24.5  (1.4) | 43.2  (1.1) |
|  |  |  |  |  | |  |  |  | |  |  |
| Age composition of ill members |  |  |  |  | |  |  |  | |  |  |
| Children (<18 years) only | 13.4  (1.0) | 11.4  (1.1) | 17.2  (1.0) | 23.2  (5.0) | | 23.8  (4.4) | 37.1  (5.8) | 24.3  (5.5) | | 24.0  (6.0) | 20.8  (3.0) |
|  |  |  |  |  | |  |  |  | |  |  |
| Non-elderly adults (18-60 years) only | 23.2  (1.5) | 20.4  (1.5) | 25.3  (1.1) | 11.6  (1.0) | | 10.1  (0.8) | 32.2  (0.9) | 23.5  (2.1) | | 26.6  (1.9) | 42.3  (1.8) |
|  |  |  |  |  | |  |  |  | |  |  |
| Elderly (>60 years) only | 17.3  (3.1) | 15.2  (3.5) | 25.6  (2.5) | 10.2  (1.9) | | 9.3  (1.4) | 39.7  (1.5) | 20.1  (5.0) | | 26.1  (3.6) | 51.3  (3.1) |
|  |  |  |  |  | |  |  |  | |  |  |
| Children and non-elderly adults | 31.7  (2.5) | 24.8  (2.1) | 29.8  (1.8) | 22.9  (7.5) | | 10.2  (3.6) | 38.2  (3.1) | 19.1  (1.6) | | 23.4  (1.5) | 41.2  (1.4) |
|  |  |  |  |  | |  |  |  | |  |  |
| Non-elderly adults and elderly | 26.2  (9.2) | 21.5  (6.6) | 38.6  (5.5) | 10.7  (3.0) | | 14.7  (2.9) | 44.7  (2.2) | 24.8  (3.4) | | 29.9  (2.8) | 49.7  (2.1) |
|  |  |  |  |  | |  |  |  | |  |  |
| Children and elderly | 21.0  (0.8) | 15.1  (8.9) | 25.8  (6.7) | 0.0  (n/o) | | 39.4  (16.9) | 71.9  (9.5) | 25.0  (0.6) | | 26.6  (4.4) | 36.5  (3.1) |
|  |  |  |  |  | |  |  |  | |  |  |
| Gender composition of ill members |  |  |  |  | |  |  |  | |  |  |
| Male only | 18.3  (1.2) | 16.6  (1.3) | 21.4  (1.0) | 13.9  (1.5) | | 11.1  (1.3) | 34.5  (1.6) | 20.1  (2.5) | | 20.8  (2.6) | 37.3  (2.2) |
|  |  |  |  |  | |  |  |  | |  |  |
| Female only | 17.1  (1.2) | 16.1  (1.3) | 20.8  (0.9) | 10.7  (1.2) | | 10.8  (1.1) | 33.4  (1.1) | 19.1  (2.3) | | 25.7  (1.9) | 41.8  (2.0) |
|  |  |  |  |  | |  |  |  | |  |  |
| Male and female | 28.9  (2.2) | 22.1  (1.9) | 30.8  (1.7) | 12.2  (2.0) | | 11.6  (1.4) | 39.4  (1.4) | 22.0  (1.3) | | 26.9  (1.4) | 43.8  (1.1) |
|  |  |  |  |  | |  |  |  | |  |  |
| Number of ill members |  |  |  |  | |  |  |  | |  |  |
| One | 17.6  (0.9) | 15.7  (1.0) | 20.5  (0.7) | 12.2  (1.0) | | 11.0  (0.9) | 33.1  (1.0) | 23.2  (2.7) | | 26.4  (2.6) | 41.7  (2.6) |
|  |  |  |  |  | |  |  |  | |  |  |
| Two or more | 25.0  (1.7) | 21.4  (1.5) | 28.8  (1.4) | 12.1  (1.9) | | 11.3  (1.4) | 40.6  (1.3) | 20.8  (1.1) | | 25.8  (1.2) | 42.8  (1.0) |
|  |  |  |  |  | |  |  |  | |  |  |
| Comorbidity of ill members |  |  |  |  | |  |  |  | |  |  |
| One disease (no comorbidity) | 18.1  (0.9) | 16.3  (0.9) | 22.7  (0.8) | 11.5  (0.9) | | 11.1  (0.8) | 33.2  (0.9) | 17.0  (1.4) | | 21.3  (1.8) | 38.6  (1.4) |
|  |  |  |  |  | |  |  |  | |  |  |
| Two or more diseases | 27.7  (2.2) | 29.3  (3.2) | 25.2  (1.8) | 41.8  (9.4) | | 11.2  (1.5) | 40.4  (1.3) | 24.5  (1.5) | | 28.5  (1.3) | 44.0  (1.2) |

NCD = noncommunicable diseases, OOP = out-of-pocket, n/o = no observations

Numbers in parentheses are standard errors
