# Supplementary material for: Financial risk protection against noncommunicable diseases: trends and patterns in Bangladesh
Source: BMC Public Health. 2022 Sep 30;22:1835. doi: 10.1186/s12889-022-14243-0 (PMC9524135; doi:10.1186/s12889-022-14243-0)
Supplement: Supplementary file 9 — Additional file 9. [file 12889_2022_14243_MOESM9_ESM.docx]

**Additional file 9:** Alternative calculation of the impoverishing effects of OOP expenditure (%), normative food, housing (rent), and utilities method

**Alternative measurement approach A** [using OOP expenses (as a separate variable and as a component of total consumption expenditure) from the survey’s health module]

| Impoverishment risk categories ^a^ | Households affected by non-NCD only | | | Households affected by NCD only | | | Households affected by both NCD and non-NCD | | |
| --- | --- | --- | --- | --- | --- | --- | --- | --- | --- |
|  | 2005  (n = 2,875) | 2010  (n = 2,931) | 2016  (n = 10,391) | 2005  (n = 1,648) | 2010  (n = 2, 449) | 2016  (n = 9,393) | 2005  (n=1,806) | 2010  (n = 2,440) | 2016  (n = 10,160) |
|  |  |  |  |  |  |  |  |  |  |
| 1. Further impoverished | 8.5  (0.5) | 9.3  (0.7) | 5.6  (0.4) | 1.2  (0.3) | 3.1  (0.4) | 5.9  (0.4) | 6.3  (0.6) | 6.4  (0.5) | 4.4  (0.3) |
|  |  |  |  |  |  |  |  |  |  |
| 1. Impoverished | 2.7  (0.3) | 3.1  (0.4) | 3.1  (0.2) | 1.4  (0.3) | 1.1  (0.2) | 3.5  (0.2) | 2.9  (0.4) | 2.8  (0.4) | 3.3  (0.2) |
|  |  |  |  |  |  |  |  |  |  |
| 1. At-risk of impoverishment ^b^ | 11.2  (0.6) | 12.0  (0.7) | 8.0  (0.4) | 3.3  (0.5) | 2.6  (0.4) | 7.5  (0.4) | 7.9  (0.7) | 9.2  (0.7) | 6.7  (0.3) |
|  |  |  |  |  |  |  |  |  |  |
| 1. Not at-risk of impoverishment | 67.9  (0.9) | 71.2  (1.2) | 72.4  (1.0) | 25.8  (1.2) | 25.6  (1.2) | 77.0  (0.7) | 75.7  (1.0) | 78.4  (1.1) | 82.2  (0.7) |
|  |  |  |  |  |  |  |  |  |  |
| 1. Non-spenders | 9.7  (0.6) | 4.4  (0.5) | 10.8  (0.9) | 68.3  (1.2) | 67.6  (1.3) | 6.1  (0.5) | 7.1  (0.6) | 3.2  (0.4) | 3.4  (0.5) |
|  |  |  |  |  |  |  |  |  |  |
| *Non-spenders disaggregated by reasons* |  |  |  |  |  |  |  |  |  |
|  |  |  |  |  |  |  |  |  |  |
| 5a. Financial reasons | 0.7  (0.2) | 0.2  (0.1) | 0.5  (0.1) | 0.9  (0.2) | 0.2  (0.1) | 0.3  (0.1) | 0.9  (0.2) | 0.2  (0.1) | 0.2  (0.1) |
|  |  |  |  |  |  |  |  |  |  |
| 5b. Non-financial reasons ^c^ | 7.7  (0.4) | 3.7  (0.3) | 6.3  (0.8) | 1.2  (0.3) | 0.9  (0.2) | 0.2  (0.1) | 4.2  (0.5) | 0.9  (0.2) | 2.1  (0.4) |
|  |  |  |  |  |  |  |  |  |  |
| 5c. Unspecified reasons ^d^ | 0.0  (n/o) | 0.0  (n/o) | 0.0  (n/o) | 66.0  (1.3) | 66.3  (1.3) | 5.0  (0.5) | 1.1  (0.3) | 1.6  (0.3) | 0.2  (0.1) |
|  |  |  |  |  |  |  |  |  |  |
| 5d. Non-spender but sought care | 1.2  (0.2) | 0.5  (0.2) | 4.0  (0.4) | 0.2  (0.1) | 0.2  (0.1) | 0.5  (0.1) | 1.0  (0.3) | 0.5  (0.2) | 0.9  (0.1) |

NCD = noncommunicable disease, CTP = capacity-to-pay, n/o = no observations

Numbers in parentheses are standard errors

^a^ The sum of the incidences of risk categories 1, 2, 3, 4, and 5 = 100%; the sum of the incidences of the risk categories 1, 2, 3, 4, 5a, 5b, 5c, and 5d = 100%

^b^ Households are at risk of impoverishment if consumption expenditure net of OOP expenses is between 100% and 120% of subsistence expenditure.

^c^ Non-financial reasons: health problem was not severe, distance, worried about receiving a fatal diagnosis, none to accompany, permission from the household decision-maker to seek care, didn’t know where to seek care, and others

^d^ Categories 5a, 5b, and 5c represent households forgoing care. HIES provides no information on reasons for forgoing care for individuals’ health problems within the last 12 months or illnesses that occurred 30 days before the survey but were ranked second or third in order of importance. HIES collects this information only for health problems ranked the most important within 30 days before the survey.

**Alternative measurement approach B** [using OOP expenses (as a separate variable) from the survey’s health module, and the OOP component of total consumption expenditure (thus CTP) from the consumption module]

| Impoverishment risk categories ^a^ | Household affected by non-NCD only | | | | Households affected by NCD only | | | | Households affected by both NCD and non-NCD | | | |
| --- | --- | --- | --- | --- | --- | --- | --- | --- | --- | --- | --- | --- |
|  | 2005  (n = 2,875) | 2010  (n = 2,931) | 2016  (n = 10,391) | 2005  (n = 1,648) | | 2010  (n = 2, 449) | 2016  (n = 9,393) | 2005  (n=1,806) | | 2010  (n = 2,440) | 2016  (n = 10,160) |  |
|  |  |  |  |  | |  |  |  | |  |  |  |
| 1. Further impoverished | 10.1  (0.6) | 10.3  (0.8) | 6.7  (0.4) | 2.0  (0.4) | | 3.4  (0.4) | 6.7  (0.4) | 7.3  (0.6) | | 7.3  (0.6) | 5.8  (0.4) |  |
|  |  |  |  |  | |  |  |  | |  |  |  |
| 1. Impoverished | 6.8  (0.5) | 5.7  (0.5) | 5.9  (0.3) | 3.6  (0.5) | | 3.3  (0.4) | 8.8  (0.4) | 5.6  (0.6) | | 5.5  (0.5) | 9.2  (0.4) |  |
|  |  |  |  |  | |  |  |  | |  |  |  |
| 1. At-risk of impoverishment ^b^ | 11.1  (0.6) | 11.5  (0.7) | 8.0  (0.4) | 2.7  (0.4) | | 2.5  (0.3) | 7.5  (0.4) | 8.7  (0.7) | | 9.2  (0.7) | 7.2  (0.3) |  |
|  |  |  |  |  | |  |  |  | |  |  |  |
| 1. Not at-risk of impoverishment | 62.4  (0.9) | 68.2  (1.2) | 68.5  (1.1) | 23.3  (1.1) | | 23.1  (1.1) | 70.8  (0.8) | 71.2  (1.1) | | 74.7  (1.1) | 74.4  (0.8) |  |
|  |  |  |  |  | |  |  |  | |  |  |  |
| 1. Non-spenders | 9.7  (0.6) | 4.4  (0.5) | 10.8  (0.9) | 68.3  (1.2) | | 67.6  (1.3) | 6.1  (0.5) | 7.1  (0.6) | | 3.2  (0.4) | 3.4  (0.5) |  |
|  |  |  |  |  | |  |  |  | |  |  |  |
| *Non-spenders disaggregated by reasons* |  |  |  |  | |  |  |  | |  |  |  |
|  |  |  |  |  | |  |  |  | |  |  |  |
| 5a. Financial reasons | 0.7  (0.2) | 0.2  (0.1) | 0.5  (0.1) | 0.9  (0.2) | | 0.2  (0.1) | 0.3  (0.1) | 0.9  (0.2) | | 0.2  (0.1) | 0.2  (0.1) |  |
|  |  |  |  |  | |  |  |  | |  |  |  |
| 5b. Non-financial reasons ^c^ | 7.7  (0.4) | 3.7  (0.3) | 6.3  (0.8) | 1.2  (0.3) | | 0.9  (0.2) | 0.2  (0.1) | 4.2  (0.5) | | 0.9  (0.2) | 2.1  (0.4) |  |
|  |  |  |  |  | |  |  |  | |  |  |  |
| 5c. Unspecified reasons ^d^ | 0.0  (n/o) | 0.0  (n/o) | 0.0  (n/o) | 66.0  (1.3) | | 66.3  (1.3) | 5.0  (0.5) | 1.1  (0.3) | | 1.6  (0.3) | 0.2  (0.1) |  |
|  |  |  |  |  | |  |  |  | |  |  |  |
| 5d. Non-spender but sought care | 1.2  (0.2) | 0.5  (0.2) | 4.0  (0.4) | 0.2  (0.1) | | 0.2  (0.1) | 0.5  (0.1) | 1.0  (0.3) | | 0.5  (0.2) | 0.9  (0.1) |  |

NCD = noncommunicable disease, CTP = capacity-to-pay, n/o = no observations

Numbers in parentheses are standard errors

^a^ The sum of the incidences of risk categories 1, 2, 3, 4, and 5 = 100%; the sum of the incidences of the risk categories 1, 2, 3, 4, 5a, 5b, 5c, and 5d = 100%

^b^ Households are at risk of impoverishment if consumption expenditure net of OOP expenses is between 100% and 120% of subsistence expenditure

^c^ Non-financial reasons: health problem was not severe, distance, worried about receiving a fatal diagnosis, none to accompany, permission from the household decision-maker to seek care, didn’t know where to seek care, and others

^d^ Categories 5a, 5b, and 5c represent households forgoing care. HIES provides no information on reasons for forgoing care for individuals’ health problems within the last 12 months or illnesses that occurred 30 days before the survey but were ranked second or third in order of importance. HIES collects this information only for health problems ranked the most important within 30 days before the survey.
