# Supplementary material for: Financial risk protection against noncommunicable diseases: trends and patterns in Bangladesh
Source: BMC Public Health. 2022 Sep 30;22:1835. doi: 10.1186/s12889-022-14243-0 (PMC9524135; doi:10.1186/s12889-022-14243-0)
Supplement: Supplementary file 10 — Additional file 10. [file 12889_2022_14243_MOESM10_ESM.docx]

**Additional file 10**: Alternative calculations of the incidence of impoverishment due to OOP expenses (%) among non-poor ^a^ households, normative food, housing (rent), and utilities method

**Alternative measurement approach A** [using OOP expenses (as a separate variable and as a component of total consumption expenditure) from the survey’s health module]

|  | Households affected by non-NCD only | | | Households affected by NCD only | | | Households affected by both NCD & non-NCD | | |
| --- | --- | --- | --- | --- | --- | --- | --- | --- | --- |
|  | 2005  (n = 2,875) | 2010  (n = 2,931) | 2016  (n = 10,391) | 2005  (n = 1,648) | 2010  (n = 2, 449) | 2016  (n = 9,393) | 2005  (n=1,806) | 2010  (n = 2,440) | 2016  (n = 10,160) |
|  |  |  |  |  |  |  |  |  |  |
| Overall | 2.7  (0.3) | 3.1  (0.4) | 3.1  (0.2) | 1.4  (0.3) | 1.1  (0.2) | 3.5  (0.2) | 2.9  (0.4) | 2.8  (0.4) | 3.3  (0.2) |
|  |  |  |  |  |  |  |  |  |  |
| Consumption expenditure quintile |  |  |  |  |  |  |  |  |  |
| Lowest ^b^ | 9.8  (1.3) | 11.6  (1.3) | 11.8  (0.8) | 5.0  (1.4) | 5.1  (1.1) | 13.8  (0.9) | 14.6  (2.3) | 15.4  (2.0) | 18.3  (1.2) |
|  |  |  |  |  |  |  |  |  |  |
| 2nd | 3.3  (0.8) | 2.0  (0.6) | 2.0  (0.3) | 0.4  (0.4) | 0.1  (0.1) | 2.4  (0.4) | 3.1  (1.1) | 1.5  (0.7) | 3.1  (0.5) |
|  |  |  |  |  |  |  |  |  |  |
| 3rd | 0.3  (0.2) | 0.2  (0.2) | 0.8  (0.2) | 1.5  (0.8) | 0.7  (0.4) | 1.8  (0.8) | 0.7  (0.5) | 0.8  (0.4) | 0.7  (0.2) |
|  |  |  |  |  |  |  |  |  |  |
| 4th | 0.0  (n/o) | 0.6  (0.4) | 0.1  (0.1) | 0.0  (n/o) | 0.0  (n/o) | 0.0  (n/o) | 0.0  (n/o) | 0.0  (n/o) | 0.3  (0.1) |
|  |  |  |  |  |  |  |  |  |  |
| Highest | 0.1  (0.1) | 0.0  (n/o) | 0.4  (0.2) | 0.6  (0.4) | 0.0  (n/o) | 0.6  (0.2) | 0.3  (0.3) | 0.1  (0.1) | 0.2  (0.1) |
|  |  |  |  |  |  |  |  |  |  |
| Area of residence |  |  |  |  |  |  |  |  |  |
| Rural | 3.1  (0.4) | 3.6  (0.4) | 3.8  (0.3) | 1.8  (0.4) | 1.4  (0.3) | 4.3  (0.3) | 3.5  (0.5) | 3.1  (0.4) | 3.9  (0.3) |
|  |  |  |  |  |  |  |  |  |  |
| Urban | 1.3  (0.3) | 1.3  (0.4) | 1.6  (0.4) | 0.2  (0.1) | 0.4  (0.1) | 1.0  (0.2) | 1.2  (0.4) | 1.5  (0.5) | 1.4  (0.3) |
|  |  |  |  |  |  |  |  |  |  |
| Household head's education |  |  |  |  |  |  |  |  |  |
| No education | 3.6  (0.5) | 3.7  (0.5) | 4.4  (0.4) | 2.2  (0.5) | 1.3  (0.3) | 5.4  (0.4) | 3.9  (0.7) | 4.1  (0.6) | 4.4  (0.4) |
|  |  |  |  |  |  |  |  |  |  |
| Below secondary | 2.1  (0.5) | 3.1  (0.6) | 2.8  (0.3) | 0.7  (0.4) | 1.3  (0.4) | 2.3  (0.3) | 2.5  (0.7) | 1.6  (0.5) | 3.1  (0.3) |
|  |  |  |  |  |  |  |  |  |  |
| Secondary or above | 0.2  (0.1) | 0.8  (0.4) | 0.3  (0.1) | 0.0  (n/o) | 0.0  (n/o) | 0.9  (0.2) | 0.5  (0.5) | 0.7  (0.5) | 0.4  (0.2) |
|  |  |  |  |  |  |  |  |  |  |
| Illness of main income earner |  |  |  |  |  |  |  |  |  |
| No | 2.3  (0.3) | 2.6  (0.4) | 2.6  (0.2) | 0.9  (0.3) | 1.1  (0.3) | 3.8  (0.4) | 2.8  (0.6) | 3.1  (0.6) | 3.0  (0.3) |
|  |  |  |  |  |  |  |  |  |  |
| Yes | 4.1  (0.8) | 4.4  (0.8) | 4.7  (0.6) | 1.9  (0.6) | 1.0  (0.3) | 3.0  (0.3) | 3.1  (0.6) | 2.5  (0.5) | 3.5  (0.3) |
|  |  |  |  |  |  |  |  |  |  |
| Age composition of ill members |  |  |  |  |  |  |  |  |  |
| Children (<18 years) only | 2.4  (0.4) | 2.9  (0.6) | 2.1  (0.3) | 1.8  (1.5) | 1.3  (1.0) | 6.3  (3.2) | 5.9  (3.1) | 2.9  (2.8) | 3.1  (1.0) |
|  |  |  |  |  |  |  |  |  |  |
| Non-elderly adults (18-60 years) only | 2.7  (0.6) | 2.9  (0.6) | 3.9  (0.4) | 1.5  (0.4) | 0.9  (0.2) | 2.6  (0.2) | 2.5  (0.8) | 3.2  (0.7) | 3.0  (0.4) |
|  |  |  |  |  |  |  |  |  |  |
| Elderly (>60 years) only | 3.9  (1.6) | 3.2  (1.7) | 4.3  (1.0) | 1.1  (0.7) | 0.9  (0.4) | 6.0  (0.6) | 3.5  (2.2) | 4.3  (1.7) | 9.7  (1.3) |
|  |  |  |  |  |  |  |  |  |  |
| Children and non-elderly adults | 3.6  (1.0) | 3.8  (0.9) | 3.8  (0.5) | 0.0  (n/o) | 1.4  (1.4) | 2.2  (0.8) | 3.0  (0.7) | 2.8  (0.6) | 3.0  (0.3) |
|  |  |  |  |  |  |  |  |  |  |
| Non-elderly adults and elderly | 0.0  (n/o) | 9.5  (5.0) | 1.7  (1.1) | 1.1  (1.1) | 2.1  (1.2) | 3.0  (0.6) | 4.2  (1.6) | 2.4  (1.0) | 2.9  (0.6) |
|  |  |  |  |  |  |  |  |  |  |
| Children and elderly | 0.0  (n/o) | 0.0  (n/o) | 3.1  (2.2) | 0.0  (n/o) | 13.7  (12.5) | 0.0  (n/o) | 2.6  (0.2) | 2.3  (1.3) | 1.2  (0.5) |
|  |  |  |  |  |  |  |  |  |  |
| Gender composition of ill members |  |  |  |  |  |  |  |  |  |
| Male only | 3.0  (0.5) | 3.1  (0.6) | 3.1  (0.4) | 1.1  (0.5) | 1.1  (0.4) | 2.8  (0.3) | 4.9  (1.3) | 3.1  (1.1) | 4.1  (0.7) |
|  |  |  |  |  |  |  |  |  |  |
| Female only | 2.1  (0.4) | 3.2  (0.6) | 2.8  (0.3) | 1.4  (0.5) | 0.9  (0.3) | 4.2  (0.5) | 3.7  (1.1) | 4.0  (0.9) | 4.5  (0.5) |
|  |  |  |  |  |  |  |  |  |  |
| Male and female | 3.5  (0.9) | 3.0  (0.8) | 3.8  (0.5) | 1.9  (0.8) | 1.3  (0.5) | 3.0  (0.4) | 2.2  (0.5) | 2.4  (0.4) | 2.7  (0.2) |
|  |  |  |  |  |  |  |  |  |  |
| Number of ill members |  |  |  |  |  |  |  |  |  |
| One | 2.5  (0.4) | 2.8  (0.4) | 3.1  (0.3) | 1.3  (0.3) | 1.1  (0.2) | 3.7  (0.3) | 4.5  (1.3) | 4.8  (1.2) | 5.6  (0.7) |
|  |  |  |  |  |  |  |  |  |  |
| Two or more | 3.2  (0.7) | 3.7  (0.7) | 3.3  (0.3) | 1.7  (0.7) | 1.1  (0.4) | 2.9  (0.3) | 2.7  (0.4) | 2.4  (0.4) | 2.8  (0.2) |
|  |  |  |  |  |  |  |  |  |  |
| Comorbidity of ill members |  |  |  |  |  |  |  |  |  |
| One disease (no comorbidity) | 2.6  (0.3) | 3.0  (0.4) | 3.1  (0.2) | 1.4  (0.3) | 1.0  (0.5) | 3.5  (0.3) | 2.5  (0.6) | 2.0  (0.5) | 2.8  (0.4) |
|  |  |  |  |  |  |  |  |  |  |
| Two or more diseases | 3.2  (0.9) | 4.0  (1.3) | 3.4  (0.5) | 0.0  (n/o) | 2.5  (0.4) | 3.2  (0.4) | 3.3  (0.6) | 3.3  (0.5) | 3.4  (0.3) |

NCD = noncommunicable diseases, OOP = out-of-pocket, CTP = capacity-to-pay, n/o = no observations

Numbers in parentheses are standard errors

^a^ A household is non-poor when its total consumption expenditure is equal to or more than subsistence expenditure. Subsistence expenditure is defined as the average per (equivalent) person spending on food, rent, and utilities of the households between the 25th and 35th percentiles of the per (equivalent) person total consumption expenditure distribution. A non-poor household is impoverished when its total consumption expenditure after paying for health care from out-of-pocket expenses is less than the household’s subsistence expenditure.

^b^ Some of the households in the poorest quintile are non-poor; so, besides the upper quintile households, the impoverishment incidence is available for the poorest quintile.

**Alternative measurement approach B** [using OOP expenses (as a separate variable) from the survey’s health module, and the OOP component of total consumption expenditure (thus CTP) from the consumption module]

|  | Households affected by non-NCD only | | | Households affected by NCD only | | | Households affected by both NCD & non-NCD | | |
| --- | --- | --- | --- | --- | --- | --- | --- | --- | --- |
|  | 2005  (n = 2,875) | 2010  (n = 2,931) | 2016  (n = 10,391) | 2005  (n = 1,648) | 2010  (n = 2, 449) | 2016  (n = 9,393) | 2005  (n=1,806) | 2010  (n = 2,440) | 2016  (n = 10,160) |
|  |  |  |  |  |  |  |  |  |  |
| Overall | 6.8  (0.5) | 5.7  (0.5) | 5.9  (0.3) | 3.6  (0.5) | 3.3  (0.4) | 8.8  (0.4) | 5.6  (0.6) | 5.5  (0.5) | 9.2  (0.4) |
|  |  |  |  |  |  |  |  |  |  |
| Consumption expenditure quintile |  |  |  |  |  |  |  |  |  |
| Lowest ^b^ | 15.3  (1.5) | 16.4  (1.6) | 16.9  (1.0) | 7.3  (1.7) | 6.5  (1.3) | 21.5  (1.1) | 19.4  (2.5) | 18.3  (2.0) | 26.9  (1.3) |
|  |  |  |  |  |  |  |  |  |  |
| 2nd | 9.5  (1.2) | 4.8  (0.9) | 5.9  (0.6) | 6.0  (1.5) | 6.1  (1.3) | 10.8  (1.0) | 7.2  (1.6) | 5.9  (1.1) | 13.5  (0.9) |
|  |  |  |  |  |  |  |  |  |  |
| 3rd | 3.0  (0.8) | 2.3  (0.7) | 2.9  (0.5) | 1.9  (0.7) | 3.8  (0.9) | 6.6  (0.6) | 2.2  (0.8) | 3.3  (0.9) | 6.6  (0.7) |
|  |  |  |  |  |  |  |  |  |  |
| 4th | 2.2  (0.7) | 1.5  (0.5) | 1.7  (0.4) | 1.9  (0.7) | 1.3  (0.6) | 3.6  (0.5) | 2.6  (0.9) | 2.1  (0.7) | 4.0  (0.6) |
|  |  |  |  |  |  |  |  |  |  |
| Highest | 1.9  (0.7) | 0.3  (0.3) | 0.6  (0.2) | 2.3  (0.9) | 0.9  (0.3) | 2.9  (0.5) | 1.3  (0.5) | 1.0  (0.4) | 1.6  (0.3) |
|  |  |  |  |  |  |  |  |  |  |
| Area of residence |  |  |  |  |  |  |  |  |  |
| Rural | 6.8  (0.6) | 6.4  (0.6) | 7.1  (0.4) | 3.6  (0.6) | 4.0  (0.5) | 10.2  (0.5) | 6.2  (0.7) | 6.2  (0.6) | 10.4  (0.5) |
|  |  |  |  |  |  |  |  |  |  |
| Urban | 6.6  (1.1) | 2.8  (0.7) | 3.2  (0.6) | 3.7  (0.9) | 1.8  (0.5) | 5.0  (0.6) | 4.0  (0.9) | 2.0  (0.5) | 5.5  (0.6) |
|  |  |  |  |  |  |  |  |  |  |
| Household head's education |  |  |  |  |  |  |  |  |  |
| No education | 7.7  (0.7) | 7.0  (0.8) | 7.1  (0.5) | 4.5  (0.7) | 4.2  (0.6) | 10.7  (0.6) | 6.8  (0.9) | 6.7  (0.8) | 10.0  (0.6) |
|  |  |  |  |  |  |  |  |  |  |
| Below secondary | 6.4  (0.9) | 4.9  (0.8) | 5.8  (0.4) | 3.0  (0.8) | 3.4  (0.7) | 8.2  (0.5) | 4.2  (0.9) | 4.6  (0.8) | 10.1  (0.6) |
|  |  |  |  |  |  |  |  |  |  |
| Secondary or above | 3.4  (1.1) | 2.2  (0.8) | 3.0  (0.6) | 1.9  (1.1) | 1.2  (0.5) | 5.4  (0.7) | 4.6  (1.5) | 2.5  (0.9) | 4.1  (0.6) |
|  |  |  |  |  |  |  |  |  |  |
| Illness of main income earner |  |  |  |  |  |  |  |  |  |
| No | 6.0  (0.5) | 5.9  (0.6) | 5.1  (0.3) | 3.9  (0.7) | 3.3  (0.5) | 9.2  (0.5) | 5.6  (0.9) | 7.1  (0.8) | 9.4  (0.6) |
|  |  |  |  |  |  |  |  |  |  |
| Yes | 9.3  (1.2) | 5.1  (0.8) | 8.3  (0.8) | 3.2  (0.7) | 3.4  (0.6) | 8.3  (0.5) | 5.6  (0.8) | 4.1  (0.6) | 9.0  (0.5) |
|  |  |  |  |  |  |  |  |  |  |
| Age composition of ill members |  |  |  |  |  |  |  |  |  |
| Children (<18 years) only | 4.6  (0.6) | 5.3  (0.7) | 4.2  (0.4) | 11.0  (3.7) | 5.3  (1.9) | 10.5  (3.1) | 8.7  (3.7) | 5.2  (3.5) | 5.6  (1.3) |
|  |  |  |  |  |  |  |  |  |  |
| Non-elderly adults (18-60 years) only | 8.2  (1.0) | 7.2  (0.9) | 6.5  (0.6) | 3.2  (0.6) | 3.2  (0.5) | 7.9  (0.5) | 6.0  (1.2) | 4.8  (0.8) | 8.4  (0.6) |
|  |  |  |  |  |  |  |  |  |  |
| Elderly (>60 years) only | 5.7  (1.8) | 2.7  (1.5) | 7.1  (1.3) | 2.5  (1.0) | 2.4  (0.7) | 11.6  (0.9) | 4.9  (2.7) | 6.8  (2.0) | 13.4  (1.6) |
|  |  |  |  |  |  |  |  |  |  |
| Children and non-elderly adults | 10.4  (1.6) | 4.6  (1.0) | 7.6  (0.8) | 4.7  (3.3) | 9.1  (3.4) | 8.2  (1.6) | 5.9  (0.9) | 5.2  (0.8) | 8.7  (0.6) |
|  |  |  |  |  |  |  |  |  |  |
| Non-elderly adults and elderly | 1.3  (1.3) | 0.0  (n/o) | 13.0  (3.8) | 4.1  (1.9) | 4.2  (1.7) | 9.0  (1.1) | 5.8  (1.8) | 8.3  (1.7) | 13.0  (1.3) |
|  |  |  |  |  |  |  |  |  |  |
| Children and elderly | 9.6  (0.6) | 2.1  (2.1) | 10.4  (5.3) | 0.0  (n/o) | 0.0  (n/o) | 7.4  (4.0) | 5.8  (0.6) | 3.4  (1.7) | 7.3  (1.4) |
|  |  |  |  |  |  |  |  |  |  |
| Gender composition of ill members |  |  |  |  |  |  |  |  |  |
| Male only | 7.1  (0.8) | 4.8  (0.7) | 5.5  (0.5) | 4.8  (1.0) | 3.8  (0.8) | 8.7  (0.6) | 9.3  (1.8) | 3.8  (1.1) | 8.9  (1.0) |
|  |  |  |  |  |  |  |  |  |  |
| Female only | 6.3  (0.8) | 6.1  (0.8) | 5.2  (0.4) | 3.1  (0.7) | 3.2  (0.6) | 8.8  (0.6) | 5.2  (1.3) | 6.8  (1.1) | 9.8  (0.8) |
|  |  |  |  |  |  |  |  |  |  |
| Male and female | 6.9  (1.2) | 6.5  (1.1) | 7.9  (0.7) | 2.5  (0.8) | 2.9  (0.7) | 9.1  (0.7) | 4.8  (0.7) | 5.3  (0.6) | 9.0  (0.5) |
|  |  |  |  |  |  |  |  |  |  |
| Number of ill members |  |  |  |  |  |  |  |  |  |
| One | 6.4  (0.6) | 5.8  (0.6) | 5.3  (0.4) | 3.9  (0.6) | 3.4  (0.4) | 8.5  (0.4) | 6.9  (1.6) | 6.1  (1.2) | 9.7  (0.9) |
|  |  |  |  |  |  |  |  |  |  |
| Two or more | 7.6  (1.0) | 5.3  (0.8) | 7.2  (0.6) | 2.7  (0.8) | 3.1  (0.7) | 9.6  (0.7) | 5.4  (0.6) | 5.3  (0.5) | 9.1  (0.4) |
|  |  |  |  |  |  |  |  |  |  |
| Comorbidity of ill members |  |  |  |  |  |  |  |  |  |
| One disease (no comorbidity) | 6.4  (0.5) | 5.2  (0.5) | 5.8  (0.3) | 3.6  (0.5) | 3.5  (0.4) | 8.9  (0.4) | 4.6  (0.8) | 4.8  (0.7) | 8.2  (0.6) |
|  |  |  |  |  |  |  |  |  |  |
| Two or more diseases | 8.6  (1.4) | 10.1  (2.0) | 6.3  (0.8) | 4.2  (3.4) | 2.9  (0.8) | 8.8  (0.7) | 6.5  (0.8) | 5.8  (0.6) | 9.5  (0.5) |

NCD = noncommunicable diseases, OOP = out-of-pocket, CTP = capacity-to-pay, n/o = no observations

Numbers in parentheses are standard errors

^a^ A household is non-poor when its total consumption expenditure is equal to or more than subsistence expenditure. Subsistence expenditure is defined as the average per (equivalent) person spending on food, rent, and utilities of the households between the 25th and 35th percentiles of the per (equivalent) person total consumption expenditure distribution. A non-poor household is impoverished when its total consumption expenditure after paying for health care from out-of-pocket expenses is less than the household’s subsistence expenditure.

^b^ Some of the households in the poorest quintile are non-poor; so, besides the upper quintile households, the impoverishment incidence is available for the poorest quintile.
